# Supplementary material for: Resolving the full spectrum of human genome variation using Linked-Reads
Source: Genome Res. 2019 Apr;29(4):635–45. doi: 10.1101/gr.234443.118 (PMC6442396; doi:10.1101/gr.234443.118)
Supplement: Supplemental Material [file supp_gr.234443.118_Supplemental_longranger-2.2.zip › longranger-2.2.tar.bz2!/lr2.2-public/lib/stan/cmdstan/stan/doc/api/html/namespacestan_1_1agrad.html]

Stan: stan::agrad Namespace Reference


|  |  |
| --- | --- |
|  | Stan  2.5.0  probability, sampling & optimization |


- Main Page
- Related Pages
- Namespaces
- Classes
- Files

- Namespace List
- Namespace Members

All Classes Namespaces Files Functions Variables Typedefs Enumerations Enumerator Friends Macros Pages

- stan
- agrad

Classes |
Typedefs |
Functions |
Variables

stan::agrad Namespace Reference

Function gradients via reverse-mode automatic differentiation.
More...

|  |  |
| --- | --- |
| Classes | |
| struct | fvar |
|  | |
| class | partials\_vari |
|  | |
| struct | OperandsAndPartials |
|  | A variable implementation that stores operands and derivatives with respect to the variable. More... |
|  | |
| class | chainable |
|  | Abstract base class for variable implementations that handles memory management and applying the chain rule. More... |
|  | |
| class | op\_ddv\_vari |
|  | |
| class | op\_dv\_vari |
|  | |
| class | op\_dvd\_vari |
|  | |
| class | op\_dvv\_vari |
|  | |
| class | op\_matrix\_vari |
|  | |
| class | precomp\_v\_vari |
|  | |
| class | precomputed\_gradients\_vari |
|  | This is a var implementation class that takes precomputed gradient values. More... |
|  | |
| class | op\_v\_vari |
|  | |
| class | op\_vd\_vari |
|  | |
| class | op\_vdd\_vari |
|  | |
| class | op\_vdv\_vari |
|  | |
| class | op\_vector\_vari |
|  | |
| class | op\_vv\_vari |
|  | |
| class | op\_vvd\_vari |
|  | |
| class | op\_vvv\_vari |
|  | |
| class | LDLT\_alloc |
|  | This object stores the actual (double typed) LDLT factorization of an Eigen::Matrix<var> along with pointers to its vari's which allow the \*\_ldlt functions to save memory. More... |
|  | |
| class | stored\_gradient\_vari |
|  | A var implementation that stores the daughter variable implementation pointers and the partial derivative with respect to the result explicitly in arrays constructed on the auto-diff memory stack. More... |
|  | |
| class | var |
|  | Independent (input) and dependent (output) variables for gradients. More... |
|  | |
| class | chainable\_alloc |
|  | A chainable\_alloc is an object which is constructed and destructed normally but the memory lifespan is managed along with the arena allocator for the gradient calculation. More... |
|  | |
| class | vari |
|  | The variable implementation base class. More... |
|  | |

|  |  |
| --- | --- |
| Typedefs | |
| typedef Eigen::Matrix< double,   Eigen::Dynamic, Eigen::Dynamic >  ::Index | size\_type |
|  | |
| typedef Eigen::Matrix< fvar  < double >, Eigen::Dynamic,   Eigen::Dynamic > | matrix\_fd |
|  | |
| typedef Eigen::Matrix< fvar  < var >, Eigen::Dynamic,   Eigen::Dynamic > | matrix\_fv |
|  | |
| typedef Eigen::Matrix< fvar  < fvar< double >  >, Eigen::Dynamic,   Eigen::Dynamic > | matrix\_ffd |
|  | |
| typedef Eigen::Matrix< fvar  < fvar< var >  >, Eigen::Dynamic,   Eigen::Dynamic > | matrix\_ffv |
|  | |
| typedef Eigen::Matrix< fvar  < double >, Eigen::Dynamic, 1 > | vector\_fd |
|  | |
| typedef Eigen::Matrix< fvar  < var >, Eigen::Dynamic, 1 > | vector\_fv |
|  | |
| typedef Eigen::Matrix< fvar  < fvar< double >  >, Eigen::Dynamic, 1 > | vector\_ffd |
|  | |
| typedef Eigen::Matrix< fvar  < fvar< var >  >, Eigen::Dynamic, 1 > | vector\_ffv |
|  | |
| typedef Eigen::Matrix< fvar  < double >, 1, Eigen::Dynamic > | row\_vector\_fd |
|  | |
| typedef Eigen::Matrix< fvar  < var >, 1, Eigen::Dynamic > | row\_vector\_fv |
|  | |
| typedef Eigen::Matrix< fvar  < fvar< double >  >, 1, Eigen::Dynamic > | row\_vector\_ffd |
|  | |
| typedef Eigen::Matrix< fvar  < fvar< var >  >, 1, Eigen::Dynamic > | row\_vector\_ffv |
|  | |
| typedef Eigen::Matrix< var,   Eigen::Dynamic, Eigen::Dynamic > | matrix\_v |
|  | The type of a matrix holding `stan::agrad::var` values. More... |
|  | |
| typedef Eigen::Matrix< var,   Eigen::Dynamic, 1 > | vector\_v |
|  | The type of a (column) vector holding `stan::agrad::var` values. More... |
|  | |
| typedef Eigen::Matrix< var,   1, Eigen::Dynamic > | row\_vector\_v |
|  | The type of a row vector holding `stan::agrad::var` values. More... |
|  | |

|  |  |
| --- | --- |
| Functions | |
| template<typename T , typename F > | |
| void | derivative (const F &f, const T &x, T &fx, T &dfx\_dx) |
|  | Return the derivative of the specified univariate function at the specified argument. More... |
|  | |
| template<typename T , typename F > | |
| void | partial\_derivative (const F &f, const Eigen::Matrix< T, Eigen::Dynamic, 1 > &x, int n, T &fx, T &dfx\_dxn) |
|  | Return the partial derivative of the specified multiivariate function at the specified argument. More... |
|  | |
| template<typename F > | |
| void | gradient (const F &f, const Eigen::Matrix< double, Eigen::Dynamic, 1 > &x, double &fx, Eigen::Matrix< double, Eigen::Dynamic, 1 > &grad\_fx) |
|  | Calculate the value and the gradient of the specified function at the specified argument. More... |
|  | |
| template<typename T , typename F > | |
| void | gradient (const F &f, const Eigen::Matrix< T, Eigen::Dynamic, 1 > &x, T &fx, Eigen::Matrix< T, Eigen::Dynamic, 1 > &grad\_fx) |
|  | |
| template<typename F > | |
| void | jacobian (const F &f, const Eigen::Matrix< double, Eigen::Dynamic, 1 > &x, Eigen::Matrix< double, Eigen::Dynamic, 1 > &fx, Eigen::Matrix< double, Eigen::Dynamic, Eigen::Dynamic > &J) |
|  | |
| template<typename T , typename F > | |
| void | jacobian (const F &f, const Eigen::Matrix< T, Eigen::Dynamic, 1 > &x, Eigen::Matrix< T, Eigen::Dynamic, 1 > &fx, Eigen::Matrix< T, Eigen::Dynamic, Eigen::Dynamic > &J) |
|  | |
| template<typename F > | |
| void | hessian (const F &f, const Eigen::Matrix< double, Eigen::Dynamic, 1 > &x, double &fx, Eigen::Matrix< double, Eigen::Dynamic, 1 > &grad, Eigen::Matrix< double, Eigen::Dynamic, Eigen::Dynamic > &H) |
|  | |
| template<typename T , typename F > | |
| void | hessian (const F &f, const Eigen::Matrix< T, Eigen::Dynamic, 1 > &x, T &fx, Eigen::Matrix< T, Eigen::Dynamic, 1 > &grad, Eigen::Matrix< T, Eigen::Dynamic, Eigen::Dynamic > &H) |
|  | |
| template<typename T1 , typename T2 , typename F > | |
| void | gradient\_dot\_vector (const F &f, const Eigen::Matrix< T1, Eigen::Dynamic, 1 > &x, const Eigen::Matrix< T2, Eigen::Dynamic, 1 > &v, T1 &fx, T1 &grad\_fx\_dot\_v) |
|  | |
| template<typename F > | |
| void | hessian\_times\_vector (const F &f, const Eigen::Matrix< double, Eigen::Dynamic, 1 > &x, const Eigen::Matrix< double, Eigen::Dynamic, 1 > &v, double &fx, Eigen::Matrix< double, Eigen::Dynamic, 1 > &Hv) |
|  | |
| template<typename T , typename F > | |
| void | hessian\_times\_vector (const F &f, const Eigen::Matrix< T, Eigen::Dynamic, 1 > &x, const Eigen::Matrix< T, Eigen::Dynamic, 1 > &v, T &fx, Eigen::Matrix< T, Eigen::Dynamic, 1 > &Hv) |
|  | |
| template<typename F > | |
| void | grad\_tr\_mat\_times\_hessian (const F &f, const Eigen::Matrix< double, Eigen::Dynamic, 1 > &x, const Eigen::Matrix< double, Eigen::Dynamic, Eigen::Dynamic > &M, Eigen::Matrix< double, Eigen::Dynamic, 1 > &grad\_tr\_MH) |
|  | |
| template<typename T > | |
| fvar< T > | abs (const fvar< T > &x) |
|  | |
| template<typename T > | |
| fvar< T > | acos (const fvar< T > &x) |
|  | |
| template<typename T > | |
| fvar< T > | acosh (const fvar< T > &x) |
|  | |
| template<typename T > | |
| fvar< T > | asin (const fvar< T > &x) |
|  | |
| template<typename T > | |
| fvar< T > | asinh (const fvar< T > &x) |
|  | |
| template<typename T > | |
| fvar< T > | atan (const fvar< T > &x) |
|  | |
| template<typename T > | |
| fvar< T > | atan2 (const fvar< T > &x1, const fvar< T > &x2) |
|  | |
| template<typename T > | |
| fvar< T > | atan2 (const double x1, const fvar< T > &x2) |
|  | |
| template<typename T > | |
| fvar< T > | atan2 (const fvar< T > &x1, const double x2) |
|  | |
| template<typename T > | |
| fvar< T > | atanh (const fvar< T > &x) |
|  | |
| template<typename T > | |
| fvar< T > | bessel\_first\_kind (int v, const fvar< T > &z) |
|  | |
| template<typename T > | |
| fvar< T > | bessel\_second\_kind (int v, const fvar< T > &z) |
|  | |
| template<typename T > | |
| fvar< T > | binary\_log\_loss (const int y, const fvar< T > &y\_hat) |
|  | |
| template<typename T > | |
| fvar< T > | binomial\_coefficient\_log (const fvar< T > &x1, const fvar< T > &x2) |
|  | |
| template<typename T > | |
| fvar< T > | binomial\_coefficient\_log (const fvar< T > &x1, const double x2) |
|  | |
| template<typename T > | |
| fvar< T > | binomial\_coefficient\_log (const double x1, const fvar< T > &x2) |
|  | |
| template<typename T > | |
| fvar< T > | cbrt (const fvar< T > &x) |
|  | |
| template<typename T > | |
| fvar< T > | ceil (const fvar< T > &x) |
|  | |
| template<typename T > | |
| fvar< T > | cos (const fvar< T > &x) |
|  | |
| template<typename T > | |
| fvar< T > | cosh (const fvar< T > &x) |
|  | |
| template<typename T > | |
| fvar< T > | digamma (const fvar< T > &x) |
|  | |
| template<typename T > | |
| fvar< T > | erf (const fvar< T > &x) |
|  | |
| template<typename T > | |
| fvar< T > | erfc (const fvar< T > &x) |
|  | |
| template<typename T > | |
| fvar< T > | exp (const fvar< T > &x) |
|  | |
| template<typename T > | |
| fvar< T > | exp2 (const fvar< T > &x) |
|  | |
| template<typename T > | |
| fvar< T > | expm1 (const fvar< T > &x) |
|  | |
| template<typename T > | |
| fvar< T > | fabs (const fvar< T > &x) |
|  | |
| template<typename T > | |
| fvar< T > | falling\_factorial (const fvar< T > &x, const fvar< T > &n) |
|  | |
| template<typename T > | |
| fvar< T > | falling\_factorial (const fvar< T > &x, const double n) |
|  | |
| template<typename T > | |
| fvar< T > | falling\_factorial (const double x, const fvar< T > &n) |
|  | |
| template<typename T > | |
| fvar< T > | fdim (const fvar< T > &x1, const fvar< T > &x2) |
|  | |
| template<typename T > | |
| fvar< T > | fdim (const fvar< T > &x1, const double x2) |
|  | |
| template<typename T > | |
| fvar< T > | fdim (const double x1, const fvar< T > &x2) |
|  | |
| template<typename T > | |
| fvar< T > | floor (const fvar< T > &x) |
|  | |
| template<typename T1 , typename T2 , typename T3 > | |
| fvar< typename   stan::return\_type< T1, T2, T3 >  ::type > | fma (const fvar< T1 > &x1, const fvar< T2 > &x2, const fvar< T3 > &x3) |
|  | The fused multiply-add operation (C99). More... |
|  | |
| template<typename T1 , typename T2 , typename T3 > | |
| fvar< typename   stan::return\_type< T1, T2, T3 >  ::type > | fma (const T1 &x1, const fvar< T2 > &x2, const fvar< T3 > &x3) |
|  | See all-var input signature for details on the function and derivatives. More... |
|  | |
| template<typename T1 , typename T2 , typename T3 > | |
| fvar< typename   stan::return\_type< T1, T2, T3 >  ::type > | fma (const fvar< T1 > &x1, const T2 &x2, const fvar< T3 > &x3) |
|  | See all-var input signature for details on the function and derivatives. More... |
|  | |
| template<typename T1 , typename T2 , typename T3 > | |
| fvar< typename   stan::return\_type< T1, T2, T3 >  ::type > | fma (const fvar< T1 > &x1, const fvar< T2 > &x2, const T3 &x3) |
|  | See all-var input signature for details on the function and derivatives. More... |
|  | |
| template<typename T1 , typename T2 , typename T3 > | |
| fvar< typename   stan::return\_type< T1, T2, T3 >  ::type > | fma (const T1 &x1, const T2 &x2, const fvar< T3 > &x3) |
|  | See all-var input signature for details on the function and derivatives. More... |
|  | |
| template<typename T1 , typename T2 , typename T3 > | |
| fvar< typename   stan::return\_type< T1, T2, T3 >  ::type > | fma (const fvar< T1 > &x1, const T2 &x2, const T3 &x3) |
|  | See all-var input signature for details on the function and derivatives. More... |
|  | |
| template<typename T1 , typename T2 , typename T3 > | |
| fvar< typename   stan::return\_type< T1, T2, T3 >  ::type > | fma (const T1 &x1, const fvar< T2 > &x2, const T3 &x3) |
|  | See all-var input signature for details on the function and derivatives. More... |
|  | |
| template<typename T > | |
| fvar< T > | fmax (const fvar< T > &x1, const fvar< T > &x2) |
|  | |
| template<typename T > | |
| fvar< T > | fmax (const double x1, const fvar< T > &x2) |
|  | |
| template<typename T > | |
| fvar< T > | fmax (const fvar< T > &x1, const double x2) |
|  | |
| template<typename T > | |
| fvar< T > | fmin (const fvar< T > &x1, const fvar< T > &x2) |
|  | |
| template<typename T > | |
| fvar< T > | fmin (const double x1, const fvar< T > &x2) |
|  | |
| template<typename T > | |
| fvar< T > | fmin (const fvar< T > &x1, const double x2) |
|  | |
| template<typename T > | |
| fvar< T > | fmod (const fvar< T > &x1, const fvar< T > &x2) |
|  | |
| template<typename T > | |
| fvar< T > | fmod (const fvar< T > &x1, const double x2) |
|  | |
| template<typename T > | |
| fvar< T > | fmod (const double x1, const fvar< T > &x2) |
|  | |
| template<typename T > | |
| fvar< T > | gamma\_p (const fvar< T > &x1, const fvar< T > &x2) |
|  | |
| template<typename T > | |
| fvar< T > | gamma\_p (const fvar< T > &x1, const double x2) |
|  | |
| template<typename T > | |
| fvar< T > | gamma\_p (const double x1, const fvar< T > &x2) |
|  | |
| template<typename T > | |
| fvar< T > | gamma\_q (const fvar< T > &x1, const fvar< T > &x2) |
|  | |
| template<typename T > | |
| fvar< T > | gamma\_q (const fvar< T > &x1, const double x2) |
|  | |
| template<typename T > | |
| fvar< T > | gamma\_q (const double x1, const fvar< T > &x2) |
|  | |
| template<typename T > | |
| fvar< T > | hypot (const fvar< T > &x1, const fvar< T > &x2) |
|  | |
| template<typename T > | |
| fvar< T > | hypot (const fvar< T > &x1, const double x2) |
|  | |
| template<typename T > | |
| fvar< T > | hypot (const double x1, const fvar< T > &x2) |
|  | |
| template<typename T > | |
| fvar< T > | inv (const fvar< T > &x) |
|  | |
| template<typename T > | |
| fvar< T > | inv\_cloglog (const fvar< T > &x) |
|  | |
| template<typename T > | |
| fvar< T > | inv\_logit (const fvar< T > &x) |
|  | |
| template<typename T > | |
| fvar< T > | inv\_sqrt (const fvar< T > &x) |
|  | |
| template<typename T > | |
| fvar< T > | inv\_square (const fvar< T > &x) |
|  | |
| template<typename T > | |
| int | is\_inf (const fvar< T > &x) |
|  | Returns 1 if the input's value is infinite and 0 otherwise. More... |
|  | |
| template<typename T > | |
| int | is\_nan (const fvar< T > &x) |
|  | Returns 1 if the input's value is NaN and 0 otherwise. More... |
|  | |
| template<typename T > | |
| fvar< T > | lbeta (const fvar< T > &x1, const fvar< T > &x2) |
|  | |
| template<typename T > | |
| fvar< T > | lbeta (const double x1, const fvar< T > &x2) |
|  | |
| template<typename T > | |
| fvar< T > | lbeta (const fvar< T > &x1, const double x2) |
|  | |
| template<typename T > | |
| fvar< T > | lgamma (const fvar< T > &x) |
|  | |
| template<typename T > | |
| fvar< typename   stan::return\_type< T, int >  ::type > | lmgamma (int x1, const fvar< T > &x2) |
|  | |
| template<typename T > | |
| fvar< T > | log (const fvar< T > &x) |
|  | |
| template<typename T > | |
| fvar< T > | log10 (const fvar< T > &x) |
|  | |
| template<typename T > | |
| fvar< T > | log1m (const fvar< T > &x) |
|  | |
| template<typename T > | |
| fvar< T > | log1m\_exp (const fvar< T > &x) |
|  | |
| template<typename T > | |
| fvar< T > | log1m\_inv\_logit (const fvar< T > &x) |
|  | |
| template<typename T > | |
| fvar< T > | log1p (const fvar< T > &x) |
|  | |
| template<typename T > | |
| fvar< T > | log1p\_exp (const fvar< T > &x) |
|  | |
| template<typename T > | |
| fvar< T > | log2 (const fvar< T > &x) |
|  | |
| template<typename T > | |
| fvar< T > | log\_diff\_exp (const fvar< T > &x1, const fvar< T > &x2) |
|  | |
| template<typename T1 , typename T2 > | |
| fvar< T2 > | log\_diff\_exp (const T1 &x1, const fvar< T2 > &x2) |
|  | |
| template<typename T1 , typename T2 > | |
| fvar< T1 > | log\_diff\_exp (const fvar< T1 > &x1, const T2 &x2) |
|  | |
| template<typename T > | |
| fvar< T > | log\_falling\_factorial (const fvar< T > &x, const fvar< T > &n) |
|  | |
| template<typename T > | |
| fvar< T > | log\_falling\_factorial (const double x, const fvar< T > &n) |
|  | |
| template<typename T > | |
| fvar< T > | log\_falling\_factorial (const fvar< T > &x, const double n) |
|  | |
| template<typename T > | |
| fvar< T > | log\_inv\_logit (const fvar< T > &x) |
|  | |
| template<typename T > | |
| fvar< T > | log\_rising\_factorial (const fvar< T > &x, const fvar< T > &n) |
|  | |
| template<typename T > | |
| fvar< T > | log\_rising\_factorial (const fvar< T > &x, const double n) |
|  | |
| template<typename T > | |
| fvar< T > | log\_rising\_factorial (const double x, const fvar< T > &n) |
|  | |
| template<typename T > | |
| fvar< T > | log\_sum\_exp (const fvar< T > &x1, const fvar< T > &x2) |
|  | |
| template<typename T > | |
| fvar< T > | log\_sum\_exp (const double x1, const fvar< T > &x2) |
|  | |
| template<typename T > | |
| fvar< T > | log\_sum\_exp (const fvar< T > &x1, const double x2) |
|  | |
| template<typename T > | |
| fvar< T > | log\_sum\_exp (const std::vector< fvar< T > > &v) |
|  | |
| template<typename T > | |
| fvar< T > | logit (const fvar< T > &x) |
|  | |
| template<typename T > | |
| fvar< T > | modified\_bessel\_first\_kind (int v, const fvar< T > &z) |
|  | |
| template<typename T > | |
| fvar< T > | modified\_bessel\_second\_kind (int v, const fvar< T > &z) |
|  | |
| template<typename T > | |
| fvar< T > | multiply\_log (const fvar< T > &x1, const fvar< T > &x2) |
|  | |
| template<typename T > | |
| fvar< T > | multiply\_log (const double x1, const fvar< T > &x2) |
|  | |
| template<typename T > | |
| fvar< T > | multiply\_log (const fvar< T > &x1, const double x2) |
|  | |
| template<typename T > | |
| fvar< T > | owens\_t (const fvar< T > &x1, const fvar< T > &x2) |
|  | |
| template<typename T > | |
| fvar< T > | owens\_t (const double x1, const fvar< T > &x2) |
|  | |
| template<typename T > | |
| fvar< T > | owens\_t (const fvar< T > &x1, const double x2) |
|  | |
| template<typename T > | |
| fvar< T > | Phi (const fvar< T > &x) |
|  | |
| template<typename T > | |
| fvar< T > | pow (const fvar< T > &x1, const fvar< T > &x2) |
|  | |
| template<typename T > | |
| fvar< T > | pow (const double x1, const fvar< T > &x2) |
|  | |
| template<typename T > | |
| fvar< T > | pow (const fvar< T > &x1, const double x2) |
|  | |
| template<typename T > | |
| double | primitive\_value (const fvar< T > &v) |
|  | Return the primitive value of the specified forward-mode autodiff variable. More... |
|  | |
| template<typename T > | |
| fvar< T > | rising\_factorial (const fvar< T > &x, const fvar< T > &n) |
|  | |
| template<typename T > | |
| fvar< T > | rising\_factorial (const fvar< T > &x, const double n) |
|  | |
| template<typename T > | |
| fvar< T > | rising\_factorial (const double x, const fvar< T > &n) |
|  | |
| template<typename T > | |
| fvar< T > | round (const fvar< T > &x) |
|  | |
| template<typename T > | |
| fvar< T > | sin (const fvar< T > &x) |
|  | |
| template<typename T > | |
| fvar< T > | sinh (const fvar< T > &x) |
|  | |
| template<typename T > | |
| fvar< T > | sqrt (const fvar< T > &x) |
|  | |
| template<typename T > | |
| fvar< T > | square (const fvar< T > &x) |
|  | |
| template<typename T > | |
| fvar< T > | tan (const fvar< T > &x) |
|  | |
| template<typename T > | |
| fvar< T > | tanh (const fvar< T > &x) |
|  | |
| template<typename T > | |
| fvar< T > | tgamma (const fvar< T > &x) |
|  | |
| template<typename T > | |
| fvar< T > | trunc (const fvar< T > &x) |
|  | |
| template<typename T > | |
| T | value\_of (const fvar< T > &v) |
|  | Return the value of the specified variable. More... |
|  | |
| template<typename T , int R1, int C1, int R2, int C2> | |
| Eigen::Matrix< fvar< T >, 1, C1 > | columns\_dot\_product (const Eigen::Matrix< fvar< T >, R1, C1 > &v1, const Eigen::Matrix< fvar< T >, R2, C2 > &v2) |
|  | |
| template<typename T , int R1, int C1, int R2, int C2> | |
| Eigen::Matrix< fvar< T >, 1, C1 > | columns\_dot\_product (const Eigen::Matrix< fvar< T >, R1, C1 > &v1, const Eigen::Matrix< double, R2, C2 > &v2) |
|  | |
| template<typename T , int R1, int C1, int R2, int C2> | |
| Eigen::Matrix< fvar< T >, 1, C1 > | columns\_dot\_product (const Eigen::Matrix< double, R1, C1 > &v1, const Eigen::Matrix< fvar< T >, R2, C2 > &v2) |
|  | |
| template<typename T , int R, int C> | |
| Eigen::Matrix< fvar< T >, 1, C > | columns\_dot\_self (const Eigen::Matrix< fvar< T >, R, C > &x) |
|  | |
| template<typename T , int R, int C> | |
| Eigen::Matrix< fvar< T >, C, C > | crossprod (const Eigen::Matrix< fvar< T >, R, C > &m) |
|  | |
| template<typename T , int R, int C> | |
| fvar< T > | determinant (const Eigen::Matrix< fvar< T >, R, C > &m) |
|  | |
| template<typename T1 , typename T2 > | |
| stan::return\_type< T1, T2 >::type | divide (const T1 &v, const T2 &c) |
|  | |
| template<typename T , int R, int C> | |
| Eigen::Matrix< fvar< T >, R, C > | divide (const Eigen::Matrix< fvar< T >, R, C > &v, const fvar< T > &c) |
|  | |
| template<typename T , int R, int C> | |
| Eigen::Matrix< fvar< T >, R, C > | divide (const Eigen::Matrix< fvar< T >, R, C > &v, const double c) |
|  | |
| template<typename T , int R, int C> | |
| Eigen::Matrix< fvar< T >, R, C > | divide (const Eigen::Matrix< double, R, C > &v, const fvar< T > &c) |
|  | |
| template<typename T , int R, int C> | |
| Eigen::Matrix< fvar< T >, R, C > | operator/ (const Eigen::Matrix< fvar< T >, R, C > &v, const fvar< T > &c) |
|  | |
| template<typename T , int R, int C> | |
| Eigen::Matrix< fvar< T >, R, C > | operator/ (const Eigen::Matrix< fvar< T >, R, C > &v, const double c) |
|  | |
| template<typename T , int R, int C> | |
| Eigen::Matrix< fvar< T >, R, C > | operator/ (const Eigen::Matrix< double, R, C > &v, const fvar< T > &c) |
|  | |
| template<typename T , int R1, int C1, int R2, int C2> | |
| fvar< T > | dot\_product (const Eigen::Matrix< fvar< T >, R1, C1 > &v1, const Eigen::Matrix< fvar< T >, R2, C2 > &v2) |
|  | |
| template<typename T , int R1, int C1, int R2, int C2> | |
| fvar< T > | dot\_product (const Eigen::Matrix< fvar< T >, R1, C1 > &v1, const Eigen::Matrix< double, R2, C2 > &v2) |
|  | |
| template<typename T , int R1, int C1, int R2, int C2> | |
| fvar< T > | dot\_product (const Eigen::Matrix< double, R1, C1 > &v1, const Eigen::Matrix< fvar< T >, R2, C2 > &v2) |
|  | |
| template<typename T , int R1, int C1, int R2, int C2> | |
| fvar< T > | dot\_product (const Eigen::Matrix< fvar< T >, R1, C1 > &v1, const Eigen::Matrix< fvar< T >, R2, C2 > &v2, size\_type &length) |
|  | |
| template<typename T , int R1, int C1, int R2, int C2> | |
| fvar< T > | dot\_product (const Eigen::Matrix< fvar< T >, R1, C1 > &v1, const Eigen::Matrix< double, R2, C2 > &v2, size\_type &length) |
|  | |
| template<typename T , int R1, int C1, int R2, int C2> | |
| fvar< T > | dot\_product (const Eigen::Matrix< double, R1, C1 > &v1, const Eigen::Matrix< fvar< T >, R2, C2 > &v2, size\_type &length) |
|  | |
| template<typename T > | |
| fvar< T > | dot\_product (const std::vector< fvar< T > > &v1, const std::vector< fvar< T > > &v2) |
|  | |
| template<typename T > | |
| fvar< T > | dot\_product (const std::vector< double > &v1, const std::vector< fvar< T > > &v2) |
|  | |
| template<typename T > | |
| fvar< T > | dot\_product (const std::vector< fvar< T > > &v1, const std::vector< double > &v2) |
|  | |
| template<typename T > | |
| fvar< T > | dot\_product (const std::vector< fvar< T > > &v1, const std::vector< fvar< T > > &v2, size\_type &length) |
|  | |
| template<typename T > | |
| fvar< T > | dot\_product (const std::vector< double > &v1, const std::vector< fvar< T > > &v2, size\_type &length) |
|  | |
| template<typename T > | |
| fvar< T > | dot\_product (const std::vector< fvar< T > > &v1, const std::vector< double > &v2, size\_type &length) |
|  | |
| template<typename T , int R, int C> | |
| fvar< T > | dot\_self (const Eigen::Matrix< fvar< T >, R, C > &v) |
|  | |
| template<typename T , int R, int C> | |
| Eigen::Matrix< fvar< T >, R, C > | inverse (const Eigen::Matrix< fvar< T >, R, C > &m) |
|  | |
| template<typename T , int R, int C> | |
| fvar< T > | log\_determinant (const Eigen::Matrix< fvar< T >, R, C > &m) |
|  | |
| template<typename T > | |
| Eigen::Matrix< fvar< T >  , Eigen::Dynamic, 1 > | log\_softmax (const Eigen::Matrix< fvar< T >, Eigen::Dynamic, 1 > &alpha) |
|  | |
| template<typename T , int R, int C> | |
| fvar< T > | log\_sum\_exp (const Eigen::Matrix< fvar< T >, R, C > &v) |
|  | |
| template<typename T , int R1, int C1, int R2, int C2> | |
| Eigen::Matrix< fvar< T >, R1, C2 > | mdivide\_left (const Eigen::Matrix< fvar< T >, R1, C1 > &A, const Eigen::Matrix< fvar< T >, R2, C2 > &b) |
|  | |
| template<typename T , int R1, int C1, int R2, int C2> | |
| Eigen::Matrix< fvar< T >, R1, C2 > | mdivide\_left (const Eigen::Matrix< double, R1, C1 > &A, const Eigen::Matrix< fvar< T >, R2, C2 > &b) |
|  | |
| template<typename T , int R1, int C1, int R2, int C2> | |
| Eigen::Matrix< fvar< T >, R1, C2 > | mdivide\_left (const Eigen::Matrix< fvar< T >, R1, C1 > &A, const Eigen::Matrix< double, R2, C2 > &b) |
|  | |
| template<int R1, int C1, int R2, int C2, typename T2 > | |
| Eigen::Matrix< fvar< T2 >, R1, C2 > | mdivide\_left\_ldlt (const stan::math::LDLT\_factor< double, R1, C1 > &A, const Eigen::Matrix< fvar< T2 >, R2, C2 > &b) |
|  | Returns the solution of the system Ax=b given an LDLT\_factor of A. More... |
|  | |
| template<typename T , int R1, int C1, int R2, int C2> | |
| Eigen::Matrix< fvar< T >, R1, C1 > | mdivide\_left\_tri\_low (const Eigen::Matrix< fvar< T >, R1, C1 > &A, const Eigen::Matrix< fvar< T >, R2, C2 > &b) |
|  | |
| template<typename T , int R1, int C1, int R2, int C2> | |
| Eigen::Matrix< fvar< T >, R1, C1 > | mdivide\_left\_tri\_low (const Eigen::Matrix< double, R1, C1 > &A, const Eigen::Matrix< fvar< T >, R2, C2 > &b) |
|  | |
| template<typename T , int R1, int C1, int R2, int C2> | |
| Eigen::Matrix< fvar< T >, R1, C1 > | mdivide\_left\_tri\_low (const Eigen::Matrix< fvar< T >, R1, C1 > &A, const Eigen::Matrix< double, R2, C2 > &b) |
|  | |
| template<typename T , int R1, int C1, int R2, int C2> | |
| Eigen::Matrix< fvar< T >, R1, C2 > | mdivide\_right (const Eigen::Matrix< fvar< T >, R1, C1 > &A, const Eigen::Matrix< fvar< T >, R2, C2 > &b) |
|  | |
| template<typename T , int R1, int C1, int R2, int C2> | |
| Eigen::Matrix< fvar< T >, R1, C2 > | mdivide\_right (const Eigen::Matrix< fvar< T >, R1, C1 > &A, const Eigen::Matrix< double, R2, C2 > &b) |
|  | |
| template<typename T , int R1, int C1, int R2, int C2> | |
| Eigen::Matrix< fvar< T >, R1, C2 > | mdivide\_right (const Eigen::Matrix< double, R1, C1 > &A, const Eigen::Matrix< fvar< T >, R2, C2 > &b) |
|  | |
| template<typename T , int R1, int C1, int R2, int C2> | |
| Eigen::Matrix< fvar< T >, R1, C1 > | mdivide\_right\_tri\_low (const Eigen::Matrix< fvar< T >, R1, C1 > &A, const Eigen::Matrix< fvar< T >, R2, C2 > &b) |
|  | |
| template<typename T , int R1, int C1, int R2, int C2> | |
| Eigen::Matrix< fvar< T >, R1, C2 > | mdivide\_right\_tri\_low (const Eigen::Matrix< fvar< T >, R1, C1 > &A, const Eigen::Matrix< double, R2, C2 > &b) |
|  | |
| template<typename T , int R1, int C1, int R2, int C2> | |
| Eigen::Matrix< fvar< T >, R1, C2 > | mdivide\_right\_tri\_low (const Eigen::Matrix< double, R1, C1 > &A, const Eigen::Matrix< fvar< T >, R2, C2 > &b) |
|  | |
| template<typename T , int R1, int C1> | |
| Eigen::Matrix< fvar< T >, R1, C1 > | multiply (const Eigen::Matrix< fvar< T >, R1, C1 > &m, const fvar< T > &c) |
|  | |
| template<typename T , int R2, int C2> | |
| Eigen::Matrix< fvar< T >, R2, C2 > | multiply (const Eigen::Matrix< fvar< T >, R2, C2 > &m, const double c) |
|  | |
| template<typename T , int R1, int C1> | |
| Eigen::Matrix< fvar< T >, R1, C1 > | multiply (const Eigen::Matrix< double, R1, C1 > &m, const fvar< T > &c) |
|  | |
| template<typename T , int R1, int C1> | |
| Eigen::Matrix< fvar< T >, R1, C1 > | multiply (const fvar< T > &c, const Eigen::Matrix< fvar< T >, R1, C1 > &m) |
|  | |
| template<typename T , int R1, int C1> | |
| Eigen::Matrix< fvar< T >, R1, C1 > | multiply (const double c, const Eigen::Matrix< fvar< T >, R1, C1 > &m) |
|  | |
| template<typename T , int R1, int C1> | |
| Eigen::Matrix< fvar< T >, R1, C1 > | multiply (const fvar< T > &c, const Eigen::Matrix< double, R1, C1 > &m) |
|  | |
| template<typename T , int R1, int C1, int R2, int C2> | |
| Eigen::Matrix< fvar< T >, R1, C2 > | multiply (const Eigen::Matrix< fvar< T >, R1, C1 > &m1, const Eigen::Matrix< fvar< T >, R2, C2 > &m2) |
|  | |
| template<typename T , int R1, int C1, int R2, int C2> | |
| Eigen::Matrix< fvar< T >, R1, C2 > | multiply (const Eigen::Matrix< fvar< T >, R1, C1 > &m1, const Eigen::Matrix< double, R2, C2 > &m2) |
|  | |
| template<typename T , int R1, int C1, int R2, int C2> | |
| Eigen::Matrix< fvar< T >, R1, C2 > | multiply (const Eigen::Matrix< double, R1, C1 > &m1, const Eigen::Matrix< fvar< T >, R2, C2 > &m2) |
|  | |
| template<typename T , int C1, int R2> | |
| fvar< T > | multiply (const Eigen::Matrix< fvar< T >, 1, C1 > &rv, const Eigen::Matrix< fvar< T >, R2, 1 > &v) |
|  | |
| template<typename T , int C1, int R2> | |
| fvar< T > | multiply (const Eigen::Matrix< fvar< T >, 1, C1 > &rv, const Eigen::Matrix< double, R2, 1 > &v) |
|  | |
| template<typename T , int C1, int R2> | |
| fvar< T > | multiply (const Eigen::Matrix< double, 1, C1 > &rv, const Eigen::Matrix< fvar< T >, R2, 1 > &v) |
|  | |
| template<typename T , int R, int C> | |
| Eigen::Matrix< fvar< T >, R, R > | multiply\_lower\_tri\_self\_transpose (const Eigen::Matrix< fvar< T >, R, C > &m) |
|  | |
| template<typename T > | |
| Eigen::Matrix< fvar< T >  , Eigen::Dynamic,   Eigen::Dynamic > | qr\_Q (const Eigen::Matrix< fvar< T >, Eigen::Dynamic, Eigen::Dynamic > &m) |
|  | |
| template<typename T > | |
| Eigen::Matrix< fvar< T >  , Eigen::Dynamic,   Eigen::Dynamic > | qr\_R (const Eigen::Matrix< fvar< T >, Eigen::Dynamic, Eigen::Dynamic > &m) |
|  | |
| template<typename T , int R1, int C1, int R2, int C2> | |
| Eigen::Matrix< fvar< T >, R1, 1 > | rows\_dot\_product (const Eigen::Matrix< fvar< T >, R1, C1 > &v1, const Eigen::Matrix< fvar< T >, R2, C2 > &v2) |
|  | |
| template<typename T , int R1, int C1, int R2, int C2> | |
| Eigen::Matrix< fvar< T >, R1, 1 > | rows\_dot\_product (const Eigen::Matrix< double, R1, C1 > &v1, const Eigen::Matrix< fvar< T >, R2, C2 > &v2) |
|  | |
| template<typename T , int R1, int C1, int R2, int C2> | |
| Eigen::Matrix< fvar< T >, R1, 1 > | rows\_dot\_product (const Eigen::Matrix< fvar< T >, R1, C1 > &v1, const Eigen::Matrix< double, R2, C2 > &v2) |
|  | |
| template<typename T , int R, int C> | |
| Eigen::Matrix< fvar< T >, R, 1 > | rows\_dot\_self (const Eigen::Matrix< fvar< T >, R, C > &x) |
|  | |
| template<typename T > | |
| Eigen::Matrix< fvar< T >  , Eigen::Dynamic, 1 > | softmax (const Eigen::Matrix< fvar< T >, Eigen::Dynamic, 1 > &alpha) |
|  | |
| template<typename T > | |
| std::vector< fvar< T > > | sort\_asc (std::vector< fvar< T > > xs) |
|  | |
| template<typename T > | |
| std::vector< fvar< T > > | sort\_desc (std::vector< fvar< T > > xs) |
|  | |
| template<typename T , int R, int C> | |
| Eigen::Matrix< fvar< T >, R, C > | sort\_asc (Eigen::Matrix< fvar< T >, R, C > xs) |
|  | |
| template<typename T , int R, int C> | |
| Eigen::Matrix< fvar< T >, R, C > | sort\_desc (Eigen::Matrix< fvar< T >, R, C > xs) |
|  | |
| template<typename T , int R, int C> | |
| fvar< T > | sum (const Eigen::Matrix< fvar< T >, R, C > &m) |
|  | |
| template<typename T , int R, int C> | |
| Eigen::Matrix< fvar< T >, R, R > | tcrossprod (const Eigen::Matrix< fvar< T >, R, C > &m) |
|  | |
| template<typename T > | |
| fvar< T > | to\_fvar (const T &x) |
|  | |
| template<typename T > | |
| fvar< T > | to\_fvar (const fvar< T > &x) |
|  | |
| matrix\_fd | to\_fvar (const stan::math::matrix\_d &m) |
|  | |
| matrix\_fd | to\_fvar (const matrix\_fd &m) |
|  | |
| matrix\_fv | to\_fvar (const matrix\_fv &m) |
|  | |
| matrix\_ffd | to\_fvar (const matrix\_ffd &m) |
|  | |
| matrix\_ffv | to\_fvar (const matrix\_ffv &m) |
|  | |
| vector\_fd | to\_fvar (const stan::math::vector\_d &v) |
|  | |
| vector\_fd | to\_fvar (const vector\_fd &v) |
|  | |
| vector\_fv | to\_fvar (const vector\_fv &v) |
|  | |
| vector\_ffd | to\_fvar (const vector\_ffd &v) |
|  | |
| vector\_ffv | to\_fvar (const vector\_ffv &v) |
|  | |
| row\_vector\_fd | to\_fvar (const stan::math::row\_vector\_d &rv) |
|  | |
| row\_vector\_fd | to\_fvar (const row\_vector\_fd &rv) |
|  | |
| row\_vector\_fv | to\_fvar (const row\_vector\_fv &rv) |
|  | |
| row\_vector\_ffd | to\_fvar (const row\_vector\_ffd &rv) |
|  | |
| row\_vector\_ffv | to\_fvar (const row\_vector\_ffv &rv) |
|  | |
| template<typename T , int R, int C> | |
| Eigen::Matrix< fvar< T >, R, C > | to\_fvar (const Eigen::Matrix< T, R, C > &val, const Eigen::Matrix< T, R, C > &deriv) |
|  | |
| template<int RD, int CD, int RA, int CA, int RB, int CB, typename T > | |
| fvar< T > | trace\_gen\_quad\_form (const Eigen::Matrix< fvar< T >, RD, CD > &D, const Eigen::Matrix< fvar< T >, RA, CA > &A, const Eigen::Matrix< fvar< T >, RB, CB > &B) |
|  | |
| template<int RA, int CA, int RB, int CB, typename T > | |
| stan::agrad::fvar< T > | trace\_quad\_form (const Eigen::Matrix< stan::agrad::fvar< T >, RA, CA > &A, const Eigen::Matrix< stan::agrad::fvar< T >, RB, CB > &B) |
|  | |
| template<typename T > | |
| fvar< T > | operator+ (const fvar< T > &x1, const fvar< T > &x2) |
|  | |
| template<typename T > | |
| fvar< T > | operator+ (const double x1, const fvar< T > &x2) |
|  | |
| template<typename T > | |
| fvar< T > | operator+ (const fvar< T > &x1, const double x2) |
|  | |
| template<typename T > | |
| fvar< T > | operator/ (const fvar< T > &x1, const fvar< T > &x2) |
|  | |
| template<typename T > | |
| fvar< T > | operator/ (const fvar< T > &x1, const double x2) |
|  | |
| template<typename T > | |
| fvar< T > | operator/ (const double x1, const fvar< T > &x2) |
|  | |
| template<typename T > | |
| bool | operator== (const fvar< T > &x, const fvar< T > &y) |
|  | |
| template<typename T > | |
| bool | operator== (const fvar< T > &x, double y) |
|  | |
| template<typename T > | |
| bool | operator== (double x, const fvar< T > &y) |
|  | |
| template<typename T > | |
| bool | operator> (const fvar< T > &x, const fvar< T > &y) |
|  | |
| template<typename T > | |
| bool | operator> (const fvar< T > &x, double y) |
|  | |
| template<typename T > | |
| bool | operator> (double x, const fvar< T > &y) |
|  | |
| template<typename T > | |
| bool | operator>= (const fvar< T > &x, const fvar< T > &y) |
|  | |
| template<typename T > | |
| bool | operator>= (const fvar< T > &x, double y) |
|  | |
| template<typename T > | |
| bool | operator>= (double x, const fvar< T > &y) |
|  | |
| template<typename T > | |
| bool | operator< (const fvar< T > &x, double y) |
|  | |
| template<typename T > | |
| bool | operator< (double x, const fvar< T > &y) |
|  | |
| template<typename T > | |
| bool | operator< (const fvar< T > &x, const fvar< T > &y) |
|  | |
| template<typename T > | |
| bool | operator<= (const fvar< T > &x, const fvar< T > &y) |
|  | |
| template<typename T > | |
| bool | operator<= (const fvar< T > &x, double y) |
|  | |
| template<typename T > | |
| bool | operator<= (double x, const fvar< T > &y) |
|  | |
| template<typename T > | |
| fvar< T > | operator\* (const fvar< T > &x1, const fvar< T > &x2) |
|  | |
| template<typename T > | |
| fvar< T > | operator\* (const double x1, const fvar< T > &x2) |
|  | |
| template<typename T > | |
| fvar< T > | operator\* (const fvar< T > &x1, const double x2) |
|  | |
| template<typename T > | |
| bool | operator!= (const fvar< T > &x, const fvar< T > &y) |
|  | |
| template<typename T > | |
| bool | operator!= (const fvar< T > &x, double y) |
|  | |
| template<typename T > | |
| bool | operator!= (double x, const fvar< T > &y) |
|  | |
| template<typename T > | |
| fvar< T > | operator- (const fvar< T > &x1, const fvar< T > &x2) |
|  | |
| template<typename T > | |
| fvar< T > | operator- (const double x1, const fvar< T > &x2) |
|  | |
| template<typename T > | |
| fvar< T > | operator- (const fvar< T > &x1, const double x2) |
|  | |
| template<typename T > | |
| fvar< T > | operator- (const fvar< T > &x) |
|  | |
| double | calculate\_chain (const double &x, const double &val) |
|  | |
| static void | set\_zero\_all\_adjoints () |
|  | Reset all adjoint values in the stack to zero. More... |
|  | |
| static void | grad (chainable \*vi) |
|  | Compute the gradient for all variables starting from the specified root variable implementation. More... |
|  | |
| template<typename T\_result , class Policy > | |
| bool | check\_pos\_definite (const char \*function, const Eigen::Matrix< var, Eigen::Dynamic, Eigen::Dynamic > &y, const char \*name, T\_result \*result, const Policy &) |
|  | |
| var | abs (const var &a) |
|  | Return the absolute value of the variable (std). More... |
|  | |
| var | acos (const var &a) |
|  | Return the principal value of the arc cosine of a variable, in radians (cmath). More... |
|  | |
| var | acosh (const stan::agrad::var &a) |
|  | The inverse hyperbolic cosine function for variables (C99). More... |
|  | |
| int | as\_bool (const agrad::var &v) |
|  | Return 1 if the argument is unequal to zero and 0 otherwise. More... |
|  | |
| var | asin (const var &a) |
|  | Return the principal value of the arc sine, in radians, of the specified variable (cmath). More... |
|  | |
| var | asinh (const stan::agrad::var &a) |
|  | The inverse hyperbolic sine function for variables (C99). More... |
|  | |
| var | atan (const var &a) |
|  | Return the principal value of the arc tangent, in radians, of the specified variable (cmath). More... |
|  | |
| var | atan2 (const var &a, const var &b) |
|  | Return the principal value of the arc tangent, in radians, of the first variable divided by the second (cmath). More... |
|  | |
| var | atan2 (const var &a, const double b) |
|  | Return the principal value of the arc tangent, in radians, of the first variable divided by the second scalar (cmath). More... |
|  | |
| var | atan2 (const double a, const var &b) |
|  | Return the principal value of the arc tangent, in radians, of the first scalar divided by the second variable (cmath). More... |
|  | |
| var | atanh (const stan::agrad::var &a) |
|  | The inverse hyperbolic tangent function for variables (C99). More... |
|  | |
| var | bessel\_first\_kind (const int &v, const var &a) |
|  | |
| var | bessel\_second\_kind (const int &v, const var &a) |
|  | |
| var | binary\_log\_loss (const int y, const stan::agrad::var &y\_hat) |
|  | The log loss function for variables (stan). More... |
|  | |
| var | cbrt (const stan::agrad::var &a) |
|  | Returns the cube root of the specified variable (C99). More... |
|  | |
| var | ceil (const var &a) |
|  | Return the ceiling of the specified variable (cmath). More... |
|  | |
| var | cos (const var &a) |
|  | Return the cosine of a radian-scaled variable (cmath). More... |
|  | |
| var | cosh (const var &a) |
|  | Return the hyperbolic cosine of the specified variable (cmath). More... |
|  | |
| var | digamma (const stan::agrad::var &a) |
|  | |
| var | erf (const stan::agrad::var &a) |
|  | The error function for variables (C99). More... |
|  | |
| var | erfc (const stan::agrad::var &a) |
|  | The complementary error function for variables (C99). More... |
|  | |
| var | exp (const var &a) |
|  | Return the exponentiation of the specified variable (cmath). More... |
|  | |
| var | exp2 (const stan::agrad::var &a) |
|  | Exponentiation base 2 function for variables (C99). More... |
|  | |
| var | expm1 (const stan::agrad::var &a) |
|  | The exponentiation of the specified variable minus 1 (C99). More... |
|  | |
| var | fabs (const var &a) |
|  | Return the absolute value of the variable (cmath). More... |
|  | |
| var | falling\_factorial (const var &a, const double &b) |
|  | |
| var | falling\_factorial (const var &a, const var &b) |
|  | |
| var | falling\_factorial (const double &a, const var &b) |
|  | |
| var | fdim (const stan::agrad::var &a, const stan::agrad::var &b) |
|  | Return the positive difference between the first variable's the value and the second's (C99). More... |
|  | |
| var | fdim (const double &a, const stan::agrad::var &b) |
|  | Return the positive difference between the first value and the value of the second variable (C99). More... |
|  | |
| var | fdim (const stan::agrad::var &a, const double &b) |
|  | Return the positive difference between the first variable's value and the second value (C99). More... |
|  | |
| var | floor (const var &a) |
|  | Return the floor of the specified variable (cmath). More... |
|  | |
| var | fma (const stan::agrad::var &a, const stan::agrad::var &b, const stan::agrad::var &c) |
|  | The fused multiply-add function for three variables (C99). More... |
|  | |
| var | fma (const stan::agrad::var &a, const stan::agrad::var &b, const double &c) |
|  | The fused multiply-add function for two variables and a value (C99). More... |
|  | |
| var | fma (const stan::agrad::var &a, const double &b, const stan::agrad::var &c) |
|  | The fused multiply-add function for a variable, value, and variable (C99). More... |
|  | |
| var | fma (const stan::agrad::var &a, const double &b, const double &c) |
|  | The fused multiply-add function for a variable and two values (C99). More... |
|  | |
| var | fma (const double &a, const stan::agrad::var &b, const double &c) |
|  | The fused multiply-add function for a value, variable, and value (C99). More... |
|  | |
| var | fma (const double &a, const double &b, const stan::agrad::var &c) |
|  | The fused multiply-add function for two values and a variable, and value (C99). More... |
|  | |
| var | fma (const double &a, const stan::agrad::var &b, const stan::agrad::var &c) |
|  | The fused multiply-add function for a value and two variables (C99). More... |
|  | |
| var | fmax (const stan::agrad::var &a, const stan::agrad::var &b) |
|  | Returns the maximum of the two variable arguments (C99). More... |
|  | |
| var | fmax (const stan::agrad::var &a, const double &b) |
|  | Returns the maximum of the variable and scalar, promoting the scalar to a variable if it is larger (C99). More... |
|  | |
| var | fmax (const double &a, const stan::agrad::var &b) |
|  | Returns the maximum of a scalar and variable, promoting the scalar to a variable if it is larger (C99). More... |
|  | |
| var | fmin (const stan::agrad::var &a, const stan::agrad::var &b) |
|  | Returns the minimum of the two variable arguments (C99). More... |
|  | |
| var | fmin (const stan::agrad::var &a, const double &b) |
|  | Returns the minimum of the variable and scalar, promoting the scalar to a variable if it is larger (C99). More... |
|  | |
| var | fmin (const double &a, const stan::agrad::var &b) |
|  | Returns the minimum of a scalar and variable, promoting the scalar to a variable if it is larger (C99). More... |
|  | |
| var | fmod (const var &a, const var &b) |
|  | Return the floating point remainder after dividing the first variable by the second (cmath). More... |
|  | |
| var | fmod (const var &a, const double b) |
|  | Return the floating point remainder after dividing the the first variable by the second scalar (cmath). More... |
|  | |
| var | fmod (const double a, const var &b) |
|  | Return the floating point remainder after dividing the first scalar by the second variable (cmath). More... |
|  | |
| var | gamma\_p (const stan::agrad::var &a, const stan::agrad::var &b) |
|  | |
| var | gamma\_p (const stan::agrad::var &a, const double &b) |
|  | |
| var | gamma\_p (const double &a, const stan::agrad::var &b) |
|  | |
| var | gamma\_q (const stan::agrad::var &a, const stan::agrad::var &b) |
|  | |
| var | gamma\_q (const stan::agrad::var &a, const double &b) |
|  | |
| var | gamma\_q (const double &a, const stan::agrad::var &b) |
|  | |
| var | hypot (const stan::agrad::var &a, const stan::agrad::var &b) |
|  | Returns the length of the hypoteneuse of a right triangle with sides of the specified lengths (C99). More... |
|  | |
| var | hypot (const stan::agrad::var &a, const double &b) |
|  | Returns the length of the hypoteneuse of a right triangle with sides of the specified lengths (C99). More... |
|  | |
| var | hypot (const double &a, const stan::agrad::var &b) |
|  | Returns the length of the hypoteneuse of a right triangle with sides of the specified lengths (C99). More... |
|  | |
| var | ibeta (const var &a, const var &b, const var &x) |
|  | The normalized incomplete beta function of a, b, and x. More... |
|  | |
| var | if\_else (bool c, const var &y\_true, const var &y\_false) |
|  | If the specified condition is true, return the first variable, otherwise return the second variable. More... |
|  | |
| var | if\_else (bool c, double y\_true, const var &y\_false) |
|  | If the specified condition is true, return a new variable constructed from the first scalar, otherwise return the second variable. More... |
|  | |
| var | if\_else (bool c, const var &y\_true, const double y\_false) |
|  | If the specified condition is true, return the first variable, otherwise return a new variable constructed from the second scalar. More... |
|  | |
| var | inv (const var &a) |
|  | More... |
|  | |
| var | inv\_cloglog (const stan::agrad::var &a) |
|  | Return the inverse complementary log-log function applied specified variable (stan). More... |
|  | |
| var | inv\_logit (const stan::agrad::var &a) |
|  | The inverse logit function for variables (stan). More... |
|  | |
| var | inv\_sqrt (const var &a) |
|  | More... |
|  | |
| var | inv\_square (const var &a) |
|  | More... |
|  | |
| int | is\_inf (const var &v) |
|  | Returns 1 if the input's value is infinite and 0 otherwise. More... |
|  | |
| int | is\_nan (const var &v) |
|  | Returns 1 if the input's value is NaN and 0 otherwise. More... |
|  | |
| bool | is\_uninitialized (var x) |
|  | Returns `true` if the specified variable is uninitialized. More... |
|  | |
| var | lgamma (const stan::agrad::var &a) |
|  | The log gamma function for variables (C99). More... |
|  | |
| var | lmgamma (int a, const stan::agrad::var &b) |
|  | |
| var | log (const var &a) |
|  | Return the natural log of the specified variable (cmath). More... |
|  | |
| var | log10 (const var &a) |
|  | Return the base 10 log of the specified variable (cmath). More... |
|  | |
| var | log1m (const stan::agrad::var &a) |
|  | The log (1 - x) function for variables. More... |
|  | |
| var | log1m\_exp (const stan::agrad::var &a) |
|  | Return the log of 1 minus the exponential of the specified variable. More... |
|  | |
| var | log1p (const stan::agrad::var &a) |
|  | The log (1 + x) function for variables (C99). More... |
|  | |
| var | log1p\_exp (const stan::agrad::var &a) |
|  | Return the log of 1 plus the exponential of the specified variable. More... |
|  | |
| var | log2 (const stan::agrad::var &a) |
|  | Returns the base 2 logarithm of the specified variable (C99). More... |
|  | |
| var | log\_diff\_exp (const stan::agrad::var &a, const stan::agrad::var &b) |
|  | Returns the log sum of exponentials. More... |
|  | |
| var | log\_diff\_exp (const stan::agrad::var &a, const double &b) |
|  | Returns the log sum of exponentials. More... |
|  | |
| var | log\_diff\_exp (const double &a, const stan::agrad::var &b) |
|  | Returns the log sum of exponentials. More... |
|  | |
| var | log\_falling\_factorial (const var &a, const double &b) |
|  | |
| var | log\_falling\_factorial (const var &a, const var &b) |
|  | |
| var | log\_falling\_factorial (const double &a, const var &b) |
|  | |
| var | log\_rising\_factorial (const var &a, const double &b) |
|  | |
| var | log\_rising\_factorial (const var &a, const var &b) |
|  | |
| var | log\_rising\_factorial (const double &a, const var &b) |
|  | |
| var | log\_sum\_exp (const stan::agrad::var &a, const stan::agrad::var &b) |
|  | Returns the log sum of exponentials. More... |
|  | |
| var | log\_sum\_exp (const stan::agrad::var &a, const double &b) |
|  | Returns the log sum of exponentials. More... |
|  | |
| var | log\_sum\_exp (const double &a, const stan::agrad::var &b) |
|  | Returns the log sum of exponentials. More... |
|  | |
| var | log\_sum\_exp (const std::vector< var > &x) |
|  | Returns the log sum of exponentials. More... |
|  | |
| var | modified\_bessel\_first\_kind (const int &v, const var &a) |
|  | |
| var | modified\_bessel\_second\_kind (const int &v, const var &a) |
|  | |
| var | multiply\_log (const var &a, const var &b) |
|  | Return the value of a\*log(b). More... |
|  | |
| var | multiply\_log (const var &a, const double b) |
|  | Return the value of a\*log(b). More... |
|  | |
| var | multiply\_log (const double a, const var &b) |
|  | Return the value of a\*log(b). More... |
|  | |
| var | owens\_t (const var &h, const var &a) |
|  | The Owen's T function of h and a. More... |
|  | |
| var | owens\_t (const var &h, const double &a) |
|  | The Owen's T function of h and a. More... |
|  | |
| var | owens\_t (const double &h, const var &a) |
|  | The Owen's T function of h and a. More... |
|  | |
| var | Phi (const stan::agrad::var &a) |
|  | The unit normal cumulative density function for variables (stan). More... |
|  | |
| var | Phi\_approx (const stan::agrad::var &a) |
|  | Approximation of the unit normal CDF for variables (stan). More... |
|  | |
| var | pow (const var &base, const var &exponent) |
|  | Return the base raised to the power of the exponent (cmath). More... |
|  | |
| var | pow (const var &base, const double exponent) |
|  | Return the base variable raised to the power of the exponent scalar (cmath). More... |
|  | |
| var | pow (const double base, const var &exponent) |
|  | Return the base scalar raised to the power of the exponent variable (cmath). More... |
|  | |
| double | primitive\_value (const agrad::var &v) |
|  | Return the primitive double value for the specified auto-diff variable. More... |
|  | |
| var | rising\_factorial (const var &a, const double &b) |
|  | |
| var | rising\_factorial (const var &a, const var &b) |
|  | |
| var | rising\_factorial (const double &a, const var &b) |
|  | |
| var | round (const stan::agrad::var &a) |
|  | Returns the rounded form of the specified variable (C99). More... |
|  | |
| var | sin (const var &a) |
|  | Return the sine of a radian-scaled variable (cmath). More... |
|  | |
| var | sinh (const var &a) |
|  | Return the hyperbolic sine of the specified variable (cmath). More... |
|  | |
| var | sqrt (const var &a) |
|  | Return the square root of the specified variable (cmath). More... |
|  | |
| var | square (const var &x) |
|  | Return the square of the input variable. More... |
|  | |
| var | step (const stan::agrad::var &a) |
|  | Return the step, or heaviside, function applied to the specified variable (stan). More... |
|  | |
| var | tan (const var &a) |
|  | Return the tangent of a radian-scaled variable (cmath). More... |
|  | |
| var | tanh (const var &a) |
|  | Return the hyperbolic tangent of the specified variable (cmath). More... |
|  | |
| var | tgamma (const stan::agrad::var &a) |
|  | Return the Gamma function applied to the specified variable (C99). More... |
|  | |
| var | trunc (const stan::agrad::var &a) |
|  | Returns the truncatation of the specified variable (C99). More... |
|  | |
| double | value\_of (const agrad::var &v) |
|  | Return the value of the specified variable. More... |
|  | |
| var | precomputed\_gradients (const double value, const std::vector< var > &vars, const std::vector< double > &gradients) |
|  | This function is provided for Stan users that want to compute gradients without using Stan's auto-diff. More... |
|  | |
| matrix\_v | crossprod (const matrix\_v &M) |
|  | Returns the result of pre-multiplying a matrix by its own transpose. More... |
|  | |
| template<int R, int C> | |
| var | determinant (const Eigen::Matrix< var, R, C > &m) |
|  | |
| double | divide (double x, double y) |
|  | Return the division of the first scalar by the second scalar. More... |
|  | |
| template<typename T1 , typename T2 > | |
| var | divide (const T1 &v, const T2 &c) |
|  | |
| template<typename T1 , typename T2 , int R, int C> | |
| Eigen::Matrix< var, R, C > | divide (const Eigen::Matrix< T1, R, C > &v, const T2 &c) |
|  | Return the division of the specified column vector by the specified scalar. More... |
|  | |
| template<typename T1 , int R1, int C1, typename T2 , int R2, int C2> | |
| boost::enable\_if\_c  < boost::is\_same< T1, var >  ::value\|\|boost::is\_same< T2,   var >::value, var >::type | dot\_product (const Eigen::Matrix< T1, R1, C1 > &v1, const Eigen::Matrix< T2, R2, C2 > &v2) |
|  | Returns the dot product. More... |
|  | |
| template<typename T1 , typename T2 > | |
| boost::enable\_if\_c  < boost::is\_same< T1, var >  ::value\|\|boost::is\_same< T2,   var >::value, var >::type | dot\_product (const T1 \*v1, const T2 \*v2, size\_t length) |
|  | Returns the dot product. More... |
|  | |
| template<typename T1 , typename T2 > | |
| boost::enable\_if\_c  < boost::is\_same< T1, var >  ::value\|\|boost::is\_same< T2,   var >::value, var >::type | dot\_product (const std::vector< T1 > &v1, const std::vector< T2 > &v2) |
|  | Returns the dot product. More... |
|  | |
| template<typename T1 , int R1, int C1, typename T2 , int R2, int C2> | |
| boost::enable\_if\_c  < boost::is\_same< T1, var >  ::value\|\|boost::is\_same< T2,   var >::value, Eigen::Matrix  < var, 1, C1 > >::type | columns\_dot\_product (const Eigen::Matrix< T1, R1, C1 > &v1, const Eigen::Matrix< T2, R2, C2 > &v2) |
|  | |
| template<typename T1 , int R1, int C1, typename T2 , int R2, int C2> | |
| boost::enable\_if\_c  < boost::is\_same< T1, var >  ::value\|\|boost::is\_same< T2,   var >::value, Eigen::Matrix  < var, R1, 1 > >::type | rows\_dot\_product (const Eigen::Matrix< T1, R1, C1 > &v1, const Eigen::Matrix< T2, R2, C2 > &v2) |
|  | |
| template<int R, int C> | |
| var | dot\_self (const Eigen::Matrix< var, R, C > &v) |
|  | Returns the dot product of a vector with itself. More... |
|  | |
| template<int R, int C> | |
| Eigen::Matrix< var, 1, C > | columns\_dot\_self (const Eigen::Matrix< var, R, C > &x) |
|  | Returns the dot product of each column of a matrix with itself. More... |
|  | |
| void | grad (var &v, Eigen::Matrix< var, Eigen::Dynamic, 1 > &x, Eigen::VectorXd &g) |
|  | Propagate chain rule to calculate gradients starting from the specified variable. More... |
|  | |
| void | initialize\_variable (var &variable, const var &value) |
|  | Initialize variable to value. More... |
|  | |
| template<int R, int C> | |
| void | initialize\_variable (Eigen::Matrix< var, R, C > &matrix, const var &value) |
|  | Initialize every cell in the matrix to the specified value. More... |
|  | |
| template<typename T > | |
| void | initialize\_variable (std::vector< T > &variables, const var &value) |
|  | Initialize the variables in the standard vector recursively. More... |
|  | |
| template<int R, int C> | |
| var | log\_determinant (const Eigen::Matrix< var, R, C > &m) |
|  | |
| template<int R, int C> | |
| var | log\_determinant\_ldlt (stan::math::LDLT\_factor< var, R, C > &A) |
|  | |
| template<int R, int C> | |
| var | log\_determinant\_spd (const Eigen::Matrix< var, R, C > &m) |
|  | |
| Eigen::Matrix< var,   Eigen::Dynamic, 1 > | log\_softmax (const Eigen::Matrix< var, Eigen::Dynamic, 1 > &alpha) |
|  | Return the softmax of the specified Eigen vector. More... |
|  | |
| template<int R, int C> | |
| var | log\_sum\_exp (const Eigen::Matrix< var, R, C > &x) |
|  | Returns the log sum of exponentials. More... |
|  | |
| template<int R1, int C1, int R2, int C2> | |
| Eigen::Matrix< var, R1, C2 > | mdivide\_left (const Eigen::Matrix< var, R1, C1 > &A, const Eigen::Matrix< var, R2, C2 > &b) |
|  | |
| template<int R1, int C1, int R2, int C2> | |
| Eigen::Matrix< var, R1, C2 > | mdivide\_left (const Eigen::Matrix< var, R1, C1 > &A, const Eigen::Matrix< double, R2, C2 > &b) |
|  | |
| template<int R1, int C1, int R2, int C2> | |
| Eigen::Matrix< var, R1, C2 > | mdivide\_left (const Eigen::Matrix< double, R1, C1 > &A, const Eigen::Matrix< var, R2, C2 > &b) |
|  | |
| template<int R1, int C1, int R2, int C2> | |
| Eigen::Matrix< var, R1, C2 > | mdivide\_left\_ldlt (const stan::math::LDLT\_factor< var, R1, C1 > &A, const Eigen::Matrix< var, R2, C2 > &b) |
|  | Returns the solution of the system Ax=b given an LDLT\_factor of A. More... |
|  | |
| template<int R1, int C1, int R2, int C2> | |
| Eigen::Matrix< var, R1, C2 > | mdivide\_left\_ldlt (const stan::math::LDLT\_factor< var, R1, C1 > &A, const Eigen::Matrix< double, R2, C2 > &b) |
|  | Returns the solution of the system Ax=b given an LDLT\_factor of A. More... |
|  | |
| template<int R1, int C1, int R2, int C2> | |
| Eigen::Matrix< var, R1, C2 > | mdivide\_left\_ldlt (const stan::math::LDLT\_factor< double, R1, C1 > &A, const Eigen::Matrix< var, R2, C2 > &b) |
|  | Returns the solution of the system Ax=b given an LDLT\_factor of A. More... |
|  | |
| template<int R1, int C1, int R2, int C2> | |
| Eigen::Matrix< var, R1, C2 > | mdivide\_left\_spd (const Eigen::Matrix< var, R1, C1 > &A, const Eigen::Matrix< var, R2, C2 > &b) |
|  | |
| template<int R1, int C1, int R2, int C2> | |
| Eigen::Matrix< var, R1, C2 > | mdivide\_left\_spd (const Eigen::Matrix< var, R1, C1 > &A, const Eigen::Matrix< double, R2, C2 > &b) |
|  | |
| template<int R1, int C1, int R2, int C2> | |
| Eigen::Matrix< var, R1, C2 > | mdivide\_left\_spd (const Eigen::Matrix< double, R1, C1 > &A, const Eigen::Matrix< var, R2, C2 > &b) |
|  | |
| template<int TriView, int R1, int C1, int R2, int C2> | |
| Eigen::Matrix< var, R1, C2 > | mdivide\_left\_tri (const Eigen::Matrix< var, R1, C1 > &A, const Eigen::Matrix< var, R2, C2 > &b) |
|  | |
| template<int TriView, int R1, int C1, int R2, int C2> | |
| Eigen::Matrix< var, R1, C2 > | mdivide\_left\_tri (const Eigen::Matrix< double, R1, C1 > &A, const Eigen::Matrix< var, R2, C2 > &b) |
|  | |
| template<int TriView, int R1, int C1, int R2, int C2> | |
| Eigen::Matrix< var, R1, C2 > | mdivide\_left\_tri (const Eigen::Matrix< var, R1, C1 > &A, const Eigen::Matrix< double, R2, C2 > &b) |
|  | |
| template<typename T1 , typename T2 > | |
| boost::enable\_if\_c  < (boost::is\_scalar< T1 >  ::value\|\|boost::is\_same< T1,   var >::value)&&(boost::is\_scalar  < T2 >::value\|\|boost::is\_same  < T2, var >::value), typename   boost::math::tools::promote\_args  < T1, T2 >::type >::type | multiply (const T1 &v, const T2 &c) |
|  | Return the product of two scalars. More... |
|  | |
| template<typename T1 , typename T2 , int R2, int C2> | |
| Eigen::Matrix< var, R2, C2 > | multiply (const T1 &c, const Eigen::Matrix< T2, R2, C2 > &m) |
|  | Return the product of scalar and matrix. More... |
|  | |
| template<typename T1 , int R1, int C1, typename T2 > | |
| Eigen::Matrix< var, R1, C1 > | multiply (const Eigen::Matrix< T1, R1, C1 > &m, const T2 &c) |
|  | Return the product of scalar and matrix. More... |
|  | |
| template<typename T1 , int R1, int C1, typename T2 , int R2, int C2> | |
| boost::enable\_if\_c  < boost::is\_same< T1, var >  ::value\|\|boost::is\_same< T2,   var >::value, Eigen::Matrix  < var, R1, C2 > >::type | multiply (const Eigen::Matrix< T1, R1, C1 > &m1, const Eigen::Matrix< T2, R2, C2 > &m2) |
|  | Return the product of the specified matrices. More... |
|  | |
| template<typename T1 , int C1, typename T2 , int R2> | |
| boost::enable\_if\_c  < boost::is\_same< T1, var >  ::value\|\|boost::is\_same< T2,   var >::value, var >::type | multiply (const Eigen::Matrix< T1, 1, C1 > &rv, const Eigen::Matrix< T2, R2, 1 > &v) |
|  | Return the scalar product of the specified row vector and specified column vector. More... |
|  | |
| matrix\_v | multiply\_lower\_tri\_self\_transpose (const matrix\_v &L) |
|  | |
| template<typename TA , int RA, int CA, typename TB , int RB, int CB> | |
| boost::enable\_if\_c  < boost::is\_same< TA, var >  ::value\|\|boost::is\_same< TB,   var >::value, Eigen::Matrix  < var, CB, CB > >::type | quad\_form (const Eigen::Matrix< TA, RA, CA > &A, const Eigen::Matrix< TB, RB, CB > &B) |
|  | |
| template<typename TA , int RA, int CA, typename TB , int RB> | |
| boost::enable\_if\_c  < boost::is\_same< TA, var >  ::value\|\|boost::is\_same< TB,   var >::value, var >::type | quad\_form (const Eigen::Matrix< TA, RA, CA > &A, const Eigen::Matrix< TB, RB, 1 > &B) |
|  | |
| template<typename TA , int RA, int CA, typename TB , int RB, int CB> | |
| boost::enable\_if\_c  < boost::is\_same< TA, var >  ::value\|\|boost::is\_same< TB,   var >::value, Eigen::Matrix  < var, CB, CB > >::type | quad\_form\_sym (const Eigen::Matrix< TA, RA, CA > &A, const Eigen::Matrix< TB, RB, CB > &B) |
|  | |
| template<typename TA , int RA, int CA, typename TB , int RB> | |
| boost::enable\_if\_c  < boost::is\_same< TA, var >  ::value\|\|boost::is\_same< TB,   var >::value, var >::type | quad\_form\_sym (const Eigen::Matrix< TA, RA, CA > &A, const Eigen::Matrix< TB, RB, 1 > &B) |
|  | |
| var | sd (const std::vector< var > &v) |
|  | Return the sample standard deviation of the specified standard vector. More... |
|  | |
| template<int R, int C> | |
| var | sd (const Eigen::Matrix< var, R, C > &m) |
|  | |
| Eigen::Matrix< var,   Eigen::Dynamic, 1 > | softmax (const Eigen::Matrix< var, Eigen::Dynamic, 1 > &alpha) |
|  | Return the softmax of the specified Eigen vector. More... |
|  | |
| std::vector< var > | sort\_asc (std::vector< var > xs) |
|  | Return the specified standard vector in ascending order with gradients kept. More... |
|  | |
| std::vector< var > | sort\_desc (std::vector< var > xs) |
|  | Return the specified standard vector in descending order with gradients kept. More... |
|  | |
| template<int R, int C> | |
| Eigen::Matrix< var, R, C > | sort\_asc (Eigen::Matrix< var, R, C > xs) |
|  | Return the specified eigen vector in ascending order with gradients kept. More... |
|  | |
| template<int R, int C> | |
| Eigen::Matrix< var, R, C > | sort\_desc (Eigen::Matrix< var, R, C > xs) |
|  | Return the specified eigen vector in descending order with gradients kept. More... |
|  | |
| template<int R1, int C1, int R2, int C2> | |
| var | squared\_distance (const Eigen::Matrix< var, R1, C1 > &v1, const Eigen::Matrix< var, R2, C2 > &v2) |
|  | |
| template<int R1, int C1, int R2, int C2> | |
| var | squared\_distance (const Eigen::Matrix< var, R1, C1 > &v1, const Eigen::Matrix< double, R2, C2 > &v2) |
|  | |
| template<int R1, int C1, int R2, int C2> | |
| var | squared\_distance (const Eigen::Matrix< double, R1, C1 > &v1, const Eigen::Matrix< var, R2, C2 > &v2) |
|  | |
| void | stan\_print (std::ostream \*o, const var &x) |
|  | |
| template<int R, int C> | |
| var | sum (const Eigen::Matrix< var, R, C > &m) |
|  | Returns the sum of the coefficients of the specified matrix, column vector or row vector. More... |
|  | |
| matrix\_v | tcrossprod (const matrix\_v &M) |
|  | Returns the result of post-multiplying a matrix by its own transpose. More... |
|  | |
| var | to\_var (const double &x) |
|  | Converts argument to an automatic differentiation variable. More... |
|  | |
| var | to\_var (const var &x) |
|  | Converts argument to an automatic differentiation variable. More... |
|  | |
| matrix\_v | to\_var (const stan::math::matrix\_d &m) |
|  | Converts argument to an automatic differentiation variable. More... |
|  | |
| matrix\_v | to\_var (const matrix\_v &m) |
|  | Converts argument to an automatic differentiation variable. More... |
|  | |
| vector\_v | to\_var (const stan::math::vector\_d &v) |
|  | Converts argument to an automatic differentiation variable. More... |
|  | |
| vector\_v | to\_var (const vector\_v &v) |
|  | Converts argument to an automatic differentiation variable. More... |
|  | |
| row\_vector\_v | to\_var (const stan::math::row\_vector\_d &rv) |
|  | Converts argument to an automatic differentiation variable. More... |
|  | |
| row\_vector\_v | to\_var (const row\_vector\_v &rv) |
|  | Converts argument to an automatic differentiation variable. More... |
|  | |
| template<typename T1 , int R1, int C1, typename T2 , int R2, int C2, typename T3 , int R3, int C3> | |
| boost::enable\_if\_c  < boost::is\_same< T1, var >  ::value\|\|boost::is\_same< T2,   var >::value\|\|boost::is\_same  < T3, var >::value, var >  ::type | trace\_gen\_inv\_quad\_form\_ldlt (const Eigen::Matrix< T1, R1, C1 > &D, const stan::math::LDLT\_factor< T2, R2, C2 > &A, const Eigen::Matrix< T3, R3, C3 > &B) |
|  | Compute the trace of an inverse quadratic form. More... |
|  | |
| template<typename TD , int RD, int CD, typename TA , int RA, int CA, typename TB , int RB, int CB> | |
| boost::enable\_if\_c  < boost::is\_same< TD, var >  ::value\|\|boost::is\_same< TA,   var >::value\|\|boost::is\_same  < TB, var >::value, var >  ::type | trace\_gen\_quad\_form (const Eigen::Matrix< TD, RD, CD > &D, const Eigen::Matrix< TA, RA, CA > &A, const Eigen::Matrix< TB, RB, CB > &B) |
|  | |
| template<typename T2 , int R2, int C2, typename T3 , int R3, int C3> | |
| boost::enable\_if\_c  < boost::is\_same< T2, var >  ::value\|\|boost::is\_same< T3,   var >::value, var >::type | trace\_inv\_quad\_form\_ldlt (const stan::math::LDLT\_factor< T2, R2, C2 > &A, const Eigen::Matrix< T3, R3, C3 > &B) |
|  | Compute the trace of an inverse quadratic form. More... |
|  | |
| template<typename TA , int RA, int CA, typename TB , int RB, int CB> | |
| boost::enable\_if\_c  < boost::is\_same< TA, var >  ::value\|\|boost::is\_same< TB,   var >::value, var >::type | trace\_quad\_form (const Eigen::Matrix< TA, RA, CA > &A, const Eigen::Matrix< TB, RB, CB > &B) |
|  | |
| template<int R, int C> | |
| const Eigen::Matrix< double, R,   C > & | value\_of (const Eigen::Matrix< double, R, C > &M) |
|  | Convert a matrix to a matrix of doubles. More... |
|  | |
| template<int R, int C> | |
| Eigen::Matrix< double, R, C > | value\_of (const Eigen::Matrix< var, R, C > &M) |
|  | Convert a matrix to a matrix of doubles. More... |
|  | |
| var | variance (const std::vector< var > &v) |
|  | Return the sample variance of the specified standard vector. More... |
|  | |
| template<int R, int C> | |
| var | variance (const Eigen::Matrix< var, R, C > &m) |
|  | |
| var | operator+ (const var &a, const var &b) |
|  | Addition operator for variables (C++). More... |
|  | |
| var | operator+ (const var &a, const double b) |
|  | Addition operator for variable and scalar (C++). More... |
|  | |
| var | operator+ (const double a, const var &b) |
|  | Addition operator for scalar and variable (C++). More... |
|  | |
| var | operator/ (const var &a, const var &b) |
|  | Division operator for two variables (C++). More... |
|  | |
| var | operator/ (const var &a, const double b) |
|  | Division operator for dividing a variable by a scalar (C++). More... |
|  | |
| var | operator/ (const double a, const var &b) |
|  | Division operator for dividing a scalar by a variable (C++). More... |
|  | |
| bool | operator== (const var &a, const var &b) |
|  | Equality operator comparing two variables' values (C++). More... |
|  | |
| bool | operator== (const var &a, const double b) |
|  | Equality operator comparing a variable's value and a double (C++). More... |
|  | |
| bool | operator== (const double a, const var &b) |
|  | Equality operator comparing a scalar and a variable's value (C++). More... |
|  | |
| bool | operator> (const var &a, const var &b) |
|  | Greater than operator comparing variables' values (C++). More... |
|  | |
| bool | operator> (const var &a, const double b) |
|  | Greater than operator comparing variable's value and double (C++). More... |
|  | |
| bool | operator> (const double a, const var &b) |
|  | Greater than operator comparing a double and a variable's value (C++). More... |
|  | |
| bool | operator>= (const var &a, const var &b) |
|  | Greater than or equal operator comparing two variables' values (C++). More... |
|  | |
| bool | operator>= (const var &a, const double b) |
|  | Greater than or equal operator comparing variable's value and double (C++). More... |
|  | |
| bool | operator>= (const double a, const var &b) |
|  | Greater than or equal operator comparing double and variable's value (C++). More... |
|  | |
| bool | operator< (const var &a, const var &b) |
|  | Less than operator comparing variables' values (C++). More... |
|  | |
| bool | operator< (const var &a, const double b) |
|  | Less than operator comparing variable's value and a double (C++). More... |
|  | |
| bool | operator< (const double a, const var &b) |
|  | Less than operator comparing a double and variable's value (C++). More... |
|  | |
| bool | operator<= (const var &a, const var &b) |
|  | Less than or equal operator comparing two variables' values (C++). More... |
|  | |
| bool | operator<= (const var &a, const double b) |
|  | Less than or equal operator comparing a variable's value and a scalar (C++). More... |
|  | |
| bool | operator<= (const double a, const var &b) |
|  | Less than or equal operator comparing a double and variable's value (C++). More... |
|  | |
| var | operator\* (const var &a, const var &b) |
|  | Multiplication operator for two variables (C++). More... |
|  | |
| var | operator\* (const var &a, const double b) |
|  | Multiplication operator for a variable and a scalar (C++). More... |
|  | |
| var | operator\* (const double a, const var &b) |
|  | Multiplication operator for a scalar and a variable (C++). More... |
|  | |
| bool | operator!= (const var &a, const var &b) |
|  | Inequality operator comparing two variables' values (C++). More... |
|  | |
| bool | operator!= (const var &a, const double b) |
|  | Inequality operator comparing a variable's value and a double (C++). More... |
|  | |
| bool | operator!= (const double a, const var &b) |
|  | Inequality operator comparing a double and a variable's value (C++). More... |
|  | |
| var | operator- (const var &a, const var &b) |
|  | Subtraction operator for variables (C++). More... |
|  | |
| var | operator- (const var &a, const double b) |
|  | Subtraction operator for variable and scalar (C++). More... |
|  | |
| var | operator- (const double a, const var &b) |
|  | Subtraction operator for scalar and variable (C++). More... |
|  | |
| var & | operator-- (var &a) |
|  | Prefix decrement operator for variables (C++). More... |
|  | |
| var | operator-- (var &a, int) |
|  | Postfix decrement operator for variables (C++). More... |
|  | |
| var & | operator++ (var &a) |
|  | Prefix increment operator for variables (C++). More... |
|  | |
| var | operator++ (var &a, int) |
|  | Postfix increment operator for variables (C++). More... |
|  | |
| var | operator- (const var &a) |
|  | Unary negation operator for variables (C++). More... |
|  | |
| bool | operator! (const var &a) |
|  | Prefix logical negation for the value of variables (C++). More... |
|  | |
| var | operator+ (const var &a) |
|  | Unary plus operator for variables (C++). More... |
|  | |
| void | print\_stack (std::ostream &o) |
|  | Prints the auto-dif variable stack. More... |
|  | |
| static void | grad (chainable \*vi) |
|  | |
| static bool | empty\_nested () |
|  | Return true if there is no nested autodiff being executed. More... |
|  | |
| static void | recover\_memory () |
|  | Recover memory used for all variables for reuse. More... |
|  | |
| static void | recover\_memory\_nested () |
|  | Recover only the memory used for the top nested call. More... |
|  | |
| static void | start\_nested () |
|  | Record the current position so that `recover_memory_nested()` can find it. More... |
|  | |
| static size\_t | nested\_size () |
|  | |

|  |  |
| --- | --- |
| Variables | |
| std::vector< chainable \* > | var\_stack\_ |
|  | |
| std::vector< chainable \* > | var\_nochain\_stack\_ |
|  | |
| std::vector< chainable\_alloc \* > | var\_alloc\_stack\_ |
|  | |
| memory::stack\_alloc | memalloc\_ |
|  | |
| std::vector< size\_t > | nested\_var\_stack\_sizes\_ |
|  | |
| std::vector< size\_t > | nested\_var\_nochain\_stack\_sizes\_ |
|  | |
| std::vector< size\_t > | nested\_var\_alloc\_stack\_starts\_ |
|  | |

## Detailed Description

Function gradients via reverse-mode automatic differentiation.

## Typedef Documentation

typedef Eigen::Matrix<fvar<double>,Eigen::Dynamic,Eigen::Dynamic> stan::agrad::matrix\_fd

Definition at line 18 of file typedefs.hpp.

typedef Eigen::Matrix<fvar<fvar<double> >,Eigen::Dynamic,Eigen::Dynamic> stan::agrad::matrix\_ffd

Definition at line 26 of file typedefs.hpp.

typedef Eigen::Matrix<fvar<fvar<var> >,Eigen::Dynamic,Eigen::Dynamic> stan::agrad::matrix\_ffv

Definition at line 30 of file typedefs.hpp.

typedef Eigen::Matrix<fvar<var>,Eigen::Dynamic,Eigen::Dynamic> stan::agrad::matrix\_fv

Definition at line 22 of file typedefs.hpp.

typedef Eigen::Matrix<var,Eigen::Dynamic,Eigen::Dynamic> stan::agrad::matrix\_v

The type of a matrix holding `stan::agrad::var` values.

Definition at line 21 of file typedefs.hpp.

typedef Eigen::Matrix<fvar<double>,1,Eigen::Dynamic> stan::agrad::row\_vector\_fd

Definition at line 50 of file typedefs.hpp.

typedef Eigen::Matrix<fvar<fvar<double> >,1,Eigen::Dynamic> stan::agrad::row\_vector\_ffd

Definition at line 58 of file typedefs.hpp.

typedef Eigen::Matrix<fvar<fvar<var> >,1,Eigen::Dynamic> stan::agrad::row\_vector\_ffv

Definition at line 62 of file typedefs.hpp.

typedef Eigen::Matrix<fvar<var>,1,Eigen::Dynamic> stan::agrad::row\_vector\_fv

Definition at line 54 of file typedefs.hpp.

typedef Eigen::Matrix<var,1,Eigen::Dynamic> stan::agrad::row\_vector\_v

The type of a row vector holding `stan::agrad::var` values.

Definition at line 37 of file typedefs.hpp.

typedef Eigen::Matrix< double, Eigen::Dynamic, Eigen::Dynamic >::Index stan::agrad::size\_type

Definition at line 14 of file typedefs.hpp.

typedef Eigen::Matrix<fvar<double>,Eigen::Dynamic,1> stan::agrad::vector\_fd

Definition at line 34 of file typedefs.hpp.

typedef Eigen::Matrix<fvar<fvar<double> >,Eigen::Dynamic,1> stan::agrad::vector\_ffd

Definition at line 42 of file typedefs.hpp.

typedef Eigen::Matrix<fvar<fvar<var> >,Eigen::Dynamic,1> stan::agrad::vector\_ffv

Definition at line 46 of file typedefs.hpp.

typedef Eigen::Matrix<fvar<var>,Eigen::Dynamic,1> stan::agrad::vector\_fv

Definition at line 38 of file typedefs.hpp.

typedef Eigen::Matrix<var,Eigen::Dynamic,1> stan::agrad::vector\_v

The type of a (column) vector holding `stan::agrad::var` values.

Definition at line 29 of file typedefs.hpp.

## Function Documentation

template<typename T >

|  |  |  |  |  |  |
| --- | --- | --- | --- | --- | --- |
| fvar<T> stan::agrad::abs | ( | const fvar< T > & | *x* | ) |  |

inline

Definition at line 19 of file abs.hpp.

|  |  |  |  |  |  |
| --- | --- | --- | --- | --- | --- |
| var stan::agrad::abs | ( | const var & | *a* | ) |  |

inline

Return the absolute value of the variable (std).

Delegates to `fabs()` (see for doc).

Parameters
:   |  |  |
    | --- | --- |
    | a | Variable input. |

Returns
:   Absolute value of variable.

Definition at line 35 of file abs.hpp.

template<typename T >

|  |  |  |  |  |  |
| --- | --- | --- | --- | --- | --- |
| fvar<T> stan::agrad::acos | ( | const fvar< T > & | *x* | ) |  |

inline

Definition at line 16 of file acos.hpp.

|  |  |  |  |  |  |
| --- | --- | --- | --- | --- | --- |
| var stan::agrad::acos | ( | const var & | *a* | ) |  |

inline

Return the principal value of the arc cosine of a variable, in radians (cmath).

The derivative is defined by

.

Parameters
:   |  |  |
    | --- | --- |
    | a | Variable in range [-1,1]. |

Returns
:   Arc cosine of variable, in radians.

Definition at line 60 of file acos.hpp.

template<typename T >

|  |  |  |  |  |  |
| --- | --- | --- | --- | --- | --- |
| fvar<T> stan::agrad::acosh | ( | const fvar< T > & | *x* | ) |  |

inline

Definition at line 17 of file acosh.hpp.

|  |  |  |  |  |  |
| --- | --- | --- | --- | --- | --- |
| var stan::agrad::acosh | ( | const stan::agrad::var & | *a* | ) |  |

inline

The inverse hyperbolic cosine function for variables (C99).

For non-variable function, see boost::math::acosh().

The derivative is defined by

.

Parameters
:   |  |  |
    | --- | --- |
    | a | The variable. |

Returns
:   Inverse hyperbolic cosine of the variable.

Definition at line 67 of file acosh.hpp.

|  |  |  |  |  |  |
| --- | --- | --- | --- | --- | --- |
| int stan::agrad::as\_bool | ( | const agrad::var & | *v* | ) |  |

inline

Return 1 if the argument is unequal to zero and 0 otherwise.

Parameters
:   |  |  |
    | --- | --- |
    | v | Value. |

Returns
:   1 if argument is equal to zero (or NaN) and 0 otherwise.

Definition at line 15 of file as\_bool.hpp.

template<typename T >

|  |  |  |  |  |  |
| --- | --- | --- | --- | --- | --- |
| fvar<T> stan::agrad::asin | ( | const fvar< T > & | *x* | ) |  |

inline

Definition at line 16 of file asin.hpp.

|  |  |  |  |  |  |
| --- | --- | --- | --- | --- | --- |
| var stan::agrad::asin | ( | const var & | *a* | ) |  |

inline

Return the principal value of the arc sine, in radians, of the specified variable (cmath).

The derivative is defined by

.

Parameters
:   |  |  |
    | --- | --- |
    | a | Variable in range [-1,1]. |

Returns
:   Arc sine of variable, in radians.

Definition at line 60 of file asin.hpp.

template<typename T >

|  |  |  |  |  |  |
| --- | --- | --- | --- | --- | --- |
| fvar<T> stan::agrad::asinh | ( | const fvar< T > & | *x* | ) |  |

inline

Definition at line 16 of file asinh.hpp.

|  |  |  |  |  |  |
| --- | --- | --- | --- | --- | --- |
| var stan::agrad::asinh | ( | const stan::agrad::var & | *a* | ) |  |

inline

The inverse hyperbolic sine function for variables (C99).

For non-variable function, see boost::math::asinh().

The derivative is defined by

.

Parameters
:   |  |  |
    | --- | --- |
    | a | The variable. |

Returns
:   Inverse hyperbolic sine of the variable.

Definition at line 65 of file asinh.hpp.

template<typename T >

|  |  |  |  |  |  |
| --- | --- | --- | --- | --- | --- |
| fvar<T> stan::agrad::atan | ( | const fvar< T > & | *x* | ) |  |

inline

Definition at line 16 of file atan.hpp.

|  |  |  |  |  |  |
| --- | --- | --- | --- | --- | --- |
| var stan::agrad::atan | ( | const var & | *a* | ) |  |

inline

Return the principal value of the arc tangent, in radians, of the specified variable (cmath).

The derivative is defined by

.

Parameters
:   |  |  |
    | --- | --- |
    | a | Variable in range [-1,1]. |

Returns
:   Arc tangent of variable, in radians.

Definition at line 56 of file atan.hpp.

template<typename T >

|  |  |  |  |
| --- | --- | --- | --- |
| fvar<T> stan::agrad::atan2 | ( | const fvar< T > & | *x1*, |
|  |  | const fvar< T > & | *x2* |
|  | ) |  |  |

inline

Definition at line 16 of file atan2.hpp.

template<typename T >

|  |  |  |  |
| --- | --- | --- | --- |
| fvar<T> stan::agrad::atan2 | ( | const double | *x1*, |
|  |  | const fvar< T > & | *x2* |
|  | ) |  |  |

inline

Definition at line 27 of file atan2.hpp.

template<typename T >

|  |  |  |  |
| --- | --- | --- | --- |
| fvar<T> stan::agrad::atan2 | ( | const fvar< T > & | *x1*, |
|  |  | const double | *x2* |
|  | ) |  |  |

inline

Definition at line 37 of file atan2.hpp.

|  |  |  |  |
| --- | --- | --- | --- |
| var stan::agrad::atan2 | ( | const var & | *a*, |
|  |  | const var & | *b* |
|  | ) |  |  |

inline

Return the principal value of the arc tangent, in radians, of the first variable divided by the second (cmath).

The partial derivatives are defined by

, and

.

Parameters
:   |  |  |
    | --- | --- |
    | a | Numerator variable. |
    | b | Denominator variable. |

Returns
:   The arc tangent of the fraction, in radians.

Definition at line 64 of file atan2.hpp.

|  |  |  |  |
| --- | --- | --- | --- |
| var stan::agrad::atan2 | ( | const var & | *a*, |
|  |  | const double | *b* |
|  | ) |  |  |

inline

Return the principal value of the arc tangent, in radians, of the first variable divided by the second scalar (cmath).

The derivative with respect to the variable is

.

Parameters
:   |  |  |
    | --- | --- |
    | a | Numerator variable. |
    | b | Denominator scalar. |

Returns
:   The arc tangent of the fraction, in radians.

Definition at line 80 of file atan2.hpp.

|  |  |  |  |
| --- | --- | --- | --- |
| var stan::agrad::atan2 | ( | const double | *a*, |
|  |  | const var & | *b* |
|  | ) |  |  |

inline

Return the principal value of the arc tangent, in radians, of the first scalar divided by the second variable (cmath).

The derivative with respect to the variable is

.

Parameters
:   |  |  |
    | --- | --- |
    | a | Numerator scalar. |
    | b | Denominator variable. |

Returns
:   The arc tangent of the fraction, in radians.

Definition at line 121 of file atan2.hpp.

template<typename T >

|  |  |  |  |  |  |
| --- | --- | --- | --- | --- | --- |
| fvar<T> stan::agrad::atanh | ( | const fvar< T > & | *x* | ) |  |

inline

Definition at line 16 of file atanh.hpp.

|  |  |  |  |  |  |
| --- | --- | --- | --- | --- | --- |
| var stan::agrad::atanh | ( | const stan::agrad::var & | *a* | ) |  |

inline

The inverse hyperbolic tangent function for variables (C99).

For non-variable function, see boost::math::atanh().

The derivative is defined by

.

Parameters
:   |  |  |
    | --- | --- |
    | a | The variable. |

Returns
:   Inverse hyperbolic tangent of the variable.

Definition at line 65 of file atanh.hpp.

template<typename T >

|  |  |  |  |
| --- | --- | --- | --- |
| fvar<T> stan::agrad::bessel\_first\_kind | ( | int | *v*, |
|  |  | const fvar< T > & | *z* |
|  | ) |  |  |

inline

Definition at line 15 of file bessel\_first\_kind.hpp.

|  |  |  |  |
| --- | --- | --- | --- |
| var stan::agrad::bessel\_first\_kind | ( | const int & | *v*, |
|  |  | const var & | *a* |
|  | ) |  |  |

inline

Definition at line 27 of file bessel\_first\_kind.hpp.

template<typename T >

|  |  |  |  |
| --- | --- | --- | --- |
| fvar<T> stan::agrad::bessel\_second\_kind | ( | int | *v*, |
|  |  | const fvar< T > & | *z* |
|  | ) |  |  |

inline

Definition at line 15 of file bessel\_second\_kind.hpp.

|  |  |  |  |
| --- | --- | --- | --- |
| var stan::agrad::bessel\_second\_kind | ( | const int & | *v*, |
|  |  | const var & | *a* |
|  | ) |  |  |

inline

Definition at line 27 of file bessel\_second\_kind.hpp.

template<typename T >

|  |  |  |  |
| --- | --- | --- | --- |
| fvar<T> stan::agrad::binary\_log\_loss | ( | const int | *y*, |
|  |  | const fvar< T > & | *y\_hat* |
|  | ) |  |  |

inline

Definition at line 15 of file binary\_log\_loss.hpp.

|  |  |  |  |
| --- | --- | --- | --- |
| var stan::agrad::binary\_log\_loss | ( | const int | *y*, |
|  |  | const stan::agrad::var & | *y\_hat* |
|  | ) |  |  |

inline

The log loss function for variables (stan).

See stan::math::binary\_log\_loss() for the double-based version.

The derivative with respect to the variable  is

, and

.

Parameters
:   |  |  |
    | --- | --- |
    | y | Reference value. |
    | y\_hat | Response variable. |

Returns
:   Log loss of response versus reference value.

Definition at line 69 of file binary\_log\_loss.hpp.

template<typename T >

|  |  |  |  |
| --- | --- | --- | --- |
| fvar<T> stan::agrad::binomial\_coefficient\_log | ( | const fvar< T > & | *x1*, |
|  |  | const fvar< T > & | *x2* |
|  | ) |  |  |

inline

Definition at line 16 of file binomial\_coefficient\_log.hpp.

template<typename T >

|  |  |  |  |
| --- | --- | --- | --- |
| fvar<T> stan::agrad::binomial\_coefficient\_log | ( | const fvar< T > & | *x1*, |
|  |  | const double | *x2* |
|  | ) |  |  |

inline

Definition at line 45 of file binomial\_coefficient\_log.hpp.

template<typename T >

|  |  |  |  |
| --- | --- | --- | --- |
| fvar<T> stan::agrad::binomial\_coefficient\_log | ( | const double | *x1*, |
|  |  | const fvar< T > & | *x2* |
|  | ) |  |  |

inline

Definition at line 69 of file binomial\_coefficient\_log.hpp.

|  |  |  |  |
| --- | --- | --- | --- |
| double stan::agrad::calculate\_chain | ( | const double & | *x*, |
|  |  | const double & | *val* |
|  | ) |  |  |

inline

Definition at line 8 of file calculate\_chain.hpp.

template<typename T >

|  |  |  |  |  |  |
| --- | --- | --- | --- | --- | --- |
| fvar<T> stan::agrad::cbrt | ( | const fvar< T > & | *x* | ) |  |

inline

Definition at line 16 of file cbrt.hpp.

|  |  |  |  |  |  |
| --- | --- | --- | --- | --- | --- |
| var stan::agrad::cbrt | ( | const stan::agrad::var & | *a* | ) |  |

inline

Returns the cube root of the specified variable (C99).

See boost::math::cbrt() for the double-based version.

The derivative is

.

Parameters
:   |  |  |
    | --- | --- |
    | a | Specified variable. |

Returns
:   Cube root of the variable.

Definition at line 51 of file cbrt.hpp.

template<typename T >

|  |  |  |  |  |  |
| --- | --- | --- | --- | --- | --- |
| fvar<T> stan::agrad::ceil | ( | const fvar< T > & | *x* | ) |  |

inline

Definition at line 15 of file ceil.hpp.

|  |  |  |  |  |  |
| --- | --- | --- | --- | --- | --- |
| var stan::agrad::ceil | ( | const var & | *a* | ) |  |

inline

Return the ceiling of the specified variable (cmath).

The derivative of the ceiling function is defined and zero everywhere but at integers, and we set them to zero for convenience,

.

The ceiling function rounds up. For double values, this is the smallest integral value that is not less than the specified value. Although this function is not differentiable because it is discontinuous at integral values, its gradient is returned as zero everywhere.

Parameters
:   |  |  |
    | --- | --- |
    | a | Input variable. |

Returns
:   Ceiling of the variable.

Definition at line 60 of file ceil.hpp.

template<typename T\_result , class Policy >

|  |  |  |  |
| --- | --- | --- | --- |
| bool stan::agrad::check\_pos\_definite | ( | const char \* | *function*, |
|  |  | const Eigen::Matrix< var, Eigen::Dynamic, Eigen::Dynamic > & | *y*, |
|  |  | const char \* | *name*, |
|  |  | T\_result \* | *result*, |
|  |  | const Policy & |  |
|  | ) |  |  |

inline

Definition at line 15 of file check\_pos\_definite.hpp.

template<typename T , int R1, int C1, int R2, int C2>

|  |  |  |  |
| --- | --- | --- | --- |
| Eigen::Matrix<fvar<T>, 1, C1> stan::agrad::columns\_dot\_product | ( | const Eigen::Matrix< fvar< T >, R1, C1 > & | *v1*, |
|  |  | const Eigen::Matrix< fvar< T >, R2, C2 > & | *v2* |
|  | ) |  |  |

inline

Definition at line 18 of file columns\_dot\_product.hpp.

template<typename T , int R1, int C1, int R2, int C2>

|  |  |  |  |
| --- | --- | --- | --- |
| Eigen::Matrix<fvar<T>, 1, C1> stan::agrad::columns\_dot\_product | ( | const Eigen::Matrix< fvar< T >, R1, C1 > & | *v1*, |
|  |  | const Eigen::Matrix< double, R2, C2 > & | *v2* |
|  | ) |  |  |

inline

Definition at line 34 of file columns\_dot\_product.hpp.

template<typename T , int R1, int C1, int R2, int C2>

|  |  |  |  |
| --- | --- | --- | --- |
| Eigen::Matrix<fvar<T>, 1, C1> stan::agrad::columns\_dot\_product | ( | const Eigen::Matrix< double, R1, C1 > & | *v1*, |
|  |  | const Eigen::Matrix< fvar< T >, R2, C2 > & | *v2* |
|  | ) |  |  |

inline

Definition at line 50 of file columns\_dot\_product.hpp.

template<typename T1 , int R1, int C1, typename T2 , int R2, int C2>

|  |  |  |  |
| --- | --- | --- | --- |
| boost::enable\_if\_c<boost::is\_same<T1,var>::value \|\| boost::is\_same<T2,var>::value, Eigen::Matrix<var, 1, C1> >::type stan::agrad::columns\_dot\_product | ( | const Eigen::Matrix< T1, R1, C1 > & | *v1*, |
|  |  | const Eigen::Matrix< T2, R2, C2 > & | *v2* |
|  | ) |  |  |

inline

Definition at line 261 of file dot\_product.hpp.

template<typename T , int R, int C>

|  |  |  |  |  |  |
| --- | --- | --- | --- | --- | --- |
| Eigen::Matrix<fvar<T>,1,C> stan::agrad::columns\_dot\_self | ( | const Eigen::Matrix< fvar< T >, R, C > & | *x* | ) |  |

inline

Definition at line 15 of file columns\_dot\_self.hpp.

template<int R, int C>

|  |  |  |  |  |  |
| --- | --- | --- | --- | --- | --- |
| Eigen::Matrix<var,1,C> stan::agrad::columns\_dot\_self | ( | const Eigen::Matrix< var, R, C > & | *x* | ) |  |

inline

Returns the dot product of each column of a matrix with itself.

Parameters
:   |  |  |
    | --- | --- |
    | x | Matrix. |

Template Parameters
:   |  |  |
    | --- | --- |
    | T | scalar type |

Definition at line 89 of file dot\_self.hpp.

template<typename T >

|  |  |  |  |  |  |
| --- | --- | --- | --- | --- | --- |
| fvar<T> stan::agrad::cos | ( | const fvar< T > & | *x* | ) |  |

inline

Definition at line 15 of file cos.hpp.

|  |  |  |  |  |  |
| --- | --- | --- | --- | --- | --- |
| var stan::agrad::cos | ( | const var & | *a* | ) |  |

inline

Return the cosine of a radian-scaled variable (cmath).

The derivative is defined by

.

Parameters
:   |  |  |
    | --- | --- |
    | a | Variable for radians of angle. |

Returns
:   Cosine of variable.

Definition at line 50 of file cos.hpp.

template<typename T >

|  |  |  |  |  |  |
| --- | --- | --- | --- | --- | --- |
| fvar<T> stan::agrad::cosh | ( | const fvar< T > & | *x* | ) |  |

inline

Definition at line 15 of file cosh.hpp.

|  |  |  |  |  |  |
| --- | --- | --- | --- | --- | --- |
| var stan::agrad::cosh | ( | const var & | *a* | ) |  |

inline

Return the hyperbolic cosine of the specified variable (cmath).

The derivative is defined by

.

Parameters
:   |  |  |
    | --- | --- |
    | a | Variable. |

Returns
:   Hyperbolic cosine of variable.

Definition at line 51 of file cosh.hpp.

template<typename T , int R, int C>

|  |  |  |  |  |  |
| --- | --- | --- | --- | --- | --- |
| Eigen::Matrix<fvar<T>,C,C> stan::agrad::crossprod | ( | const Eigen::Matrix< fvar< T >, R, C > & | *m* | ) |  |

inline

Definition at line 17 of file crossprod.hpp.

|  |  |  |  |  |  |
| --- | --- | --- | --- | --- | --- |
| matrix\_v stan::agrad::crossprod | ( | const matrix\_v & | *M* | ) |  |

inline

Returns the result of pre-multiplying a matrix by its own transpose.

Parameters
:   |  |  |
    | --- | --- |
    | M | Matrix to multiply. |

Returns
:   Transpose of M times M

Definition at line 17 of file crossprod.hpp.

template<typename T , typename F >

|  |  |  |  |
| --- | --- | --- | --- |
| void stan::agrad::derivative | ( | const F & | *f*, |
|  |  | const T & | *x*, |
|  |  | T & | *fx*, |
|  |  | T & | *dfx\_dx* |
|  | ) |  |  |

Return the derivative of the specified univariate function at the specified argument.

Template Parameters
:   |  |  |
    | --- | --- |
    | T | Argument type |
    | F | Function type |

Parameters
:   |  |  |  |
    | --- | --- | --- |
    | [in] | f | Function |
    | [in] | x | Argument |
    | [out] | fx | Value of function applied to argument |
    | [out] | dfx\_dx | Value of derivative |

Definition at line 25 of file autodiff.hpp.

template<typename T , int R, int C>

|  |  |  |  |  |  |
| --- | --- | --- | --- | --- | --- |
| fvar<T> stan::agrad::determinant | ( | const Eigen::Matrix< fvar< T >, R, C > & | *m* | ) |  |

inline

Definition at line 21 of file determinant.hpp.

template<int R, int C>

|  |  |  |  |  |  |
| --- | --- | --- | --- | --- | --- |
| var stan::agrad::determinant | ( | const Eigen::Matrix< var, R, C > & | *m* | ) |  |

inline

Definition at line 67 of file determinant.hpp.

template<typename T >

|  |  |  |  |  |  |
| --- | --- | --- | --- | --- | --- |
| fvar<T> stan::agrad::digamma | ( | const fvar< T > & | *x* | ) |  |

inline

Definition at line 16 of file digamma.hpp.

|  |  |  |  |  |  |
| --- | --- | --- | --- | --- | --- |
| var stan::agrad::digamma | ( | const stan::agrad::var & | *a* | ) |  |

inline

Definition at line 25 of file digamma.hpp.

template<typename T1 , typename T2 >

|  |  |  |  |
| --- | --- | --- | --- |
| stan::return\_type<T1,T2>::type stan::agrad::divide | ( | const T1 & | *v*, |
|  |  | const T2 & | *c* |
|  | ) |  |  |

inline

Definition at line 17 of file divide.hpp.

template<typename T , int R, int C>

|  |  |  |  |
| --- | --- | --- | --- |
| Eigen::Matrix<fvar<T>,R,C> stan::agrad::divide | ( | const Eigen::Matrix< fvar< T >, R, C > & | *v*, |
|  |  | const fvar< T > & | *c* |
|  | ) |  |  |

inline

Definition at line 23 of file divide.hpp.

|  |  |  |  |
| --- | --- | --- | --- |
| double stan::agrad::divide | ( | double | *x*, |
|  |  | double | *y* |
|  | ) |  |  |

inline

Return the division of the first scalar by the second scalar.

Parameters
:   |  |  |  |
    | --- | --- | --- |
    | [in] | x | Specified vector. |
    | [in] | y | Specified scalar. |

Returns
:   Vector divided by the scalar.

Definition at line 23 of file divide.hpp.

template<typename T1 , typename T2 >

|  |  |  |  |
| --- | --- | --- | --- |
| var stan::agrad::divide | ( | const T1 & | *v*, |
|  |  | const T2 & | *c* |
|  | ) |  |  |

inline

Definition at line 28 of file divide.hpp.

template<typename T , int R, int C>

|  |  |  |  |
| --- | --- | --- | --- |
| Eigen::Matrix<fvar<T>,R,C> stan::agrad::divide | ( | const Eigen::Matrix< fvar< T >, R, C > & | *v*, |
|  |  | const double | *c* |
|  | ) |  |  |

inline

Definition at line 34 of file divide.hpp.

template<typename T1 , typename T2 , int R, int C>

|  |  |  |  |
| --- | --- | --- | --- |
| Eigen::Matrix<var,R,C> stan::agrad::divide | ( | const Eigen::Matrix< T1, R, C > & | *v*, |
|  |  | const T2 & | *c* |
|  | ) |  |  |

inline

Return the division of the specified column vector by the specified scalar.

Parameters
:   |  |  |  |
    | --- | --- | --- |
    | [in] | v | Specified vector. |
    | [in] | c | Specified scalar. |

Returns
:   Vector divided by the scalar.

Definition at line 40 of file divide.hpp.

template<typename T , int R, int C>

|  |  |  |  |
| --- | --- | --- | --- |
| Eigen::Matrix<fvar<T>,R,C> stan::agrad::divide | ( | const Eigen::Matrix< double, R, C > & | *v*, |
|  |  | const fvar< T > & | *c* |
|  | ) |  |  |

inline

Definition at line 46 of file divide.hpp.

template<typename T , int R1, int C1, int R2, int C2>

|  |  |  |  |
| --- | --- | --- | --- |
| fvar<T> stan::agrad::dot\_product | ( | const Eigen::Matrix< fvar< T >, R1, C1 > & | *v1*, |
|  |  | const Eigen::Matrix< fvar< T >, R2, C2 > & | *v2* |
|  | ) |  |  |

inline

Definition at line 20 of file dot\_product.hpp.

template<typename T , int R1, int C1, int R2, int C2>

|  |  |  |  |
| --- | --- | --- | --- |
| fvar<T> stan::agrad::dot\_product | ( | const Eigen::Matrix< fvar< T >, R1, C1 > & | *v1*, |
|  |  | const Eigen::Matrix< double, R2, C2 > & | *v2* |
|  | ) |  |  |

inline

Definition at line 36 of file dot\_product.hpp.

template<typename T , int R1, int C1, int R2, int C2>

|  |  |  |  |
| --- | --- | --- | --- |
| fvar<T> stan::agrad::dot\_product | ( | const Eigen::Matrix< double, R1, C1 > & | *v1*, |
|  |  | const Eigen::Matrix< fvar< T >, R2, C2 > & | *v2* |
|  | ) |  |  |

inline

Definition at line 52 of file dot\_product.hpp.

template<typename T , int R1, int C1, int R2, int C2>

|  |  |  |  |
| --- | --- | --- | --- |
| fvar<T> stan::agrad::dot\_product | ( | const Eigen::Matrix< fvar< T >, R1, C1 > & | *v1*, |
|  |  | const Eigen::Matrix< fvar< T >, R2, C2 > & | *v2*, |
|  |  | size\_type & | *length* |
|  | ) |  |  |

inline

Definition at line 68 of file dot\_product.hpp.

template<typename T , int R1, int C1, int R2, int C2>

|  |  |  |  |
| --- | --- | --- | --- |
| fvar<T> stan::agrad::dot\_product | ( | const Eigen::Matrix< fvar< T >, R1, C1 > & | *v1*, |
|  |  | const Eigen::Matrix< double, R2, C2 > & | *v2*, |
|  |  | size\_type & | *length* |
|  | ) |  |  |

inline

Definition at line 83 of file dot\_product.hpp.

template<typename T , int R1, int C1, int R2, int C2>

|  |  |  |  |
| --- | --- | --- | --- |
| fvar<T> stan::agrad::dot\_product | ( | const Eigen::Matrix< double, R1, C1 > & | *v1*, |
|  |  | const Eigen::Matrix< fvar< T >, R2, C2 > & | *v2*, |
|  |  | size\_type & | *length* |
|  | ) |  |  |

inline

Definition at line 98 of file dot\_product.hpp.

template<typename T >

|  |  |  |  |
| --- | --- | --- | --- |
| fvar<T> stan::agrad::dot\_product | ( | const std::vector< fvar< T > > & | *v1*, |
|  |  | const std::vector< fvar< T > > & | *v2* |
|  | ) |  |  |

inline

Definition at line 113 of file dot\_product.hpp.

template<typename T >

|  |  |  |  |
| --- | --- | --- | --- |
| fvar<T> stan::agrad::dot\_product | ( | const std::vector< double > & | *v1*, |
|  |  | const std::vector< fvar< T > > & | *v2* |
|  | ) |  |  |

inline

Definition at line 126 of file dot\_product.hpp.

template<typename T >

|  |  |  |  |
| --- | --- | --- | --- |
| fvar<T> stan::agrad::dot\_product | ( | const std::vector< fvar< T > > & | *v1*, |
|  |  | const std::vector< double > & | *v2* |
|  | ) |  |  |

inline

Definition at line 139 of file dot\_product.hpp.

template<typename T >

|  |  |  |  |
| --- | --- | --- | --- |
| fvar<T> stan::agrad::dot\_product | ( | const std::vector< fvar< T > > & | *v1*, |
|  |  | const std::vector< fvar< T > > & | *v2*, |
|  |  | size\_type & | *length* |
|  | ) |  |  |

inline

Definition at line 152 of file dot\_product.hpp.

template<typename T >

|  |  |  |  |
| --- | --- | --- | --- |
| fvar<T> stan::agrad::dot\_product | ( | const std::vector< double > & | *v1*, |
|  |  | const std::vector< fvar< T > > & | *v2*, |
|  |  | size\_type & | *length* |
|  | ) |  |  |

inline

Definition at line 164 of file dot\_product.hpp.

template<typename T >

|  |  |  |  |
| --- | --- | --- | --- |
| fvar<T> stan::agrad::dot\_product | ( | const std::vector< fvar< T > > & | *v1*, |
|  |  | const std::vector< double > & | *v2*, |
|  |  | size\_type & | *length* |
|  | ) |  |  |

inline

Definition at line 176 of file dot\_product.hpp.

template<typename T1 , int R1, int C1, typename T2 , int R2, int C2>

|  |  |  |  |
| --- | --- | --- | --- |
| boost::enable\_if\_c<boost::is\_same<T1,var>::value \|\| boost::is\_same<T2,var>::value, var>::type stan::agrad::dot\_product | ( | const Eigen::Matrix< T1, R1, C1 > & | *v1*, |
|  |  | const Eigen::Matrix< T2, R2, C2 > & | *v2* |
|  | ) |  |  |

inline

Returns the dot product.

Parameters
:   |  |  |  |
    | --- | --- | --- |
    | [in] | v1 | First column vector. |
    | [in] | v2 | Second column vector. |

Returns
:   Dot product of the vectors.

Exceptions
:   |  |  |
    | --- | --- |
    | std::domain\_error | if length of v1 is not equal to length of v2. |

Definition at line 213 of file dot\_product.hpp.

template<typename T1 , typename T2 >

|  |  |  |  |
| --- | --- | --- | --- |
| boost::enable\_if\_c<boost::is\_same<T1,var>::value \|\| boost::is\_same<T2,var>::value, var>::type stan::agrad::dot\_product | ( | const T1 \* | *v1*, |
|  |  | const T2 \* | *v2*, |
|  |  | size\_t | *length* |
|  | ) |  |  |

inline

Returns the dot product.

Parameters
:   |  |  |  |
    | --- | --- | --- |
    | [in] | v1 | First array. |
    | [in] | v2 | Second array. |
    | [in] | length | Length of both arrays. |

Returns
:   Dot product of the arrays.

Definition at line 233 of file dot\_product.hpp.

template<typename T1 , typename T2 >

|  |  |  |  |
| --- | --- | --- | --- |
| boost::enable\_if\_c<boost::is\_same<T1,var>::value \|\| boost::is\_same<T2,var>::value, var>::type stan::agrad::dot\_product | ( | const std::vector< T1 > & | *v1*, |
|  |  | const std::vector< T2 > & | *v2* |
|  | ) |  |  |

inline

Returns the dot product.

Parameters
:   |  |  |  |
    | --- | --- | --- |
    | [in] | v1 | First vector. |
    | [in] | v2 | Second vector. |

Returns
:   Dot product of the vectors.

Exceptions
:   |  |  |
    | --- | --- |
    | std::domain\_error | if sizes of v1 and v2 do not match. |

Definition at line 249 of file dot\_product.hpp.

template<typename T , int R, int C>

|  |  |  |  |  |  |
| --- | --- | --- | --- | --- | --- |
| fvar<T> stan::agrad::dot\_self | ( | const Eigen::Matrix< fvar< T >, R, C > & | *v* | ) |  |

inline

Definition at line 16 of file dot\_self.hpp.

template<int R, int C>

|  |  |  |  |  |  |
| --- | --- | --- | --- | --- | --- |
| var stan::agrad::dot\_self | ( | const Eigen::Matrix< var, R, C > & | *v* | ) |  |

inline

Returns the dot product of a vector with itself.

Parameters
:   |  |  |  |
    | --- | --- | --- |
    | [in] | v | Vector. |

Returns
:   Dot product of the vector with itself.

Template Parameters
:   |  |  |
    | --- | --- |
    | R | number of rows or `Eigen::Dynamic` for dynamic; one of R or C must be 1 |
    | C | number of rows or `Eigen::Dyanmic` for dynamic; one of R or C must be 1 |

Definition at line 77 of file dot\_self.hpp.

|  |  |  |  |  |
| --- | --- | --- | --- | --- |
| static bool stan::agrad::empty\_nested | ( |  | ) |  |

inlinestatic

Return true if there is no nested autodiff being executed.

Definition at line 42 of file var\_stack.hpp.

template<typename T >

|  |  |  |  |  |  |
| --- | --- | --- | --- | --- | --- |
| fvar<T> stan::agrad::erf | ( | const fvar< T > & | *x* | ) |  |

inline

Definition at line 17 of file erf.hpp.

|  |  |  |  |  |  |
| --- | --- | --- | --- | --- | --- |
| var stan::agrad::erf | ( | const stan::agrad::var & | *a* | ) |  |

inline

The error function for variables (C99).

For non-variable function, see erf() from math.h

The derivative is

.

Parameters
:   |  |  |
    | --- | --- |
    | a | The variable. |

Returns
:   Error function applied to the variable.

Definition at line 63 of file erf.hpp.

template<typename T >

|  |  |  |  |  |  |
| --- | --- | --- | --- | --- | --- |
| fvar<T> stan::agrad::erfc | ( | const fvar< T > & | *x* | ) |  |

inline

Definition at line 17 of file erfc.hpp.

|  |  |  |  |  |  |
| --- | --- | --- | --- | --- | --- |
| var stan::agrad::erfc | ( | const stan::agrad::var & | *a* | ) |  |

inline

The complementary error function for variables (C99).

For non-variable function, see erfc() from math.h.

The derivative is

.

Parameters
:   |  |  |
    | --- | --- |
    | a | The variable. |

Returns
:   Complementary error function applied to the variable.

Definition at line 63 of file erfc.hpp.

template<typename T >

|  |  |  |  |  |  |
| --- | --- | --- | --- | --- | --- |
| fvar<T> stan::agrad::exp | ( | const fvar< T > & | *x* | ) |  |

inline

Definition at line 16 of file exp.hpp.

|  |  |  |  |  |  |
| --- | --- | --- | --- | --- | --- |
| var stan::agrad::exp | ( | const var & | *a* | ) |  |

inline

Return the exponentiation of the specified variable (cmath).

Parameters
:   |  |  |
    | --- | --- |
    | a | Variable to exponentiate. |

Returns
:   Exponentiated variable.

Definition at line 45 of file exp.hpp.

template<typename T >

|  |  |  |  |  |  |
| --- | --- | --- | --- | --- | --- |
| fvar<T> stan::agrad::exp2 | ( | const fvar< T > & | *x* | ) |  |

inline

Definition at line 16 of file exp2.hpp.

|  |  |  |  |  |  |
| --- | --- | --- | --- | --- | --- |
| var stan::agrad::exp2 | ( | const stan::agrad::var & | *a* | ) |  |

inline

Exponentiation base 2 function for variables (C99).

For non-variable function, see boost::math::exp2().

The derivatie is

.

Parameters
:   |  |  |
    | --- | --- |
    | a | The variable. |

Returns
:   Two to the power of the specified variable.

Definition at line 53 of file exp2.hpp.

template<typename T >

|  |  |  |  |  |  |
| --- | --- | --- | --- | --- | --- |
| fvar<T> stan::agrad::expm1 | ( | const fvar< T > & | *x* | ) |  |

inline

Definition at line 15 of file expm1.hpp.

|  |  |  |  |  |  |
| --- | --- | --- | --- | --- | --- |
| var stan::agrad::expm1 | ( | const stan::agrad::var & | *a* | ) |  |

inline

The exponentiation of the specified variable minus 1 (C99).

For non-variable function, see boost::math::expm1().

The derivative is given by

.

Parameters
:   |  |  |
    | --- | --- |
    | a | The variable. |

Returns
:   Two to the power of the specified variable.

Definition at line 54 of file expm1.hpp.

template<typename T >

|  |  |  |  |  |  |
| --- | --- | --- | --- | --- | --- |
| fvar<T> stan::agrad::fabs | ( | const fvar< T > & | *x* | ) |  |

inline

Definition at line 18 of file fabs.hpp.

|  |  |  |  |  |  |
| --- | --- | --- | --- | --- | --- |
| var stan::agrad::fabs | ( | const var & | *a* | ) |  |

inline

Return the absolute value of the variable (cmath).

Choosing an arbitrary value at the non-differentiable point 0,

.

where  is the signum function, taking values -1 if , 0 if , and 1 if .

The function `abs()` provides the same behavior, with `abs()` defined in stdlib.h and `fabs()` defined in `cmath`. The derivative is 0 if the input is 0.

Returns std::numeric\_limits<double>::quiet\_NaN() for NaN inputs.

[ {fabs}(x) = {cases} |x| & {if } - x \[6pt] {NaN} & {if } x = {NaN} {cases} ]

[ {\,{fabs}(x)}{ x} = {cases} -1 & {if } x < 0 \ 0 & {if } x = 0 \ 1 & {if } x > 0 \[6pt] {NaN} & {if } x = {NaN} {cases} ]

Parameters
:   |  |  |
    | --- | --- |
    | a | Input variable. |

Returns
:   Absolute value of variable.

Definition at line 53 of file fabs.hpp.

template<typename T >

|  |  |  |  |
| --- | --- | --- | --- |
| fvar<T> stan::agrad::falling\_factorial | ( | const fvar< T > & | *x*, |
|  |  | const fvar< T > & | *n* |
|  | ) |  |  |

inline

Definition at line 15 of file falling\_factorial.hpp.

template<typename T >

|  |  |  |  |
| --- | --- | --- | --- |
| fvar<T> stan::agrad::falling\_factorial | ( | const fvar< T > & | *x*, |
|  |  | const double | *n* |
|  | ) |  |  |

inline

Definition at line 26 of file falling\_factorial.hpp.

template<typename T >

|  |  |  |  |
| --- | --- | --- | --- |
| fvar<T> stan::agrad::falling\_factorial | ( | const double | *x*, |
|  |  | const fvar< T > & | *n* |
|  | ) |  |  |

inline

Definition at line 37 of file falling\_factorial.hpp.

|  |  |  |  |
| --- | --- | --- | --- |
| var stan::agrad::falling\_factorial | ( | const var & | *a*, |
|  |  | const double & | *b* |
|  | ) |  |  |

inline

Definition at line 48 of file falling\_factorial.hpp.

|  |  |  |  |
| --- | --- | --- | --- |
| var stan::agrad::falling\_factorial | ( | const var & | *a*, |
|  |  | const var & | *b* |
|  | ) |  |  |

inline

Definition at line 53 of file falling\_factorial.hpp.

|  |  |  |  |
| --- | --- | --- | --- |
| var stan::agrad::falling\_factorial | ( | const double & | *a*, |
|  |  | const var & | *b* |
|  | ) |  |  |

inline

Definition at line 58 of file falling\_factorial.hpp.

template<typename T >

|  |  |  |  |
| --- | --- | --- | --- |
| fvar<T> stan::agrad::fdim | ( | const fvar< T > & | *x1*, |
|  |  | const fvar< T > & | *x2* |
|  | ) |  |  |

inline

Definition at line 15 of file fdim.hpp.

template<typename T >

|  |  |  |  |
| --- | --- | --- | --- |
| fvar<T> stan::agrad::fdim | ( | const fvar< T > & | *x1*, |
|  |  | const double | *x2* |
|  | ) |  |  |

inline

Definition at line 28 of file fdim.hpp.

template<typename T >

|  |  |  |  |
| --- | --- | --- | --- |
| fvar<T> stan::agrad::fdim | ( | const double | *x1*, |
|  |  | const fvar< T > & | *x2* |
|  | ) |  |  |

inline

Definition at line 40 of file fdim.hpp.

|  |  |  |  |
| --- | --- | --- | --- |
| var stan::agrad::fdim | ( | const stan::agrad::var & | *a*, |
|  |  | const stan::agrad::var & | *b* |
|  | ) |  |  |

inline

Return the positive difference between the first variable's the value and the second's (C99).

See stan::math::fdim() for the double-based version.

The partial derivative with respect to the first argument is

if , and

if .

With respect to the second argument, the partial is

if , and

if .

Parameters
:   |  |  |
    | --- | --- |
    | a | First variable. |
    | b | Second variable. |

Returns
:   The positive difference between the first and second variable.

Definition at line 112 of file fdim.hpp.

|  |  |  |  |
| --- | --- | --- | --- |
| var stan::agrad::fdim | ( | const double & | *a*, |
|  |  | const stan::agrad::var & | *b* |
|  | ) |  |  |

inline

Return the positive difference between the first value and the value of the second variable (C99).

See fdim(var,var) for definitions of values and derivatives.

The derivative with respect to the variable is

if , and

if .

Parameters
:   |  |  |
    | --- | --- |
    | a | First value. |
    | b | Second variable. |

Returns
:   The positive difference between the first and second arguments.

Definition at line 137 of file fdim.hpp.

|  |  |  |  |
| --- | --- | --- | --- |
| var stan::agrad::fdim | ( | const stan::agrad::var & | *a*, |
|  |  | const double & | *b* |
|  | ) |  |  |

inline

Return the positive difference between the first variable's value and the second value (C99).

See fdim(var,var) for definitions of values and derivatives.

The derivative with respect to the variable is

if , and

if .

Parameters
:   |  |  |
    | --- | --- |
    | a | First value. |
    | b | Second variable. |

Returns
:   The positive difference between the first and second arguments.

Definition at line 160 of file fdim.hpp.

template<typename T >

|  |  |  |  |  |  |
| --- | --- | --- | --- | --- | --- |
| fvar<T> stan::agrad::floor | ( | const fvar< T > & | *x* | ) |  |

inline

Definition at line 15 of file floor.hpp.

|  |  |  |  |  |  |
| --- | --- | --- | --- | --- | --- |
| var stan::agrad::floor | ( | const var & | *a* | ) |  |

inline

Return the floor of the specified variable (cmath).

The derivative of the floor function is defined and zero everywhere but at integers, so we set these derivatives to zero for convenience,

.

The floor function rounds down. For double values, this is the largest integral value that is not greater than the specified value. Although this function is not differentiable because it is discontinuous at integral values, its gradient is returned as zero everywhere.

Parameters
:   |  |  |
    | --- | --- |
    | a | Input variable. |

Returns
:   Floor of the variable.

Definition at line 60 of file floor.hpp.

template<typename T1 , typename T2 , typename T3 >

|  |  |  |  |
| --- | --- | --- | --- |
| fvar<typename stan::return\_type<T1,T2,T3>::type> stan::agrad::fma | ( | const fvar< T1 > & | *x1*, |
|  |  | const fvar< T2 > & | *x2*, |
|  |  | const fvar< T3 > & | *x3* |
|  | ) |  |  |

inline

The fused multiply-add operation (C99).

This double-based operation delegates to `fma`.

The function is defined by

`fma(a,b,c) = (a * b) + c`.

Parameters
:   |  |  |
    | --- | --- |
    | x1 | First value. |
    | x2 | Second value. |
    | x3 | Third value. |

Returns
:   Product of the first two values plus the third.

Definition at line 62 of file fma.hpp.

template<typename T1 , typename T2 , typename T3 >

|  |  |  |  |
| --- | --- | --- | --- |
| fvar<typename stan::return\_type<T1,T2,T3>::type> stan::agrad::fma | ( | const T1 & | *x1*, |
|  |  | const fvar< T2 > & | *x2*, |
|  |  | const fvar< T3 > & | *x3* |
|  | ) |  |  |

inline

See all-var input signature for details on the function and derivatives.

Definition at line 74 of file fma.hpp.

template<typename T1 , typename T2 , typename T3 >

|  |  |  |  |
| --- | --- | --- | --- |
| fvar<typename stan::return\_type<T1,T2,T3>::type> stan::agrad::fma | ( | const fvar< T1 > & | *x1*, |
|  |  | const T2 & | *x2*, |
|  |  | const fvar< T3 > & | *x3* |
|  | ) |  |  |

inline

See all-var input signature for details on the function and derivatives.

Definition at line 86 of file fma.hpp.

template<typename T1 , typename T2 , typename T3 >

|  |  |  |  |
| --- | --- | --- | --- |
| fvar<typename stan::return\_type<T1,T2,T3>::type> stan::agrad::fma | ( | const fvar< T1 > & | *x1*, |
|  |  | const fvar< T2 > & | *x2*, |
|  |  | const T3 & | *x3* |
|  | ) |  |  |

inline

See all-var input signature for details on the function and derivatives.

Definition at line 98 of file fma.hpp.

template<typename T1 , typename T2 , typename T3 >

|  |  |  |  |
| --- | --- | --- | --- |
| fvar<typename stan::return\_type<T1,T2,T3>::type> stan::agrad::fma | ( | const T1 & | *x1*, |
|  |  | const T2 & | *x2*, |
|  |  | const fvar< T3 > & | *x3* |
|  | ) |  |  |

inline

See all-var input signature for details on the function and derivatives.

Definition at line 110 of file fma.hpp.

template<typename T1 , typename T2 , typename T3 >

|  |  |  |  |
| --- | --- | --- | --- |
| fvar<typename stan::return\_type<T1,T2,T3>::type> stan::agrad::fma | ( | const fvar< T1 > & | *x1*, |
|  |  | const T2 & | *x2*, |
|  |  | const T3 & | *x3* |
|  | ) |  |  |

inline

See all-var input signature for details on the function and derivatives.

Definition at line 122 of file fma.hpp.

|  |  |  |  |
| --- | --- | --- | --- |
| var stan::agrad::fma | ( | const stan::agrad::var & | *a*, |
|  |  | const stan::agrad::var & | *b*, |
|  |  | const stan::agrad::var & | *c* |
|  | ) |  |  |

inline

The fused multiply-add function for three variables (C99).

This function returns the product of the first two arguments plus the third argument.

The double-based version `::fma(double,double,double)` is defined in `<cmath>`.

The partial derivatives are

, and

, and

.

Parameters
:   |  |  |
    | --- | --- |
    | a | First multiplicand. |
    | b | Second multiplicand. |
    | c | Summand. |

Returns
:   Product of the multiplicands plus the summand, ($a \* $b) + $c.

Definition at line 134 of file fma.hpp.

template<typename T1 , typename T2 , typename T3 >

|  |  |  |  |
| --- | --- | --- | --- |
| fvar<typename stan::return\_type<T1,T2,T3>::type> stan::agrad::fma | ( | const T1 & | *x1*, |
|  |  | const fvar< T2 > & | *x2*, |
|  |  | const T3 & | *x3* |
|  | ) |  |  |

inline

See all-var input signature for details on the function and derivatives.

Definition at line 134 of file fma.hpp.

|  |  |  |  |
| --- | --- | --- | --- |
| var stan::agrad::fma | ( | const stan::agrad::var & | *a*, |
|  |  | const stan::agrad::var & | *b*, |
|  |  | const double & | *c* |
|  | ) |  |  |

inline

The fused multiply-add function for two variables and a value (C99).

This function returns the product of the first two arguments plus the third argument.

The double-based version `::fma(double,double,double)` is defined in `<cmath>`.

The partial derivatives are

, and

.

Parameters
:   |  |  |
    | --- | --- |
    | a | First multiplicand. |
    | b | Second multiplicand. |
    | c | Summand. |

Returns
:   Product of the multiplicands plus the summand, ($a \* $b) + $c.

Definition at line 159 of file fma.hpp.

|  |  |  |  |
| --- | --- | --- | --- |
| var stan::agrad::fma | ( | const stan::agrad::var & | *a*, |
|  |  | const double & | *b*, |
|  |  | const stan::agrad::var & | *c* |
|  | ) |  |  |

inline

The fused multiply-add function for a variable, value, and variable (C99).

This function returns the product of the first two arguments plus the third argument.

The double-based version `::fma(double,double,double)` is defined in `<cmath>`.

The partial derivatives are

, and

.

Parameters
:   |  |  |
    | --- | --- |
    | a | First multiplicand. |
    | b | Second multiplicand. |
    | c | Summand. |

Returns
:   Product of the multiplicands plus the summand, ($a \* $b) + $c.

Definition at line 184 of file fma.hpp.

|  |  |  |  |
| --- | --- | --- | --- |
| var stan::agrad::fma | ( | const stan::agrad::var & | *a*, |
|  |  | const double & | *b*, |
|  |  | const double & | *c* |
|  | ) |  |  |

inline

The fused multiply-add function for a variable and two values (C99).

This function returns the product of the first two arguments plus the third argument.

The double-based version `::fma(double,double,double)` is defined in `<cmath>`.

The derivative is

.

Parameters
:   |  |  |
    | --- | --- |
    | a | First multiplicand. |
    | b | Second multiplicand. |
    | c | Summand. |

Returns
:   Product of the multiplicands plus the summand, ($a \* $b) + $c.

Definition at line 207 of file fma.hpp.

|  |  |  |  |
| --- | --- | --- | --- |
| var stan::agrad::fma | ( | const double & | *a*, |
|  |  | const stan::agrad::var & | *b*, |
|  |  | const double & | *c* |
|  | ) |  |  |

inline

The fused multiply-add function for a value, variable, and value (C99).

This function returns the product of the first two arguments plus the third argument.

The double-based version `::fma(double,double,double)` is defined in `<cmath>`.

The derivative is

, and

Parameters
:   |  |  |
    | --- | --- |
    | a | First multiplicand. |
    | b | Second multiplicand. |
    | c | Summand. |

Returns
:   Product of the multiplicands plus the summand, ($a \* $b) + $c.

Definition at line 230 of file fma.hpp.

|  |  |  |  |
| --- | --- | --- | --- |
| var stan::agrad::fma | ( | const double & | *a*, |
|  |  | const double & | *b*, |
|  |  | const stan::agrad::var & | *c* |
|  | ) |  |  |

inline

The fused multiply-add function for two values and a variable, and value (C99).

This function returns the product of the first two arguments plus the third argument.

The double-based version `::fma(double,double,double)` is defined in `<cmath>`.

The derivative is

.

Parameters
:   |  |  |
    | --- | --- |
    | a | First multiplicand. |
    | b | Second multiplicand. |
    | c | Summand. |

Returns
:   Product of the multiplicands plus the summand, ($a \* $b) + $c.

Definition at line 253 of file fma.hpp.

|  |  |  |  |
| --- | --- | --- | --- |
| var stan::agrad::fma | ( | const double & | *a*, |
|  |  | const stan::agrad::var & | *b*, |
|  |  | const stan::agrad::var & | *c* |
|  | ) |  |  |

inline

The fused multiply-add function for a value and two variables (C99).

This function returns the product of the first two arguments plus the third argument.

The double-based version `::fma(double,double,double)` is defined in `<cmath>`.

The partial derivaties are

, and

.

Parameters
:   |  |  |
    | --- | --- |
    | a | First multiplicand. |
    | b | Second multiplicand. |
    | c | Summand. |

Returns
:   Product of the multiplicands plus the summand, ($a \* $b) + $c.

Definition at line 278 of file fma.hpp.

template<typename T >

|  |  |  |  |
| --- | --- | --- | --- |
| fvar<T> stan::agrad::fmax | ( | const fvar< T > & | *x1*, |
|  |  | const fvar< T > & | *x2* |
|  | ) |  |  |

inline

Definition at line 17 of file fmax.hpp.

template<typename T >

|  |  |  |  |
| --- | --- | --- | --- |
| fvar<T> stan::agrad::fmax | ( | const double | *x1*, |
|  |  | const fvar< T > & | *x2* |
|  | ) |  |  |

inline

Definition at line 38 of file fmax.hpp.

template<typename T >

|  |  |  |  |
| --- | --- | --- | --- |
| fvar<T> stan::agrad::fmax | ( | const fvar< T > & | *x1*, |
|  |  | const double | *x2* |
|  | ) |  |  |

inline

Definition at line 59 of file fmax.hpp.

|  |  |  |  |
| --- | --- | --- | --- |
| var stan::agrad::fmax | ( | const stan::agrad::var & | *a*, |
|  |  | const stan::agrad::var & | *b* |
|  | ) |  |  |

inline

Returns the maximum of the two variable arguments (C99).

See boost::math::fmax() for the double-based version.

No new variable implementations are created, with this function defined as if by

`fmax(a,b) = a` if a's value is greater than b's, and .

`fmax(a,b) = b` if b's value is greater than or equal to a's.

Parameters
:   |  |  |
    | --- | --- |
    | a | First variable. |
    | b | Second variable. |

Returns
:   If the first variable's value is larger than the second's, the first variable, otherwise the second variable.

Definition at line 64 of file fmax.hpp.

|  |  |  |  |
| --- | --- | --- | --- |
| var stan::agrad::fmax | ( | const stan::agrad::var & | *a*, |
|  |  | const double & | *b* |
|  | ) |  |  |

inline

Returns the maximum of the variable and scalar, promoting the scalar to a variable if it is larger (C99).

See boost::math::fmax() for the double-based version.

For `fmax(a,b)`, if a's value is greater than b, then a is returned, otherwise a fesh variable implementation wrapping the value b is returned.

Parameters
:   |  |  |
    | --- | --- |
    | a | First variable. |
    | b | Second value |

Returns
:   If the first variable's value is larger than or equal to the second value, the first variable, otherwise the second value promoted to a fresh variable.

Definition at line 101 of file fmax.hpp.

|  |  |  |  |
| --- | --- | --- | --- |
| var stan::agrad::fmax | ( | const double & | *a*, |
|  |  | const stan::agrad::var & | *b* |
|  | ) |  |  |

inline

Returns the maximum of a scalar and variable, promoting the scalar to a variable if it is larger (C99).

See boost::math::fmax() for the double-based version.

For `fmax(a,b)`, if a is greater than b's value, then a fresh variable implementation wrapping a is returned, otherwise b is returned.

Parameters
:   |  |  |
    | --- | --- |
    | a | First value. |
    | b | Second variable. |

Returns
:   If the first value is larger than the second variable's value, return the first value promoted to a variable, otherwise return the second variable.

Definition at line 132 of file fmax.hpp.

template<typename T >

|  |  |  |  |
| --- | --- | --- | --- |
| fvar<T> stan::agrad::fmin | ( | const fvar< T > & | *x1*, |
|  |  | const fvar< T > & | *x2* |
|  | ) |  |  |

inline

Definition at line 17 of file fmin.hpp.

template<typename T >

|  |  |  |  |
| --- | --- | --- | --- |
| fvar<T> stan::agrad::fmin | ( | const double | *x1*, |
|  |  | const fvar< T > & | *x2* |
|  | ) |  |  |

inline

Definition at line 38 of file fmin.hpp.

template<typename T >

|  |  |  |  |
| --- | --- | --- | --- |
| fvar<T> stan::agrad::fmin | ( | const fvar< T > & | *x1*, |
|  |  | const double | *x2* |
|  | ) |  |  |

inline

Definition at line 59 of file fmin.hpp.

|  |  |  |  |
| --- | --- | --- | --- |
| var stan::agrad::fmin | ( | const stan::agrad::var & | *a*, |
|  |  | const stan::agrad::var & | *b* |
|  | ) |  |  |

inline

Returns the minimum of the two variable arguments (C99).

See boost::math::fmin() for the double-based version.

For `fmin(a,b)`, if a's value is less than b's, then a is returned, otherwise b is returned.

Parameters
:   |  |  |
    | --- | --- |
    | a | First variable. |
    | b | Second variable. |

Returns
:   If the first variable's value is smaller than the second's, the first variable, otherwise the second variable.

Definition at line 60 of file fmin.hpp.

|  |  |  |  |
| --- | --- | --- | --- |
| var stan::agrad::fmin | ( | const stan::agrad::var & | *a*, |
|  |  | const double & | *b* |
|  | ) |  |  |

inline

Returns the minimum of the variable and scalar, promoting the scalar to a variable if it is larger (C99).

See boost::math::fmin() for the double-based version.

For `fmin(a,b)`, if a's value is less than b, then a is returned, otherwise a fresh variable wrapping b is returned.

Parameters
:   |  |  |
    | --- | --- |
    | a | First variable. |
    | b | Second value |

Returns
:   If the first variable's value is less than or equal to the second value, the first variable, otherwise the second value promoted to a fresh variable.

Definition at line 95 of file fmin.hpp.

|  |  |  |  |
| --- | --- | --- | --- |
| var stan::agrad::fmin | ( | const double & | *a*, |
|  |  | const stan::agrad::var & | *b* |
|  | ) |  |  |

inline

Returns the minimum of a scalar and variable, promoting the scalar to a variable if it is larger (C99).

See boost::math::fmin() for the double-based version.

For `fmin(a,b)`, if a is less than b's value, then a fresh variable implementation wrapping a is returned, otherwise b is returned.

Parameters
:   |  |  |
    | --- | --- |
    | a | First value. |
    | b | Second variable. |

Returns
:   If the first value is smaller than the second variable's value, return the first value promoted to a variable, otherwise return the second variable.

Definition at line 126 of file fmin.hpp.

template<typename T >

|  |  |  |  |
| --- | --- | --- | --- |
| fvar<T> stan::agrad::fmod | ( | const fvar< T > & | *x1*, |
|  |  | const fvar< T > & | *x2* |
|  | ) |  |  |

inline

Definition at line 16 of file fmod.hpp.

template<typename T >

|  |  |  |  |
| --- | --- | --- | --- |
| fvar<T> stan::agrad::fmod | ( | const fvar< T > & | *x1*, |
|  |  | const double | *x2* |
|  | ) |  |  |

inline

Definition at line 26 of file fmod.hpp.

template<typename T >

|  |  |  |  |
| --- | --- | --- | --- |
| fvar<T> stan::agrad::fmod | ( | const double | *x1*, |
|  |  | const fvar< T > & | *x2* |
|  | ) |  |  |

inline

Definition at line 39 of file fmod.hpp.

|  |  |  |  |
| --- | --- | --- | --- |
| var stan::agrad::fmod | ( | const var & | *a*, |
|  |  | const var & | *b* |
|  | ) |  |  |

inline

Return the floating point remainder after dividing the first variable by the second (cmath).

The partial derivatives with respect to the variables are defined everywhere but where , but we set these to match other values, with

, and

.

Parameters
:   |  |  |
    | --- | --- |
    | a | First variable. |
    | b | Second variable. |

Returns
:   Floating pointer remainder of dividing the first variable by the second.

Definition at line 105 of file fmod.hpp.

|  |  |  |  |
| --- | --- | --- | --- |
| var stan::agrad::fmod | ( | const var & | *a*, |
|  |  | const double | *b* |
|  | ) |  |  |

inline

Return the floating point remainder after dividing the the first variable by the second scalar (cmath).

The derivative with respect to the variable is

.

Parameters
:   |  |  |
    | --- | --- |
    | a | First variable. |
    | b | Second scalar. |

Returns
:   Floating pointer remainder of dividing the first variable by the second scalar.

Definition at line 122 of file fmod.hpp.

|  |  |  |  |
| --- | --- | --- | --- |
| var stan::agrad::fmod | ( | const double | *a*, |
|  |  | const var & | *b* |
|  | ) |  |  |

inline

Return the floating point remainder after dividing the first scalar by the second variable (cmath).

The derivative with respect to the variable is

.

Parameters
:   |  |  |
    | --- | --- |
    | a | First scalar. |
    | b | Second variable. |

Returns
:   Floating pointer remainder of dividing first scalar by the second variable.

Definition at line 139 of file fmod.hpp.

template<typename T >

|  |  |  |  |
| --- | --- | --- | --- |
| fvar<T> stan::agrad::gamma\_p | ( | const fvar< T > & | *x1*, |
|  |  | const fvar< T > & | *x2* |
|  | ) |  |  |

inline

Definition at line 16 of file gamma\_p.hpp.

template<typename T >

|  |  |  |  |
| --- | --- | --- | --- |
| fvar<T> stan::agrad::gamma\_p | ( | const fvar< T > & | *x1*, |
|  |  | const double | *x2* |
|  | ) |  |  |

inline

Definition at line 52 of file gamma\_p.hpp.

template<typename T >

|  |  |  |  |
| --- | --- | --- | --- |
| fvar<T> stan::agrad::gamma\_p | ( | const double | *x1*, |
|  |  | const fvar< T > & | *x2* |
|  | ) |  |  |

inline

Definition at line 87 of file gamma\_p.hpp.

|  |  |  |  |
| --- | --- | --- | --- |
| var stan::agrad::gamma\_p | ( | const stan::agrad::var & | *a*, |
|  |  | const stan::agrad::var & | *b* |
|  | ) |  |  |

inline

Definition at line 91 of file gamma\_p.hpp.

|  |  |  |  |
| --- | --- | --- | --- |
| var stan::agrad::gamma\_p | ( | const stan::agrad::var & | *a*, |
|  |  | const double & | *b* |
|  | ) |  |  |

inline

Definition at line 96 of file gamma\_p.hpp.

|  |  |  |  |
| --- | --- | --- | --- |
| var stan::agrad::gamma\_p | ( | const double & | *a*, |
|  |  | const stan::agrad::var & | *b* |
|  | ) |  |  |

inline

Definition at line 101 of file gamma\_p.hpp.

template<typename T >

|  |  |  |  |
| --- | --- | --- | --- |
| fvar<T> stan::agrad::gamma\_q | ( | const fvar< T > & | *x1*, |
|  |  | const fvar< T > & | *x2* |
|  | ) |  |  |

inline

Definition at line 15 of file gamma\_q.hpp.

template<typename T >

|  |  |  |  |
| --- | --- | --- | --- |
| fvar<T> stan::agrad::gamma\_q | ( | const fvar< T > & | *x1*, |
|  |  | const double | *x2* |
|  | ) |  |  |

inline

Definition at line 51 of file gamma\_q.hpp.

|  |  |  |  |
| --- | --- | --- | --- |
| var stan::agrad::gamma\_q | ( | const stan::agrad::var & | *a*, |
|  |  | const stan::agrad::var & | *b* |
|  | ) |  |  |

inline

Definition at line 61 of file gamma\_q.hpp.

|  |  |  |  |
| --- | --- | --- | --- |
| var stan::agrad::gamma\_q | ( | const stan::agrad::var & | *a*, |
|  |  | const double & | *b* |
|  | ) |  |  |

inline

Definition at line 66 of file gamma\_q.hpp.

|  |  |  |  |
| --- | --- | --- | --- |
| var stan::agrad::gamma\_q | ( | const double & | *a*, |
|  |  | const stan::agrad::var & | *b* |
|  | ) |  |  |

inline

Definition at line 71 of file gamma\_q.hpp.

template<typename T >

|  |  |  |  |
| --- | --- | --- | --- |
| fvar<T> stan::agrad::gamma\_q | ( | const double | *x1*, |
|  |  | const fvar< T > & | *x2* |
|  | ) |  |  |

inline

Definition at line 86 of file gamma\_q.hpp.

|  |  |  |  |  |  |
| --- | --- | --- | --- | --- | --- |
| static void stan::agrad::grad | ( | chainable \* | *vi* | ) |  |

static

|  |  |  |  |
| --- | --- | --- | --- |
| void stan::agrad::grad | ( | var & | *v*, |
|  |  | Eigen::Matrix< var, Eigen::Dynamic, 1 > & | *x*, |
|  |  | Eigen::VectorXd & | *g* |
|  | ) |  |  |

Propagate chain rule to calculate gradients starting from the specified variable.

Resizes the input vector to be the correct size.

The grad() function does not itself recover any memory. use `agrad::recover_memory()` or `agrad::recover_memory_nested()`, defined in , defined in agrad/rev/var\_stack.hpp, to recover memory.

Parameters
:   |  |  |  |
    | --- | --- | --- |
    | [in] | v | Value of function being differentiated |
    | [in] | x | Variables being differentiated with respect to |
    | [out] | g | Gradient, d/dx v, evaluated at x. |

Definition at line 27 of file grad.hpp.

|  |  |  |  |  |  |
| --- | --- | --- | --- | --- | --- |
| static void stan::agrad::grad | ( | chainable \* | *vi* | ) |  |

static

Compute the gradient for all variables starting from the specified root variable implementation.

Does not recover memory. This chainable variable's adjoint is initialized using the method `init_dependent()` and then the chain rule is applied working down the stack from this chainable and calling each chainable's `chain()` method in turn.

This function computes a nested gradient only going back as far as the last nesting.

This function does not recover any memory from the computation.

Parameters
:   |  |  |
    | --- | --- |
    | vi | Variable implementation for root of partial derivative propagation. |

Definition at line 110 of file chainable.hpp.

template<typename F >

|  |  |  |  |
| --- | --- | --- | --- |
| void stan::agrad::grad\_tr\_mat\_times\_hessian | ( | const F & | *f*, |
|  |  | const Eigen::Matrix< double, Eigen::Dynamic, 1 > & | *x*, |
|  |  | const Eigen::Matrix< double, Eigen::Dynamic, Eigen::Dynamic > & | *M*, |
|  |  | Eigen::Matrix< double, Eigen::Dynamic, 1 > & | *grad\_tr\_MH* |
|  | ) |  |  |

Definition at line 321 of file autodiff.hpp.

template<typename F >

|  |  |  |  |
| --- | --- | --- | --- |
| void stan::agrad::gradient | ( | const F & | *f*, |
|  |  | const Eigen::Matrix< double, Eigen::Dynamic, 1 > & | *x*, |
|  |  | double & | *fx*, |
|  |  | Eigen::Matrix< double, Eigen::Dynamic, 1 > & | *grad\_fx* |
|  | ) |  |  |

Calculate the value and the gradient of the specified function at the specified argument.

The functor must implement

`stan::agrad::var operator()(const Eigen::Matrix<stan::agrad::var,Eigen::Dynamic,1>&)`

using only operations that are defined for `stan::agrad::var`. This latter constraint usually requires the functions to be defined in terms of the libraries defined in Stan or in terms of functions with appropriately general namespace imports that eventually depend on functions defined in Stan.

Time and memory usage is on the order of the size of the fully unfolded expression for the function applied to the argument, independently of dimension.

Template Parameters
:   |  |  |
    | --- | --- |
    | F | Type of function |

Parameters
:   |  |  |  |
    | --- | --- | --- |
    | [in] | f | Function |
    | [in] | x | Argument to function |
    | [out] | fx | Function applied to argument |
    | [out] | grad\_fx | Gradient of function at argument |

Definition at line 93 of file autodiff.hpp.

template<typename T , typename F >

|  |  |  |  |
| --- | --- | --- | --- |
| void stan::agrad::gradient | ( | const F & | *f*, |
|  |  | const Eigen::Matrix< T, Eigen::Dynamic, 1 > & | *x*, |
|  |  | T & | *fx*, |
|  |  | Eigen::Matrix< T, Eigen::Dynamic, 1 > & | *grad\_fx* |
|  | ) |  |  |

Definition at line 117 of file autodiff.hpp.

template<typename T1 , typename T2 , typename F >

|  |  |  |  |
| --- | --- | --- | --- |
| void stan::agrad::gradient\_dot\_vector | ( | const F & | *f*, |
|  |  | const Eigen::Matrix< T1, Eigen::Dynamic, 1 > & | *x*, |
|  |  | const Eigen::Matrix< T2, Eigen::Dynamic, 1 > & | *v*, |
|  |  | T1 & | *fx*, |
|  |  | T1 & | *grad\_fx\_dot\_v* |
|  | ) |  |  |

Definition at line 252 of file autodiff.hpp.

template<typename F >

|  |  |  |  |
| --- | --- | --- | --- |
| void stan::agrad::hessian | ( | const F & | *f*, |
|  |  | const Eigen::Matrix< double, Eigen::Dynamic, 1 > & | *x*, |
|  |  | double & | *fx*, |
|  |  | Eigen::Matrix< double, Eigen::Dynamic, 1 > & | *grad*, |
|  |  | Eigen::Matrix< double, Eigen::Dynamic, Eigen::Dynamic > & | *H* |
|  | ) |  |  |

Definition at line 194 of file autodiff.hpp.

template<typename T , typename F >

|  |  |  |  |
| --- | --- | --- | --- |
| void stan::agrad::hessian | ( | const F & | *f*, |
|  |  | const Eigen::Matrix< T, Eigen::Dynamic, 1 > & | *x*, |
|  |  | T & | *fx*, |
|  |  | Eigen::Matrix< T, Eigen::Dynamic, 1 > & | *grad*, |
|  |  | Eigen::Matrix< T, Eigen::Dynamic, Eigen::Dynamic > & | *H* |
|  | ) |  |  |

Definition at line 223 of file autodiff.hpp.

template<typename F >

|  |  |  |  |
| --- | --- | --- | --- |
| void stan::agrad::hessian\_times\_vector | ( | const F & | *f*, |
|  |  | const Eigen::Matrix< double, Eigen::Dynamic, 1 > & | *x*, |
|  |  | const Eigen::Matrix< double, Eigen::Dynamic, 1 > & | *v*, |
|  |  | double & | *fx*, |
|  |  | Eigen::Matrix< double, Eigen::Dynamic, 1 > & | *Hv* |
|  | ) |  |  |

Definition at line 274 of file autodiff.hpp.

template<typename T , typename F >

|  |  |  |  |
| --- | --- | --- | --- |
| void stan::agrad::hessian\_times\_vector | ( | const F & | *f*, |
|  |  | const Eigen::Matrix< T, Eigen::Dynamic, 1 > & | *x*, |
|  |  | const Eigen::Matrix< T, Eigen::Dynamic, 1 > & | *v*, |
|  |  | T & | *fx*, |
|  |  | Eigen::Matrix< T, Eigen::Dynamic, 1 > & | *Hv* |
|  | ) |  |  |

Definition at line 304 of file autodiff.hpp.

template<typename T >

|  |  |  |  |
| --- | --- | --- | --- |
| fvar<T> stan::agrad::hypot | ( | const fvar< T > & | *x1*, |
|  |  | const fvar< T > & | *x2* |
|  | ) |  |  |

inline

Definition at line 16 of file hypot.hpp.

template<typename T >

|  |  |  |  |
| --- | --- | --- | --- |
| fvar<T> stan::agrad::hypot | ( | const fvar< T > & | *x1*, |
|  |  | const double | *x2* |
|  | ) |  |  |

inline

Definition at line 27 of file hypot.hpp.

template<typename T >

|  |  |  |  |
| --- | --- | --- | --- |
| fvar<T> stan::agrad::hypot | ( | const double | *x1*, |
|  |  | const fvar< T > & | *x2* |
|  | ) |  |  |

inline

Definition at line 38 of file hypot.hpp.

|  |  |  |  |
| --- | --- | --- | --- |
| var stan::agrad::hypot | ( | const stan::agrad::var & | *a*, |
|  |  | const stan::agrad::var & | *b* |
|  | ) |  |  |

inline

Returns the length of the hypoteneuse of a right triangle with sides of the specified lengths (C99).

See boost::math::hypot() for double-based function.

The partial derivatives are given by

, and

.

Parameters
:   |  |  |
    | --- | --- |
    | a | Length of first side. |
    | b | Length of second side. |

Returns
:   Length of hypoteneuse.

Definition at line 54 of file hypot.hpp.

|  |  |  |  |
| --- | --- | --- | --- |
| var stan::agrad::hypot | ( | const stan::agrad::var & | *a*, |
|  |  | const double & | *b* |
|  | ) |  |  |

inline

Returns the length of the hypoteneuse of a right triangle with sides of the specified lengths (C99).

See boost::math::hypot() for double-based function.

The derivative is

.

Parameters
:   |  |  |
    | --- | --- |
    | a | Length of first side. |
    | b | Length of second side. |

Returns
:   Length of hypoteneuse.

Definition at line 73 of file hypot.hpp.

|  |  |  |  |
| --- | --- | --- | --- |
| var stan::agrad::hypot | ( | const double & | *a*, |
|  |  | const stan::agrad::var & | *b* |
|  | ) |  |  |

inline

Returns the length of the hypoteneuse of a right triangle with sides of the specified lengths (C99).

See boost::math::hypot() for double-based function.

The derivative is

.

Parameters
:   |  |  |
    | --- | --- |
    | a | Length of first side. |
    | b | Length of second side. |

Returns
:   Length of hypoteneuse.

Definition at line 119 of file hypot.hpp.

|  |  |  |  |
| --- | --- | --- | --- |
| var stan::agrad::ibeta | ( | const var & | *a*, |
|  |  | const var & | *b*, |
|  |  | const var & | *x* |
|  | ) |  |  |

inline

The normalized incomplete beta function of a, b, and x.

Used to compute the cumulative density function for the beta distribution.

Partial derivatives are those specified by wolfram alpha. The values were checked using both finite differences and by independent code for calculating the derivatives found in JSS (paper by Boik and Robison-Cox).

Parameters
:   |  |  |
    | --- | --- |
    | a | Shape parameter. |
    | b | Shape parameter. |
    | x | Random variate. |

Returns
:   The normalized incomplete beta function.

Exceptions
:   |  |  |
    | --- | --- |
    | if | any argument is NaN. |

Definition at line 223 of file ibeta.hpp.

|  |  |  |  |
| --- | --- | --- | --- |
| var stan::agrad::if\_else | ( | bool | *c*, |
|  |  | const var & | *y\_true*, |
|  |  | const var & | *y\_false* |
|  | ) |  |  |

inline

If the specified condition is true, return the first variable, otherwise return the second variable.

Parameters
:   |  |  |
    | --- | --- |
    | c | Boolean condition. |
    | y\_true | Variable to return if condition is true. |
    | y\_false | Variable to return if condition is false. |

Definition at line 17 of file if\_else.hpp.

|  |  |  |  |
| --- | --- | --- | --- |
| var stan::agrad::if\_else | ( | bool | *c*, |
|  |  | double | *y\_true*, |
|  |  | const var & | *y\_false* |
|  | ) |  |  |

inline

If the specified condition is true, return a new variable constructed from the first scalar, otherwise return the second variable.

Parameters
:   |  |  |
    | --- | --- |
    | c | Boolean condition. |
    | y\_true | Value to promote to variable and return if condition is true. |
    | y\_false | Variable to return if condition is false. |

Definition at line 29 of file if\_else.hpp.

|  |  |  |  |
| --- | --- | --- | --- |
| var stan::agrad::if\_else | ( | bool | *c*, |
|  |  | const var & | *y\_true*, |
|  |  | const double | *y\_false* |
|  | ) |  |  |

inline

If the specified condition is true, return the first variable, otherwise return a new variable constructed from the second scalar.

Parameters
:   |  |  |
    | --- | --- |
    | c | Boolean condition. |
    | y\_true | Variable to return if condition is true. |
    | y\_false | Value to promote to variable and return if condition is false. |

Definition at line 44 of file if\_else.hpp.

|  |  |  |  |
| --- | --- | --- | --- |
| void stan::agrad::initialize\_variable | ( | var & | *variable*, |
|  |  | const var & | *value* |
|  | ) |  |  |

inline

Initialize variable to value.

(Function may look pointless, but its needed to bottom out recursion.)

Definition at line 15 of file initialize\_variable.hpp.

template<int R, int C>

|  |  |  |  |
| --- | --- | --- | --- |
| void stan::agrad::initialize\_variable | ( | Eigen::Matrix< var, R, C > & | *matrix*, |
|  |  | const var & | *value* |
|  | ) |  |  |

inline

Initialize every cell in the matrix to the specified value.

Definition at line 24 of file initialize\_variable.hpp.

template<typename T >

|  |  |  |  |
| --- | --- | --- | --- |
| void stan::agrad::initialize\_variable | ( | std::vector< T > & | *variables*, |
|  |  | const var & | *value* |
|  | ) |  |  |

inline

Initialize the variables in the standard vector recursively.

Definition at line 33 of file initialize\_variable.hpp.

template<typename T >

|  |  |  |  |  |  |
| --- | --- | --- | --- | --- | --- |
| fvar<T> stan::agrad::inv | ( | const fvar< T > & | *x* | ) |  |

inline

Definition at line 15 of file inv.hpp.

|  |  |  |  |  |  |
| --- | --- | --- | --- | --- | --- |
| var stan::agrad::inv | ( | const var & | *a* | ) |  |

inline

Definition at line 43 of file inv.hpp.

template<typename T >

|  |  |  |  |  |  |
| --- | --- | --- | --- | --- | --- |
| fvar<T> stan::agrad::inv\_cloglog | ( | const fvar< T > & | *x* | ) |  |

inline

Definition at line 15 of file inv\_cloglog.hpp.

|  |  |  |  |  |  |
| --- | --- | --- | --- | --- | --- |
| var stan::agrad::inv\_cloglog | ( | const stan::agrad::var & | *a* | ) |  |

inline

Return the inverse complementary log-log function applied specified variable (stan).

See stan::math::inv\_cloglog() for the double-based version.

The derivative is given by

.

Parameters
:   |  |  |
    | --- | --- |
    | a | Variable argument. |

Returns
:   The inverse complementary log-log of the specified argument.

Definition at line 37 of file inv\_cloglog.hpp.

template<typename T >

|  |  |  |  |  |  |
| --- | --- | --- | --- | --- | --- |
| fvar<T> stan::agrad::inv\_logit | ( | const fvar< T > & | *x* | ) |  |

inline

Definition at line 15 of file inv\_logit.hpp.

|  |  |  |  |  |  |
| --- | --- | --- | --- | --- | --- |
| var stan::agrad::inv\_logit | ( | const stan::agrad::var & | *a* | ) |  |

inline

The inverse logit function for variables (stan).

See stan::math::inv\_logit() for the double-based version.

The derivative of inverse logit is

.

Parameters
:   |  |  |
    | --- | --- |
    | a | Argument variable. |

Returns
:   Inverse logit of argument.

Definition at line 35 of file inv\_logit.hpp.

template<typename T >

|  |  |  |  |  |  |
| --- | --- | --- | --- | --- | --- |
| fvar<T> stan::agrad::inv\_sqrt | ( | const fvar< T > & | *x* | ) |  |

inline

Definition at line 15 of file inv\_sqrt.hpp.

|  |  |  |  |  |  |
| --- | --- | --- | --- | --- | --- |
| var stan::agrad::inv\_sqrt | ( | const var & | *a* | ) |  |

inline

Definition at line 43 of file inv\_sqrt.hpp.

template<typename T >

|  |  |  |  |  |  |
| --- | --- | --- | --- | --- | --- |
| fvar<T> stan::agrad::inv\_square | ( | const fvar< T > & | *x* | ) |  |

inline

Definition at line 15 of file inv\_square.hpp.

|  |  |  |  |  |  |
| --- | --- | --- | --- | --- | --- |
| var stan::agrad::inv\_square | ( | const var & | *a* | ) |  |

inline

Definition at line 43 of file inv\_square.hpp.

template<typename T , int R, int C>

|  |  |  |  |  |  |
| --- | --- | --- | --- | --- | --- |
| Eigen::Matrix<fvar<T>,R,C> stan::agrad::inverse | ( | const Eigen::Matrix< fvar< T >, R, C > & | *m* | ) |  |

inline

Definition at line 21 of file inverse.hpp.

template<typename T >

|  |  |  |  |  |  |
| --- | --- | --- | --- | --- | --- |
| int stan::agrad::is\_inf | ( | const fvar< T > & | *x* | ) |  |

inline

Returns 1 if the input's value is infinite and 0 otherwise.

Delegates to `stan::math::is_inf`.

Parameters
:   |  |  |
    | --- | --- |
    | x | Value to test. |

Returns
:   `1` if the value is infinite and `0` otherwise.

Definition at line 22 of file is\_inf.hpp.

|  |  |  |  |  |  |
| --- | --- | --- | --- | --- | --- |
| int stan::agrad::is\_inf | ( | const var & | *v* | ) |  |

inline

Returns 1 if the input's value is infinite and 0 otherwise.

Delegates to `stan::math::is_inf`.

Parameters
:   |  |  |
    | --- | --- |
    | x | Value to test. |

Returns
:   `1` if the value is infinite and `0` otherwise.

Definition at line 23 of file is\_inf.hpp.

template<typename T >

|  |  |  |  |  |  |
| --- | --- | --- | --- | --- | --- |
| int stan::agrad::is\_nan | ( | const fvar< T > & | *x* | ) |  |

inline

Returns 1 if the input's value is NaN and 0 otherwise.

Delegates to `stan::math::is_nan`.

Parameters
:   |  |  |
    | --- | --- |
    | x | Value to test. |

Returns
:   `1` if the value is NaN and `0` otherwise.

Definition at line 22 of file is\_nan.hpp.

|  |  |  |  |  |  |
| --- | --- | --- | --- | --- | --- |
| int stan::agrad::is\_nan | ( | const var & | *v* | ) |  |

inline

Returns 1 if the input's value is NaN and 0 otherwise.

Delegates to `stan::math::is_nan`.

Parameters
:   |  |  |
    | --- | --- |
    | x | Value to test. |

Returns
:   `1` if the value is NaN and `0` otherwise.

Definition at line 23 of file is\_nan.hpp.

|  |  |  |  |  |  |
| --- | --- | --- | --- | --- | --- |
| bool stan::agrad::is\_uninitialized | ( | var | *x* | ) |  |

inline

Returns `true` if the specified variable is uninitialized.

This overload of the `stan::math::is_uninitialized()` function delegates the return to the `is_uninitialized()` method on the specified variable.

Parameters
:   |  |  |
    | --- | --- |
    | x | Object to test. |

Returns
:   `true` if the specified object is uninitialized.

Definition at line 25 of file is\_uninitialized.hpp.

template<typename F >

|  |  |  |  |
| --- | --- | --- | --- |
| void stan::agrad::jacobian | ( | const F & | *f*, |
|  |  | const Eigen::Matrix< double, Eigen::Dynamic, 1 > & | *x*, |
|  |  | Eigen::Matrix< double, Eigen::Dynamic, 1 > & | *fx*, |
|  |  | Eigen::Matrix< double, Eigen::Dynamic, Eigen::Dynamic > & | *J* |
|  | ) |  |  |

Definition at line 135 of file autodiff.hpp.

template<typename T , typename F >

|  |  |  |  |
| --- | --- | --- | --- |
| void stan::agrad::jacobian | ( | const F & | *f*, |
|  |  | const Eigen::Matrix< T, Eigen::Dynamic, 1 > & | *x*, |
|  |  | Eigen::Matrix< T, Eigen::Dynamic, 1 > & | *fx*, |
|  |  | Eigen::Matrix< T, Eigen::Dynamic, Eigen::Dynamic > & | *J* |
|  | ) |  |  |

Definition at line 166 of file autodiff.hpp.

template<typename T >

|  |  |  |  |
| --- | --- | --- | --- |
| fvar<T> stan::agrad::lbeta | ( | const fvar< T > & | *x1*, |
|  |  | const fvar< T > & | *x2* |
|  | ) |  |  |

inline

Definition at line 16 of file lbeta.hpp.

template<typename T >

|  |  |  |  |
| --- | --- | --- | --- |
| fvar<T> stan::agrad::lbeta | ( | const double | *x1*, |
|  |  | const fvar< T > & | *x2* |
|  | ) |  |  |

inline

Definition at line 28 of file lbeta.hpp.

template<typename T >

|  |  |  |  |
| --- | --- | --- | --- |
| fvar<T> stan::agrad::lbeta | ( | const fvar< T > & | *x1*, |
|  |  | const double | *x2* |
|  | ) |  |  |

inline

Definition at line 38 of file lbeta.hpp.

template<typename T >

|  |  |  |  |  |  |
| --- | --- | --- | --- | --- | --- |
| fvar<T> stan::agrad::lgamma | ( | const fvar< T > & | *x* | ) |  |

inline

Definition at line 15 of file lgamma.hpp.

|  |  |  |  |  |  |
| --- | --- | --- | --- | --- | --- |
| var stan::agrad::lgamma | ( | const stan::agrad::var & | *a* | ) |  |

inline

The log gamma function for variables (C99).

The derivatie is the digamma function,

.

Parameters
:   |  |  |
    | --- | --- |
    | a | The variable. |

Returns
:   Log gamma of the variable.

Definition at line 36 of file lgamma.hpp.

template<typename T >

|  |  |  |  |
| --- | --- | --- | --- |
| fvar<typename stan::return\_type<T,int>::type> stan::agrad::lmgamma | ( | int | *x1*, |
|  |  | const fvar< T > & | *x2* |
|  | ) |  |  |

inline

Definition at line 16 of file lmgamma.hpp.

|  |  |  |  |
| --- | --- | --- | --- |
| var stan::agrad::lmgamma | ( | int | *a*, |
|  |  | const stan::agrad::var & | *b* |
|  | ) |  |  |

inline

Definition at line 29 of file lmgamma.hpp.

template<typename T >

|  |  |  |  |  |  |
| --- | --- | --- | --- | --- | --- |
| fvar<T> stan::agrad::log | ( | const fvar< T > & | *x* | ) |  |

inline

Definition at line 15 of file log.hpp.

|  |  |  |  |  |  |
| --- | --- | --- | --- | --- | --- |
| var stan::agrad::log | ( | const var & | *a* | ) |  |

inline

Return the natural log of the specified variable (cmath).

The derivative is defined by

.

Parameters
:   |  |  |
    | --- | --- |
    | a | Variable whose log is taken. |

Returns
:   Natural log of variable.

Definition at line 51 of file log.hpp.

template<typename T >

|  |  |  |  |  |  |
| --- | --- | --- | --- | --- | --- |
| fvar<T> stan::agrad::log10 | ( | const fvar< T > & | *x* | ) |  |

inline

Definition at line 15 of file log10.hpp.

|  |  |  |  |  |  |
| --- | --- | --- | --- | --- | --- |
| var stan::agrad::log10 | ( | const var & | *a* | ) |  |

inline

Return the base 10 log of the specified variable (cmath).

The derivative is defined by

.

Parameters
:   |  |  |
    | --- | --- |
    | a | Variable whose log is taken. |

Returns
:   Base 10 log of variable.

Definition at line 55 of file log10.hpp.

template<typename T >

|  |  |  |  |  |  |
| --- | --- | --- | --- | --- | --- |
| fvar<T> stan::agrad::log1m | ( | const fvar< T > & | *x* | ) |  |

inline

Definition at line 16 of file log1m.hpp.

|  |  |  |  |  |  |
| --- | --- | --- | --- | --- | --- |
| var stan::agrad::log1m | ( | const stan::agrad::var & | *a* | ) |  |

inline

The log (1 - x) function for variables.

The derivative is given by

.

Parameters
:   |  |  |
    | --- | --- |
    | a | The variable. |

Returns
:   The variable representing log of 1 minus the variable.

Definition at line 33 of file log1m.hpp.

template<typename T >

|  |  |  |  |  |  |
| --- | --- | --- | --- | --- | --- |
| fvar<T> stan::agrad::log1m\_exp | ( | const fvar< T > & | *x* | ) |  |

inline

Definition at line 17 of file log1m\_exp.hpp.

|  |  |  |  |  |  |
| --- | --- | --- | --- | --- | --- |
| var stan::agrad::log1m\_exp | ( | const stan::agrad::var & | *a* | ) |  |

inline

Return the log of 1 minus the exponential of the specified variable.

Definition at line 31 of file log1m\_exp.hpp.

template<typename T >

|  |  |  |  |  |  |
| --- | --- | --- | --- | --- | --- |
| fvar<T> stan::agrad::log1m\_inv\_logit | ( | const fvar< T > & | *x* | ) |  |

inline

Definition at line 15 of file log1m\_inv\_logit.hpp.

template<typename T >

|  |  |  |  |  |  |
| --- | --- | --- | --- | --- | --- |
| fvar<T> stan::agrad::log1p | ( | const fvar< T > & | *x* | ) |  |

inline

Definition at line 16 of file log1p.hpp.

|  |  |  |  |  |  |
| --- | --- | --- | --- | --- | --- |
| var stan::agrad::log1p | ( | const stan::agrad::var & | *a* | ) |  |

inline

The log (1 + x) function for variables (C99).

The derivative is given by

.

Parameters
:   |  |  |
    | --- | --- |
    | a | The variable. |

Returns
:   The log of 1 plus the variable.

Definition at line 35 of file log1p.hpp.

template<typename T >

|  |  |  |  |  |  |
| --- | --- | --- | --- | --- | --- |
| fvar<T> stan::agrad::log1p\_exp | ( | const fvar< T > & | *x* | ) |  |

inline

Definition at line 15 of file log1p\_exp.hpp.

|  |  |  |  |  |  |
| --- | --- | --- | --- | --- | --- |
| var stan::agrad::log1p\_exp | ( | const stan::agrad::var & | *a* | ) |  |

inline

Return the log of 1 plus the exponential of the specified variable.

Definition at line 29 of file log1p\_exp.hpp.

template<typename T >

|  |  |  |  |  |  |
| --- | --- | --- | --- | --- | --- |
| fvar<T> stan::agrad::log2 | ( | const fvar< T > & | *x* | ) |  |

inline

Definition at line 17 of file log2.hpp.

|  |  |  |  |  |  |
| --- | --- | --- | --- | --- | --- |
| var stan::agrad::log2 | ( | const stan::agrad::var & | *a* | ) |  |

inline

Returns the base 2 logarithm of the specified variable (C99).

See stan::math::log2() for the double-based version.

The derivative is

.

Parameters
:   |  |  |
    | --- | --- |
    | a | Specified variable. |

Returns
:   Base 2 logarithm of the variable.

Definition at line 54 of file log2.hpp.

template<typename T , int R, int C>

|  |  |  |  |  |  |
| --- | --- | --- | --- | --- | --- |
| fvar<T> stan::agrad::log\_determinant | ( | const Eigen::Matrix< fvar< T >, R, C > & | *m* | ) |  |

inline

Definition at line 20 of file log\_determinant.hpp.

template<int R, int C>

|  |  |  |  |  |  |
| --- | --- | --- | --- | --- | --- |
| var stan::agrad::log\_determinant | ( | const Eigen::Matrix< var, R, C > & | *m* | ) |  |

inline

Definition at line 69 of file log\_determinant.hpp.

template<int R, int C>

|  |  |  |  |  |  |
| --- | --- | --- | --- | --- | --- |
| var stan::agrad::log\_determinant\_ldlt | ( | stan::math::LDLT\_factor< var, R, C > & | *A* | ) |  |

Definition at line 48 of file log\_determinant\_ldlt.hpp.

template<int R, int C>

|  |  |  |  |  |  |
| --- | --- | --- | --- | --- | --- |
| var stan::agrad::log\_determinant\_spd | ( | const Eigen::Matrix< var, R, C > & | *m* | ) |  |

inline

Definition at line 116 of file log\_determinant\_spd.hpp.

template<typename T >

|  |  |  |  |
| --- | --- | --- | --- |
| fvar<T> stan::agrad::log\_diff\_exp | ( | const fvar< T > & | *x1*, |
|  |  | const fvar< T > & | *x2* |
|  | ) |  |  |

inline

Definition at line 14 of file log\_diff\_exp.hpp.

template<typename T1 , typename T2 >

|  |  |  |  |
| --- | --- | --- | --- |
| fvar<T2> stan::agrad::log\_diff\_exp | ( | const T1 & | *x1*, |
|  |  | const fvar< T2 > & | *x2* |
|  | ) |  |  |

inline

Definition at line 25 of file log\_diff\_exp.hpp.

template<typename T1 , typename T2 >

|  |  |  |  |
| --- | --- | --- | --- |
| fvar<T1> stan::agrad::log\_diff\_exp | ( | const fvar< T1 > & | *x1*, |
|  |  | const T2 & | *x2* |
|  | ) |  |  |

inline

Definition at line 36 of file log\_diff\_exp.hpp.

|  |  |  |  |
| --- | --- | --- | --- |
| var stan::agrad::log\_diff\_exp | ( | const stan::agrad::var & | *a*, |
|  |  | const stan::agrad::var & | *b* |
|  | ) |  |  |

inline

Returns the log sum of exponentials.

Definition at line 55 of file log\_diff\_exp.hpp.

|  |  |  |  |
| --- | --- | --- | --- |
| var stan::agrad::log\_diff\_exp | ( | const stan::agrad::var & | *a*, |
|  |  | const double & | *b* |
|  | ) |  |  |

inline

Returns the log sum of exponentials.

Definition at line 62 of file log\_diff\_exp.hpp.

|  |  |  |  |
| --- | --- | --- | --- |
| var stan::agrad::log\_diff\_exp | ( | const double & | *a*, |
|  |  | const stan::agrad::var & | *b* |
|  | ) |  |  |

inline

Returns the log sum of exponentials.

Definition at line 69 of file log\_diff\_exp.hpp.

template<typename T >

|  |  |  |  |
| --- | --- | --- | --- |
| fvar<T> stan::agrad::log\_falling\_factorial | ( | const fvar< T > & | *x*, |
|  |  | const fvar< T > & | *n* |
|  | ) |  |  |

inline

Definition at line 15 of file log\_falling\_factorial.hpp.

template<typename T >

|  |  |  |  |
| --- | --- | --- | --- |
| fvar<T> stan::agrad::log\_falling\_factorial | ( | const double | *x*, |
|  |  | const fvar< T > & | *n* |
|  | ) |  |  |

inline

Definition at line 25 of file log\_falling\_factorial.hpp.

template<typename T >

|  |  |  |  |
| --- | --- | --- | --- |
| fvar<T> stan::agrad::log\_falling\_factorial | ( | const fvar< T > & | *x*, |
|  |  | const double | *n* |
|  | ) |  |  |

inline

Definition at line 35 of file log\_falling\_factorial.hpp.

|  |  |  |  |
| --- | --- | --- | --- |
| var stan::agrad::log\_falling\_factorial | ( | const var & | *a*, |
|  |  | const double & | *b* |
|  | ) |  |  |

inline

Definition at line 63 of file log\_falling\_factorial.hpp.

|  |  |  |  |
| --- | --- | --- | --- |
| var stan::agrad::log\_falling\_factorial | ( | const var & | *a*, |
|  |  | const var & | *b* |
|  | ) |  |  |

inline

Definition at line 68 of file log\_falling\_factorial.hpp.

|  |  |  |  |
| --- | --- | --- | --- |
| var stan::agrad::log\_falling\_factorial | ( | const double & | *a*, |
|  |  | const var & | *b* |
|  | ) |  |  |

inline

Definition at line 73 of file log\_falling\_factorial.hpp.

template<typename T >

|  |  |  |  |  |  |
| --- | --- | --- | --- | --- | --- |
| fvar<T> stan::agrad::log\_inv\_logit | ( | const fvar< T > & | *x* | ) |  |

inline

Definition at line 15 of file log\_inv\_logit.hpp.

template<typename T >

|  |  |  |  |
| --- | --- | --- | --- |
| fvar<T> stan::agrad::log\_rising\_factorial | ( | const fvar< T > & | *x*, |
|  |  | const fvar< T > & | *n* |
|  | ) |  |  |

inline

Definition at line 16 of file log\_rising\_factorial.hpp.

template<typename T >

|  |  |  |  |
| --- | --- | --- | --- |
| fvar<T> stan::agrad::log\_rising\_factorial | ( | const fvar< T > & | *x*, |
|  |  | const double | *n* |
|  | ) |  |  |

inline

Definition at line 27 of file log\_rising\_factorial.hpp.

template<typename T >

|  |  |  |  |
| --- | --- | --- | --- |
| fvar<T> stan::agrad::log\_rising\_factorial | ( | const double | *x*, |
|  |  | const fvar< T > & | *n* |
|  | ) |  |  |

inline

Definition at line 39 of file log\_rising\_factorial.hpp.

|  |  |  |  |
| --- | --- | --- | --- |
| var stan::agrad::log\_rising\_factorial | ( | const var & | *a*, |
|  |  | const double & | *b* |
|  | ) |  |  |

inline

Definition at line 49 of file log\_rising\_factorial.hpp.

|  |  |  |  |
| --- | --- | --- | --- |
| var stan::agrad::log\_rising\_factorial | ( | const var & | *a*, |
|  |  | const var & | *b* |
|  | ) |  |  |

inline

Definition at line 54 of file log\_rising\_factorial.hpp.

|  |  |  |  |
| --- | --- | --- | --- |
| var stan::agrad::log\_rising\_factorial | ( | const double & | *a*, |
|  |  | const var & | *b* |
|  | ) |  |  |

inline

Definition at line 59 of file log\_rising\_factorial.hpp.

template<typename T >

|  |  |  |  |  |  |
| --- | --- | --- | --- | --- | --- |
| Eigen::Matrix<fvar<T>,Eigen::Dynamic,1> stan::agrad::log\_softmax | ( | const Eigen::Matrix< fvar< T >, Eigen::Dynamic, 1 > & | *alpha* | ) |  |

inline

Definition at line 16 of file log\_softmax.hpp.

|  |  |  |  |  |  |
| --- | --- | --- | --- | --- | --- |
| Eigen::Matrix<var,Eigen::Dynamic,1> stan::agrad::log\_softmax | ( | const Eigen::Matrix< var, Eigen::Dynamic, 1 > & | *alpha* | ) |  |

inline

Return the softmax of the specified Eigen vector.

Softmax is guaranteed to return a simplex.

The gradient calculations are unfolded.

Parameters
:   |  |  |
    | --- | --- |
    | alpha | Unconstrained input vector. |

Returns
:   Softmax of the input.

Exceptions
:   |  |  |
    | --- | --- |
    | std::domain\_error | If the input vector is size 0. |

Definition at line 60 of file log\_softmax.hpp.

template<typename T >

|  |  |  |  |
| --- | --- | --- | --- |
| fvar<T> stan::agrad::log\_sum\_exp | ( | const fvar< T > & | *x1*, |
|  |  | const fvar< T > & | *x2* |
|  | ) |  |  |

inline

Definition at line 15 of file log\_sum\_exp.hpp.

template<typename T , int R, int C>

|  |  |  |  |  |  |
| --- | --- | --- | --- | --- | --- |
| fvar<T> stan::agrad::log\_sum\_exp | ( | const Eigen::Matrix< fvar< T >, R, C > & | *v* | ) |  |

Definition at line 19 of file log\_sum\_exp.hpp.

template<typename T >

|  |  |  |  |
| --- | --- | --- | --- |
| fvar<T> stan::agrad::log\_sum\_exp | ( | const double | *x1*, |
|  |  | const fvar< T > & | *x2* |
|  | ) |  |  |

inline

Definition at line 26 of file log\_sum\_exp.hpp.

template<typename T >

|  |  |  |  |
| --- | --- | --- | --- |
| fvar<T> stan::agrad::log\_sum\_exp | ( | const fvar< T > & | *x1*, |
|  |  | const double | *x2* |
|  | ) |  |  |

inline

Definition at line 36 of file log\_sum\_exp.hpp.

template<typename T >

|  |  |  |  |  |  |
| --- | --- | --- | --- | --- | --- |
| fvar<T> stan::agrad::log\_sum\_exp | ( | const std::vector< fvar< T > > & | *v* | ) |  |

Definition at line 45 of file log\_sum\_exp.hpp.

template<int R, int C>

|  |  |  |  |  |  |
| --- | --- | --- | --- | --- | --- |
| var stan::agrad::log\_sum\_exp | ( | const Eigen::Matrix< var, R, C > & | *x* | ) |  |

inline

Returns the log sum of exponentials.

Parameters
:   |  |  |
    | --- | --- |
    | x | matrix |

Definition at line 59 of file log\_sum\_exp.hpp.

|  |  |  |  |
| --- | --- | --- | --- |
| var stan::agrad::log\_sum\_exp | ( | const stan::agrad::var & | *a*, |
|  |  | const stan::agrad::var & | *b* |
|  | ) |  |  |

inline

Returns the log sum of exponentials.

Definition at line 81 of file log\_sum\_exp.hpp.

|  |  |  |  |
| --- | --- | --- | --- |
| var stan::agrad::log\_sum\_exp | ( | const stan::agrad::var & | *a*, |
|  |  | const double & | *b* |
|  | ) |  |  |

inline

Returns the log sum of exponentials.

Definition at line 88 of file log\_sum\_exp.hpp.

|  |  |  |  |
| --- | --- | --- | --- |
| var stan::agrad::log\_sum\_exp | ( | const double & | *a*, |
|  |  | const stan::agrad::var & | *b* |
|  | ) |  |  |

inline

Returns the log sum of exponentials.

Definition at line 95 of file log\_sum\_exp.hpp.

|  |  |  |  |  |  |
| --- | --- | --- | --- | --- | --- |
| var stan::agrad::log\_sum\_exp | ( | const std::vector< var > & | *x* | ) |  |

inline

Returns the log sum of exponentials.

Definition at line 102 of file log\_sum\_exp.hpp.

template<typename T >

|  |  |  |  |  |  |
| --- | --- | --- | --- | --- | --- |
| fvar<T> stan::agrad::logit | ( | const fvar< T > & | *x* | ) |  |

inline

Definition at line 17 of file logit.hpp.

template<typename T , int R1, int C1, int R2, int C2>

|  |  |  |  |
| --- | --- | --- | --- |
| Eigen::Matrix<fvar<T>,R1,C2> stan::agrad::mdivide\_left | ( | const Eigen::Matrix< fvar< T >, R1, C1 > & | *A*, |
|  |  | const Eigen::Matrix< fvar< T >, R2, C2 > & | *b* |
|  | ) |  |  |

inline

Definition at line 25 of file mdivide\_left.hpp.

template<typename T , int R1, int C1, int R2, int C2>

|  |  |  |  |
| --- | --- | --- | --- |
| Eigen::Matrix<fvar<T>,R1,C2> stan::agrad::mdivide\_left | ( | const Eigen::Matrix< double, R1, C1 > & | *A*, |
|  |  | const Eigen::Matrix< fvar< T >, R2, C2 > & | *b* |
|  | ) |  |  |

inline

Definition at line 69 of file mdivide\_left.hpp.

template<typename T , int R1, int C1, int R2, int C2>

|  |  |  |  |
| --- | --- | --- | --- |
| Eigen::Matrix<fvar<T>,R1,C2> stan::agrad::mdivide\_left | ( | const Eigen::Matrix< fvar< T >, R1, C1 > & | *A*, |
|  |  | const Eigen::Matrix< double, R2, C2 > & | *b* |
|  | ) |  |  |

inline

Definition at line 95 of file mdivide\_left.hpp.

template<int R1, int C1, int R2, int C2>

|  |  |  |  |
| --- | --- | --- | --- |
| Eigen::Matrix<var,R1,C2> stan::agrad::mdivide\_left | ( | const Eigen::Matrix< var, R1, C1 > & | *A*, |
|  |  | const Eigen::Matrix< var, R2, C2 > & | *b* |
|  | ) |  |  |

inline

Definition at line 264 of file mdivide\_left.hpp.

template<int R1, int C1, int R2, int C2>

|  |  |  |  |
| --- | --- | --- | --- |
| Eigen::Matrix<var,R1,C2> stan::agrad::mdivide\_left | ( | const Eigen::Matrix< var, R1, C1 > & | *A*, |
|  |  | const Eigen::Matrix< double, R2, C2 > & | *b* |
|  | ) |  |  |

inline

Definition at line 288 of file mdivide\_left.hpp.

template<int R1, int C1, int R2, int C2>

|  |  |  |  |
| --- | --- | --- | --- |
| Eigen::Matrix<var,R1,C2> stan::agrad::mdivide\_left | ( | const Eigen::Matrix< double, R1, C1 > & | *A*, |
|  |  | const Eigen::Matrix< var, R2, C2 > & | *b* |
|  | ) |  |  |

inline

Definition at line 312 of file mdivide\_left.hpp.

template<int R1, int C1, int R2, int C2, typename T2 >

|  |  |  |  |
| --- | --- | --- | --- |
| Eigen::Matrix<fvar<T2>,R1,C2> stan::agrad::mdivide\_left\_ldlt | ( | const stan::math::LDLT\_factor< double, R1, C1 > & | *A*, |
|  |  | const Eigen::Matrix< fvar< T2 >, R2, C2 > & | *b* |
|  | ) |  |  |

inline

Returns the solution of the system Ax=b given an LDLT\_factor of A.

Parameters
:   |  |  |
    | --- | --- |
    | A | LDLT\_factor |
    | b | Right hand side matrix or vector. |

Returns
:   x = b A^-1, solution of the linear system.

Exceptions
:   |  |  |
    | --- | --- |
    | std::domain\_error | if rows of b don't match the size of A. |

Definition at line 25 of file mdivide\_left\_ldlt.hpp.

template<int R1, int C1, int R2, int C2>

|  |  |  |  |
| --- | --- | --- | --- |
| Eigen::Matrix<var,R1,C2> stan::agrad::mdivide\_left\_ldlt | ( | const stan::math::LDLT\_factor< var, R1, C1 > & | *A*, |
|  |  | const Eigen::Matrix< var, R2, C2 > & | *b* |
|  | ) |  |  |

inline

Returns the solution of the system Ax=b given an LDLT\_factor of A.

Parameters
:   |  |  |
    | --- | --- |
    | A | LDLT\_factor |
    | b | Right hand side matrix or vector. |

Returns
:   x = b A^-1, solution of the linear system.

Exceptions
:   |  |  |
    | --- | --- |
    | std::domain\_error | if rows of b don't match the size of A. |

Definition at line 243 of file mdivide\_left\_ldlt.hpp.

template<int R1, int C1, int R2, int C2>

|  |  |  |  |
| --- | --- | --- | --- |
| Eigen::Matrix<var,R1,C2> stan::agrad::mdivide\_left\_ldlt | ( | const stan::math::LDLT\_factor< var, R1, C1 > & | *A*, |
|  |  | const Eigen::Matrix< double, R2, C2 > & | *b* |
|  | ) |  |  |

inline

Returns the solution of the system Ax=b given an LDLT\_factor of A.

Parameters
:   |  |  |
    | --- | --- |
    | A | LDLT\_factor |
    | b | Right hand side matrix or vector. |

Returns
:   x = b A^-1, solution of the linear system.

Exceptions
:   |  |  |
    | --- | --- |
    | std::domain\_error | if rows of b don't match the size of A. |

Definition at line 269 of file mdivide\_left\_ldlt.hpp.

template<int R1, int C1, int R2, int C2>

|  |  |  |  |
| --- | --- | --- | --- |
| Eigen::Matrix<var,R1,C2> stan::agrad::mdivide\_left\_ldlt | ( | const stan::math::LDLT\_factor< double, R1, C1 > & | *A*, |
|  |  | const Eigen::Matrix< var, R2, C2 > & | *b* |
|  | ) |  |  |

inline

Returns the solution of the system Ax=b given an LDLT\_factor of A.

Parameters
:   |  |  |
    | --- | --- |
    | A | LDLT\_factor |
    | b | Right hand side matrix or vector. |

Returns
:   x = b A^-1, solution of the linear system.

Exceptions
:   |  |  |
    | --- | --- |
    | std::domain\_error | if rows of b don't match the size of A. |

Definition at line 295 of file mdivide\_left\_ldlt.hpp.

template<int R1, int C1, int R2, int C2>

|  |  |  |  |
| --- | --- | --- | --- |
| Eigen::Matrix<var,R1,C2> stan::agrad::mdivide\_left\_spd | ( | const Eigen::Matrix< var, R1, C1 > & | *A*, |
|  |  | const Eigen::Matrix< var, R2, C2 > & | *b* |
|  | ) |  |  |

inline

Definition at line 244 of file mdivide\_left\_spd.hpp.

template<int R1, int C1, int R2, int C2>

|  |  |  |  |
| --- | --- | --- | --- |
| Eigen::Matrix<var,R1,C2> stan::agrad::mdivide\_left\_spd | ( | const Eigen::Matrix< var, R1, C1 > & | *A*, |
|  |  | const Eigen::Matrix< double, R2, C2 > & | *b* |
|  | ) |  |  |

inline

Definition at line 268 of file mdivide\_left\_spd.hpp.

template<int R1, int C1, int R2, int C2>

|  |  |  |  |
| --- | --- | --- | --- |
| Eigen::Matrix<var,R1,C2> stan::agrad::mdivide\_left\_spd | ( | const Eigen::Matrix< double, R1, C1 > & | *A*, |
|  |  | const Eigen::Matrix< var, R2, C2 > & | *b* |
|  | ) |  |  |

inline

Definition at line 292 of file mdivide\_left\_spd.hpp.

template<int TriView, int R1, int C1, int R2, int C2>

|  |  |  |  |
| --- | --- | --- | --- |
| Eigen::Matrix<var,R1,C2> stan::agrad::mdivide\_left\_tri | ( | const Eigen::Matrix< var, R1, C1 > & | *A*, |
|  |  | const Eigen::Matrix< var, R2, C2 > & | *b* |
|  | ) |  |  |

inline

Definition at line 296 of file mdivide\_left\_tri.hpp.

template<int TriView, int R1, int C1, int R2, int C2>

|  |  |  |  |
| --- | --- | --- | --- |
| Eigen::Matrix<var,R1,C2> stan::agrad::mdivide\_left\_tri | ( | const Eigen::Matrix< double, R1, C1 > & | *A*, |
|  |  | const Eigen::Matrix< var, R2, C2 > & | *b* |
|  | ) |  |  |

inline

Definition at line 319 of file mdivide\_left\_tri.hpp.

template<int TriView, int R1, int C1, int R2, int C2>

|  |  |  |  |
| --- | --- | --- | --- |
| Eigen::Matrix<var,R1,C2> stan::agrad::mdivide\_left\_tri | ( | const Eigen::Matrix< var, R1, C1 > & | *A*, |
|  |  | const Eigen::Matrix< double, R2, C2 > & | *b* |
|  | ) |  |  |

inline

Definition at line 342 of file mdivide\_left\_tri.hpp.

template<typename T , int R1, int C1, int R2, int C2>

|  |  |  |  |
| --- | --- | --- | --- |
| Eigen::Matrix<fvar<T>,R1,C1> stan::agrad::mdivide\_left\_tri\_low | ( | const Eigen::Matrix< fvar< T >, R1, C1 > & | *A*, |
|  |  | const Eigen::Matrix< fvar< T >, R2, C2 > & | *b* |
|  | ) |  |  |

inline

Definition at line 22 of file mdivide\_left\_tri\_low.hpp.

template<typename T , int R1, int C1, int R2, int C2>

|  |  |  |  |
| --- | --- | --- | --- |
| Eigen::Matrix<fvar<T>,R1,C1> stan::agrad::mdivide\_left\_tri\_low | ( | const Eigen::Matrix< double, R1, C1 > & | *A*, |
|  |  | const Eigen::Matrix< fvar< T >, R2, C2 > & | *b* |
|  | ) |  |  |

inline

Definition at line 67 of file mdivide\_left\_tri\_low.hpp.

template<typename T , int R1, int C1, int R2, int C2>

|  |  |  |  |
| --- | --- | --- | --- |
| Eigen::Matrix<fvar<T>,R1,C1> stan::agrad::mdivide\_left\_tri\_low | ( | const Eigen::Matrix< fvar< T >, R1, C1 > & | *A*, |
|  |  | const Eigen::Matrix< double, R2, C2 > & | *b* |
|  | ) |  |  |

inline

Definition at line 107 of file mdivide\_left\_tri\_low.hpp.

template<typename T , int R1, int C1, int R2, int C2>

|  |  |  |  |
| --- | --- | --- | --- |
| Eigen::Matrix<fvar<T>,R1,C2> stan::agrad::mdivide\_right | ( | const Eigen::Matrix< fvar< T >, R1, C1 > & | *A*, |
|  |  | const Eigen::Matrix< fvar< T >, R2, C2 > & | *b* |
|  | ) |  |  |

inline

Definition at line 26 of file mdivide\_right.hpp.

template<typename T , int R1, int C1, int R2, int C2>

|  |  |  |  |
| --- | --- | --- | --- |
| Eigen::Matrix<fvar<T>,R1,C2> stan::agrad::mdivide\_right | ( | const Eigen::Matrix< fvar< T >, R1, C1 > & | *A*, |
|  |  | const Eigen::Matrix< double, R2, C2 > & | *b* |
|  | ) |  |  |

inline

Definition at line 70 of file mdivide\_right.hpp.

template<typename T , int R1, int C1, int R2, int C2>

|  |  |  |  |
| --- | --- | --- | --- |
| Eigen::Matrix<fvar<T>,R1,C2> stan::agrad::mdivide\_right | ( | const Eigen::Matrix< double, R1, C1 > & | *A*, |
|  |  | const Eigen::Matrix< fvar< T >, R2, C2 > & | *b* |
|  | ) |  |  |

inline

Definition at line 97 of file mdivide\_right.hpp.

template<typename T , int R1, int C1, int R2, int C2>

|  |  |  |  |
| --- | --- | --- | --- |
| Eigen::Matrix<fvar<T>,R1,C1> stan::agrad::mdivide\_right\_tri\_low | ( | const Eigen::Matrix< fvar< T >, R1, C1 > & | *A*, |
|  |  | const Eigen::Matrix< fvar< T >, R2, C2 > & | *b* |
|  | ) |  |  |

inline

Definition at line 22 of file mdivide\_right\_tri\_low.hpp.

template<typename T , int R1, int C1, int R2, int C2>

|  |  |  |  |
| --- | --- | --- | --- |
| Eigen::Matrix<fvar<T>,R1,C2> stan::agrad::mdivide\_right\_tri\_low | ( | const Eigen::Matrix< fvar< T >, R1, C1 > & | *A*, |
|  |  | const Eigen::Matrix< double, R2, C2 > & | *b* |
|  | ) |  |  |

inline

Definition at line 67 of file mdivide\_right\_tri\_low.hpp.

template<typename T , int R1, int C1, int R2, int C2>

|  |  |  |  |
| --- | --- | --- | --- |
| Eigen::Matrix<fvar<T>,R1,C2> stan::agrad::mdivide\_right\_tri\_low | ( | const Eigen::Matrix< double, R1, C1 > & | *A*, |
|  |  | const Eigen::Matrix< fvar< T >, R2, C2 > & | *b* |
|  | ) |  |  |

inline

Definition at line 102 of file mdivide\_right\_tri\_low.hpp.

template<typename T >

|  |  |  |  |
| --- | --- | --- | --- |
| fvar<T> stan::agrad::modified\_bessel\_first\_kind | ( | int | *v*, |
|  |  | const fvar< T > & | *z* |
|  | ) |  |  |

inline

Definition at line 15 of file modified\_bessel\_first\_kind.hpp.

|  |  |  |  |
| --- | --- | --- | --- |
| var stan::agrad::modified\_bessel\_first\_kind | ( | const int & | *v*, |
|  |  | const var & | *a* |
|  | ) |  |  |

inline

Definition at line 24 of file modified\_bessel\_first\_kind.hpp.

template<typename T >

|  |  |  |  |
| --- | --- | --- | --- |
| fvar<T> stan::agrad::modified\_bessel\_second\_kind | ( | int | *v*, |
|  |  | const fvar< T > & | *z* |
|  | ) |  |  |

inline

Definition at line 15 of file modified\_bessel\_second\_kind.hpp.

|  |  |  |  |
| --- | --- | --- | --- |
| var stan::agrad::modified\_bessel\_second\_kind | ( | const int & | *v*, |
|  |  | const var & | *a* |
|  | ) |  |  |

inline

Definition at line 24 of file modified\_bessel\_second\_kind.hpp.

template<typename T , int R1, int C1>

|  |  |  |  |
| --- | --- | --- | --- |
| Eigen::Matrix<fvar<T>,R1,C1> stan::agrad::multiply | ( | const Eigen::Matrix< fvar< T >, R1, C1 > & | *m*, |
|  |  | const fvar< T > & | *c* |
|  | ) |  |  |

inline

Definition at line 21 of file multiply.hpp.

template<typename T1 , typename T2 >

|  |  |  |  |
| --- | --- | --- | --- |
| boost::enable\_if\_c< (boost::is\_scalar<T1>::value \|\| boost::is\_same<T1,var>::value) && (boost::is\_scalar<T2>::value \|\| boost::is\_same<T2,var>::value), typename boost::math::tools::promote\_args<T1,T2>::type>::type stan::agrad::multiply | ( | const T1 & | *v*, |
|  |  | const T2 & | *c* |
|  | ) |  |  |

inline

Return the product of two scalars.

Parameters
:   |  |  |  |
    | --- | --- | --- |
    | [in] | v | First scalar. |
    | [in] | c | Specified scalar. |

Returns
:   Product of scalars.

Definition at line 31 of file multiply.hpp.

template<typename T , int R2, int C2>

|  |  |  |  |
| --- | --- | --- | --- |
| Eigen::Matrix<fvar<T>, R2, C2> stan::agrad::multiply | ( | const Eigen::Matrix< fvar< T >, R2, C2 > & | *m*, |
|  |  | const double | *c* |
|  | ) |  |  |

inline

Definition at line 33 of file multiply.hpp.

template<typename T1 , typename T2 , int R2, int C2>

|  |  |  |  |
| --- | --- | --- | --- |
| Eigen::Matrix<var,R2,C2> stan::agrad::multiply | ( | const T1 & | *c*, |
|  |  | const Eigen::Matrix< T2, R2, C2 > & | *m* |
|  | ) |  |  |

inline

Return the product of scalar and matrix.

Parameters
:   |  |  |  |
    | --- | --- | --- |
    | [in] | c | Specified scalar. |
    | [in] | m | Matrix. |

Returns
:   Product of scalar and matrix.

Definition at line 42 of file multiply.hpp.

template<typename T , int R1, int C1>

|  |  |  |  |
| --- | --- | --- | --- |
| Eigen::Matrix<fvar<T>,R1,C1> stan::agrad::multiply | ( | const Eigen::Matrix< double, R1, C1 > & | *m*, |
|  |  | const fvar< T > & | *c* |
|  | ) |  |  |

inline

Definition at line 45 of file multiply.hpp.

template<typename T1 , int R1, int C1, typename T2 >

|  |  |  |  |
| --- | --- | --- | --- |
| Eigen::Matrix<var,R1,C1> stan::agrad::multiply | ( | const Eigen::Matrix< T1, R1, C1 > & | *m*, |
|  |  | const T2 & | *c* |
|  | ) |  |  |

inline

Return the product of scalar and matrix.

Parameters
:   |  |  |  |
    | --- | --- | --- |
    | [in] | m | Matrix. |
    | [in] | c | Specified scalar. |

Returns
:   Product of scalar and matrix.

Definition at line 56 of file multiply.hpp.

template<typename T , int R1, int C1>

|  |  |  |  |
| --- | --- | --- | --- |
| Eigen::Matrix<fvar<T>,R1,C1> stan::agrad::multiply | ( | const fvar< T > & | *c*, |
|  |  | const Eigen::Matrix< fvar< T >, R1, C1 > & | *m* |
|  | ) |  |  |

inline

Definition at line 57 of file multiply.hpp.

template<typename T , int R1, int C1>

|  |  |  |  |
| --- | --- | --- | --- |
| Eigen::Matrix<fvar<T>,R1,C1> stan::agrad::multiply | ( | const double | *c*, |
|  |  | const Eigen::Matrix< fvar< T >, R1, C1 > & | *m* |
|  | ) |  |  |

inline

Definition at line 64 of file multiply.hpp.

template<typename T , int R1, int C1>

|  |  |  |  |
| --- | --- | --- | --- |
| Eigen::Matrix<fvar<T>,R1,C1> stan::agrad::multiply | ( | const fvar< T > & | *c*, |
|  |  | const Eigen::Matrix< double, R1, C1 > & | *m* |
|  | ) |  |  |

inline

Definition at line 71 of file multiply.hpp.

template<typename T1 , int R1, int C1, typename T2 , int R2, int C2>

|  |  |  |  |
| --- | --- | --- | --- |
| boost::enable\_if\_c< boost::is\_same<T1,var>::value \|\| boost::is\_same<T2,var>::value, Eigen::Matrix<var,R1,C2> >::type stan::agrad::multiply | ( | const Eigen::Matrix< T1, R1, C1 > & | *m1*, |
|  |  | const Eigen::Matrix< T2, R2, C2 > & | *m2* |
|  | ) |  |  |

inline

Return the product of the specified matrices.

The number of columns in the first matrix must be the same as the number of rows in the second matrix.

Parameters
:   |  |  |  |
    | --- | --- | --- |
    | [in] | m1 | First matrix. |
    | [in] | m2 | Second matrix. |

Returns
:   The product of the first and second matrices.

Exceptions
:   |  |  |
    | --- | --- |
    | std::domain\_error | if the number of columns of m1 does not match the number of rows of m2. |

Definition at line 76 of file multiply.hpp.

template<typename T , int R1, int C1, int R2, int C2>

|  |  |  |  |
| --- | --- | --- | --- |
| Eigen::Matrix<fvar<T>,R1,C2> stan::agrad::multiply | ( | const Eigen::Matrix< fvar< T >, R1, C1 > & | *m1*, |
|  |  | const Eigen::Matrix< fvar< T >, R2, C2 > & | *m2* |
|  | ) |  |  |

inline

Definition at line 78 of file multiply.hpp.

template<typename T , int R1, int C1, int R2, int C2>

|  |  |  |  |
| --- | --- | --- | --- |
| Eigen::Matrix<fvar<T>,R1,C2> stan::agrad::multiply | ( | const Eigen::Matrix< fvar< T >, R1, C1 > & | *m1*, |
|  |  | const Eigen::Matrix< double, R2, C2 > & | *m2* |
|  | ) |  |  |

inline

Definition at line 96 of file multiply.hpp.

template<typename T , int R1, int C1, int R2, int C2>

|  |  |  |  |
| --- | --- | --- | --- |
| Eigen::Matrix<fvar<T>,R1,C2> stan::agrad::multiply | ( | const Eigen::Matrix< double, R1, C1 > & | *m1*, |
|  |  | const Eigen::Matrix< fvar< T >, R2, C2 > & | *m2* |
|  | ) |  |  |

inline

Definition at line 114 of file multiply.hpp.

template<typename T1 , int C1, typename T2 , int R2>

|  |  |  |  |
| --- | --- | --- | --- |
| boost::enable\_if\_c< boost::is\_same<T1,var>::value \|\| boost::is\_same<T2,var>::value, var >::type stan::agrad::multiply | ( | const Eigen::Matrix< T1, 1, C1 > & | *rv*, |
|  |  | const Eigen::Matrix< T2, R2, 1 > & | *v* |
|  | ) |  |  |

inline

Return the scalar product of the specified row vector and specified column vector.

The return is the same as the dot product. The two vectors must be the same size.

Parameters
:   |  |  |  |
    | --- | --- | --- |
    | [in] | rv | Row vector. |
    | [in] | v | Column vector. |

Returns
:   Scalar result of multiplying row vector by column vector.

Exceptions
:   |  |  |
    | --- | --- |
    | std::domain\_error | if rv and v are not the same size |

Definition at line 123 of file multiply.hpp.

template<typename T , int C1, int R2>

|  |  |  |  |
| --- | --- | --- | --- |
| fvar<T> stan::agrad::multiply | ( | const Eigen::Matrix< fvar< T >, 1, C1 > & | *rv*, |
|  |  | const Eigen::Matrix< fvar< T >, R2, 1 > & | *v* |
|  | ) |  |  |

inline

Definition at line 132 of file multiply.hpp.

template<typename T , int C1, int R2>

|  |  |  |  |
| --- | --- | --- | --- |
| fvar<T> stan::agrad::multiply | ( | const Eigen::Matrix< fvar< T >, 1, C1 > & | *rv*, |
|  |  | const Eigen::Matrix< double, R2, 1 > & | *v* |
|  | ) |  |  |

inline

Definition at line 142 of file multiply.hpp.

template<typename T , int C1, int R2>

|  |  |  |  |
| --- | --- | --- | --- |
| fvar<T> stan::agrad::multiply | ( | const Eigen::Matrix< double, 1, C1 > & | *rv*, |
|  |  | const Eigen::Matrix< fvar< T >, R2, 1 > & | *v* |
|  | ) |  |  |

inline

Definition at line 152 of file multiply.hpp.

template<typename T >

|  |  |  |  |
| --- | --- | --- | --- |
| fvar<T> stan::agrad::multiply\_log | ( | const fvar< T > & | *x1*, |
|  |  | const fvar< T > & | *x2* |
|  | ) |  |  |

inline

Definition at line 15 of file multiply\_log.hpp.

template<typename T >

|  |  |  |  |
| --- | --- | --- | --- |
| fvar<T> stan::agrad::multiply\_log | ( | const double | *x1*, |
|  |  | const fvar< T > & | *x2* |
|  | ) |  |  |

inline

Definition at line 25 of file multiply\_log.hpp.

template<typename T >

|  |  |  |  |
| --- | --- | --- | --- |
| fvar<T> stan::agrad::multiply\_log | ( | const fvar< T > & | *x1*, |
|  |  | const double | *x2* |
|  | ) |  |  |

inline

Definition at line 35 of file multiply\_log.hpp.

|  |  |  |  |
| --- | --- | --- | --- |
| var stan::agrad::multiply\_log | ( | const var & | *a*, |
|  |  | const var & | *b* |
|  | ) |  |  |

inline

Return the value of a\*log(b).

When both a and b are 0, the value returned is 0. The partial deriviative with respect to a is log(b). The partial deriviative with respect to b is a/b. When a and b are both 0, this is set to Inf.

Parameters
:   |  |  |
    | --- | --- |
    | a | First variable. |
    | b | Second variable. |

Returns
:   Value of a\*log(b)

Definition at line 77 of file multiply\_log.hpp.

|  |  |  |  |
| --- | --- | --- | --- |
| var stan::agrad::multiply\_log | ( | const var & | *a*, |
|  |  | const double | *b* |
|  | ) |  |  |

inline

Return the value of a\*log(b).

When both a and b are 0, the value returned is 0. The partial deriviative with respect to a is log(b).

Parameters
:   |  |  |
    | --- | --- |
    | a | First variable. |
    | b | Second scalar. |

Returns
:   Value of a\*log(b)

Definition at line 90 of file multiply\_log.hpp.

|  |  |  |  |
| --- | --- | --- | --- |
| var stan::agrad::multiply\_log | ( | const double | *a*, |
|  |  | const var & | *b* |
|  | ) |  |  |

inline

Return the value of a\*log(b).

When both a and b are 0, the value returned is 0. The partial deriviative with respect to b is a/b. When a and b are both 0, this is set to Inf.

Parameters
:   |  |  |
    | --- | --- |
    | a | First scalar. |
    | b | Second variable. |

Returns
:   Value of a\*log(b)

Definition at line 104 of file multiply\_log.hpp.

template<typename T , int R, int C>

|  |  |  |  |  |  |
| --- | --- | --- | --- | --- | --- |
| Eigen::Matrix<fvar<T>,R,R> stan::agrad::multiply\_lower\_tri\_self\_transpose | ( | const Eigen::Matrix< fvar< T >, R, C > & | *m* | ) |  |

inline

Definition at line 17 of file multiply\_lower\_tri\_self\_transpose.hpp.

|  |  |  |  |  |  |
| --- | --- | --- | --- | --- | --- |
| matrix\_v stan::agrad::multiply\_lower\_tri\_self\_transpose | ( | const matrix\_v & | *L* | ) |  |

inline

Definition at line 19 of file multiply\_lower\_tri\_self\_transpose.hpp.

|  |  |  |  |  |
| --- | --- | --- | --- | --- |
| static size\_t stan::agrad::nested\_size | ( |  | ) |  |

inlinestatic

Definition at line 103 of file var\_stack.hpp.

|  |  |  |  |  |  |
| --- | --- | --- | --- | --- | --- |
| bool stan::agrad::operator! | ( | const var & | *a* | ) |  |

inline

Prefix logical negation for the value of variables (C++).

The expression (!a) is equivalent to negating the scalar value of the variable a.

Note that this is the only logical operator defined for variables. Overridden logical conjunction (&&) and disjunction (||) operators do not apply the same "short circuit" rules as the built-in logical operators.

Parameters
:   |  |  |
    | --- | --- |
    | a | Variable to negate. |

Returns
:   True if variable is non-zero.

Definition at line 31 of file operator\_unary\_not.hpp.

template<typename T >

|  |  |  |  |
| --- | --- | --- | --- |
| bool stan::agrad::operator!= | ( | const fvar< T > & | *x*, |
|  |  | const fvar< T > & | *y* |
|  | ) |  |  |

inline

Definition at line 14 of file operator\_not\_equal.hpp.

template<typename T >

|  |  |  |  |
| --- | --- | --- | --- |
| bool stan::agrad::operator!= | ( | const fvar< T > & | *x*, |
|  |  | double | *y* |
|  | ) |  |  |

inline

Definition at line 21 of file operator\_not\_equal.hpp.

|  |  |  |  |
| --- | --- | --- | --- |
| bool stan::agrad::operator!= | ( | const var & | *a*, |
|  |  | const var & | *b* |
|  | ) |  |  |

inline

Inequality operator comparing two variables' values (C++).

Parameters
:   |  |  |
    | --- | --- |
    | a | First variable. |
    | b | Second variable. |

Returns
:   True if the first variable's value is not the same as the second's.

Definition at line 26 of file operator\_not\_equal.hpp.

template<typename T >

|  |  |  |  |
| --- | --- | --- | --- |
| bool stan::agrad::operator!= | ( | double | *x*, |
|  |  | const fvar< T > & | *y* |
|  | ) |  |  |

inline

Definition at line 28 of file operator\_not\_equal.hpp.

|  |  |  |  |
| --- | --- | --- | --- |
| bool stan::agrad::operator!= | ( | const var & | *a*, |
|  |  | const double | *b* |
|  | ) |  |  |

inline

Inequality operator comparing a variable's value and a double (C++).

Parameters
:   |  |  |
    | --- | --- |
    | a | First variable. |
    | b | Second value. |

Returns
:   True if the first variable's value is not the same as the second value.

Definition at line 39 of file operator\_not\_equal.hpp.

|  |  |  |  |
| --- | --- | --- | --- |
| bool stan::agrad::operator!= | ( | const double | *a*, |
|  |  | const var & | *b* |
|  | ) |  |  |

inline

Inequality operator comparing a double and a variable's value (C++).

Parameters
:   |  |  |
    | --- | --- |
    | a | First value. |
    | b | Second variable. |

Returns
:   True if the first value is not the same as the second variable's value.

Definition at line 52 of file operator\_not\_equal.hpp.

template<typename T >

|  |  |  |  |
| --- | --- | --- | --- |
| fvar<T> stan::agrad::operator\* | ( | const fvar< T > & | *x1*, |
|  |  | const fvar< T > & | *x2* |
|  | ) |  |  |

inline

Definition at line 14 of file operator\_multiplication.hpp.

template<typename T >

|  |  |  |  |
| --- | --- | --- | --- |
| fvar<T> stan::agrad::operator\* | ( | const double | *x1*, |
|  |  | const fvar< T > & | *x2* |
|  | ) |  |  |

inline

Definition at line 22 of file operator\_multiplication.hpp.

template<typename T >

|  |  |  |  |
| --- | --- | --- | --- |
| fvar<T> stan::agrad::operator\* | ( | const fvar< T > & | *x1*, |
|  |  | const double | *x2* |
|  | ) |  |  |

inline

Definition at line 29 of file operator\_multiplication.hpp.

|  |  |  |  |
| --- | --- | --- | --- |
| var stan::agrad::operator\* | ( | const var & | *a*, |
|  |  | const var & | *b* |
|  | ) |  |  |

inline

Multiplication operator for two variables (C++).

The partial derivatives are

, and

.

Parameters
:   |  |  |
    | --- | --- |
    | a | First variable operand. |
    | b | Second variable operand. |

Returns
:   Variable result of multiplying operands.

Definition at line 82 of file operator\_multiplication.hpp.

|  |  |  |  |
| --- | --- | --- | --- |
| var stan::agrad::operator\* | ( | const var & | *a*, |
|  |  | const double | *b* |
|  | ) |  |  |

inline

Multiplication operator for a variable and a scalar (C++).

The partial derivative for the variable is

, and

Parameters
:   |  |  |
    | --- | --- |
    | a | Variable operand. |
    | b | Scalar operand. |

Returns
:   Variable result of multiplying operands.

Definition at line 97 of file operator\_multiplication.hpp.

|  |  |  |  |
| --- | --- | --- | --- |
| var stan::agrad::operator\* | ( | const double | *a*, |
|  |  | const var & | *b* |
|  | ) |  |  |

inline

Multiplication operator for a scalar and a variable (C++).

The partial derivative for the variable is

.

Parameters
:   |  |  |
    | --- | --- |
    | a | Scalar operand. |
    | b | Variable operand. |

Returns
:   Variable result of multiplying the operands.

Definition at line 114 of file operator\_multiplication.hpp.

template<typename T >

|  |  |  |  |
| --- | --- | --- | --- |
| fvar<T> stan::agrad::operator+ | ( | const fvar< T > & | *x1*, |
|  |  | const fvar< T > & | *x2* |
|  | ) |  |  |

inline

Definition at line 14 of file operator\_addition.hpp.

template<typename T >

|  |  |  |  |
| --- | --- | --- | --- |
| fvar<T> stan::agrad::operator+ | ( | const double | *x1*, |
|  |  | const fvar< T > & | *x2* |
|  | ) |  |  |

inline

Definition at line 21 of file operator\_addition.hpp.

template<typename T >

|  |  |  |  |
| --- | --- | --- | --- |
| fvar<T> stan::agrad::operator+ | ( | const fvar< T > & | *x1*, |
|  |  | const double | *x2* |
|  | ) |  |  |

inline

Definition at line 28 of file operator\_addition.hpp.

|  |  |  |  |  |  |
| --- | --- | --- | --- | --- | --- |
| var stan::agrad::operator+ | ( | const var & | *a* | ) |  |

inline

Unary plus operator for variables (C++).

The function simply returns its input, because

.

The effect of unary plus on a built-in C++ scalar type is integer promotion. Because variables are all double-precision floating point already, promotion is not necessary.

Parameters
:   |  |  |
    | --- | --- |
    | a | Argument variable. |

Returns
:   The input reference.

Definition at line 43 of file operator\_unary\_plus.hpp.

|  |  |  |  |
| --- | --- | --- | --- |
| var stan::agrad::operator+ | ( | const var & | *a*, |
|  |  | const var & | *b* |
|  | ) |  |  |

inline

Addition operator for variables (C++).

The partial derivatives are defined by

, and

.

Parameters
:   |  |  |
    | --- | --- |
    | a | First variable operand. |
    | b | Second variable operand. |

Returns
:   Variable result of adding two variables.

Definition at line 83 of file operator\_addition.hpp.

|  |  |  |  |
| --- | --- | --- | --- |
| var stan::agrad::operator+ | ( | const var & | *a*, |
|  |  | const double | *b* |
|  | ) |  |  |

inline

Addition operator for variable and scalar (C++).

The derivative with respect to the variable is

.

Parameters
:   |  |  |
    | --- | --- |
    | a | First variable operand. |
    | b | Second scalar operand. |

Returns
:   Result of adding variable and scalar.

Definition at line 98 of file operator\_addition.hpp.

|  |  |  |  |
| --- | --- | --- | --- |
| var stan::agrad::operator+ | ( | const double | *a*, |
|  |  | const var & | *b* |
|  | ) |  |  |

inline

Addition operator for scalar and variable (C++).

The derivative with respect to the variable is

.

Parameters
:   |  |  |
    | --- | --- |
    | a | First scalar operand. |
    | b | Second variable operand. |

Returns
:   Result of adding variable and scalar.

Definition at line 115 of file operator\_addition.hpp.

|  |  |  |  |  |  |
| --- | --- | --- | --- | --- | --- |
| var& stan::agrad::operator++ | ( | var & | *a* | ) |  |

inline

Prefix increment operator for variables (C++).

Following C++, (++a) is defined to behave exactly as (a = a + 1.0) does, but is faster and uses less memory. In particular, the result is an assignable lvalue.

Parameters
:   |  |  |
    | --- | --- |
    | a | Variable to increment. |

Returns
:   Reference the result of incrementing this input variable.

Definition at line 35 of file operator\_unary\_increment.hpp.

|  |  |  |  |
| --- | --- | --- | --- |
| var stan::agrad::operator++ | ( | var & | *a*, |
|  |  | int |  |
|  | ) |  |  |

inline

Postfix increment operator for variables (C++).

Following C++, the expression `(a++)` is defined to behave like the sequence of operations

`var temp = a; a = a + 1.0; return temp;`

Parameters
:   |  |  |
    | --- | --- |
    | a | Variable to increment. |

Returns
:   Input variable.

Definition at line 51 of file operator\_unary\_increment.hpp.

template<typename T >

|  |  |  |  |
| --- | --- | --- | --- |
| fvar<T> stan::agrad::operator- | ( | const fvar< T > & | *x1*, |
|  |  | const fvar< T > & | *x2* |
|  | ) |  |  |

inline

Definition at line 14 of file operator\_subtraction.hpp.

template<typename T >

|  |  |  |  |  |  |
| --- | --- | --- | --- | --- | --- |
| fvar<T> stan::agrad::operator- | ( | const fvar< T > & | *x* | ) |  |

inline

Definition at line 14 of file operator\_unary\_minus.hpp.

template<typename T >

|  |  |  |  |
| --- | --- | --- | --- |
| fvar<T> stan::agrad::operator- | ( | const double | *x1*, |
|  |  | const fvar< T > & | *x2* |
|  | ) |  |  |

inline

Definition at line 21 of file operator\_subtraction.hpp.

template<typename T >

|  |  |  |  |
| --- | --- | --- | --- |
| fvar<T> stan::agrad::operator- | ( | const fvar< T > & | *x1*, |
|  |  | const double | *x2* |
|  | ) |  |  |

inline

Definition at line 28 of file operator\_subtraction.hpp.

|  |  |  |  |  |  |
| --- | --- | --- | --- | --- | --- |
| var stan::agrad::operator- | ( | const var & | *a* | ) |  |

inline

Unary negation operator for variables (C++).

.

Parameters
:   |  |  |
    | --- | --- |
    | a | Argument variable. |

Returns
:   Negation of variable.

Definition at line 50 of file operator\_unary\_negative.hpp.

|  |  |  |  |
| --- | --- | --- | --- |
| var stan::agrad::operator- | ( | const var & | *a*, |
|  |  | const var & | *b* |
|  | ) |  |  |

inline

Subtraction operator for variables (C++).

The partial derivatives are defined by

, and

.

Parameters
:   |  |  |
    | --- | --- |
    | a | First variable operand. |
    | b | Second variable operand. |

Returns
:   Variable result of subtracting the second variable from the first.

Definition at line 98 of file operator\_subtraction.hpp.

|  |  |  |  |
| --- | --- | --- | --- |
| var stan::agrad::operator- | ( | const var & | *a*, |
|  |  | const double | *b* |
|  | ) |  |  |

inline

Subtraction operator for variable and scalar (C++).

The derivative for the variable is

, and

Parameters
:   |  |  |
    | --- | --- |
    | a | First variable operand. |
    | b | Second scalar operand. |

Returns
:   Result of subtracting the scalar from the variable.

Definition at line 113 of file operator\_subtraction.hpp.

|  |  |  |  |
| --- | --- | --- | --- |
| var stan::agrad::operator- | ( | const double | *a*, |
|  |  | const var & | *b* |
|  | ) |  |  |

inline

Subtraction operator for scalar and variable (C++).

The derivative for the variable is

, and

Parameters
:   |  |  |
    | --- | --- |
    | a | First scalar operand. |
    | b | Second variable operand. |

Returns
:   Result of sutracting a variable from a scalar.

Definition at line 130 of file operator\_subtraction.hpp.

|  |  |  |  |  |  |
| --- | --- | --- | --- | --- | --- |
| var& stan::agrad::operator-- | ( | var & | *a* | ) |  |

inline

Prefix decrement operator for variables (C++).

Following C++, `(–a)` is defined to behave exactly as

`a = a - 1.0)`

does, but is faster and uses less memory. In particular, the result is an assignable lvalue.

Parameters
:   |  |  |
    | --- | --- |
    | a | Variable to decrement. |

Returns
:   Reference the result of decrementing this input variable.

Definition at line 39 of file operator\_unary\_decrement.hpp.

|  |  |  |  |
| --- | --- | --- | --- |
| var stan::agrad::operator-- | ( | var & | *a*, |
|  |  | int |  |
|  | ) |  |  |

inline

Postfix decrement operator for variables (C++).

Following C++, the expression `(a–)` is defined to behave like the sequence of operations

`var temp = a; a = a - 1.0; return temp;`

Parameters
:   |  |  |
    | --- | --- |
    | a | Variable to decrement. |

Returns
:   Input variable.

Definition at line 55 of file operator\_unary\_decrement.hpp.

template<typename T >

|  |  |  |  |
| --- | --- | --- | --- |
| fvar<T> stan::agrad::operator/ | ( | const fvar< T > & | *x1*, |
|  |  | const fvar< T > & | *x2* |
|  | ) |  |  |

inline

Definition at line 14 of file operator\_division.hpp.

template<typename T >

|  |  |  |  |
| --- | --- | --- | --- |
| fvar<T> stan::agrad::operator/ | ( | const fvar< T > & | *x1*, |
|  |  | const double | *x2* |
|  | ) |  |  |

inline

Definition at line 22 of file operator\_division.hpp.

template<typename T >

|  |  |  |  |
| --- | --- | --- | --- |
| fvar<T> stan::agrad::operator/ | ( | const double | *x1*, |
|  |  | const fvar< T > & | *x2* |
|  | ) |  |  |

inline

Definition at line 30 of file operator\_division.hpp.

template<typename T , int R, int C>

|  |  |  |  |
| --- | --- | --- | --- |
| Eigen::Matrix<fvar<T>,R,C> stan::agrad::operator/ | ( | const Eigen::Matrix< fvar< T >, R, C > & | *v*, |
|  |  | const fvar< T > & | *c* |
|  | ) |  |  |

inline

Definition at line 58 of file divide.hpp.

template<typename T , int R, int C>

|  |  |  |  |
| --- | --- | --- | --- |
| Eigen::Matrix<fvar<T>,R,C> stan::agrad::operator/ | ( | const Eigen::Matrix< fvar< T >, R, C > & | *v*, |
|  |  | const double | *c* |
|  | ) |  |  |

inline

Definition at line 64 of file divide.hpp.

template<typename T , int R, int C>

|  |  |  |  |
| --- | --- | --- | --- |
| Eigen::Matrix<fvar<T>,R,C> stan::agrad::operator/ | ( | const Eigen::Matrix< double, R, C > & | *v*, |
|  |  | const fvar< T > & | *c* |
|  | ) |  |  |

inline

Definition at line 70 of file divide.hpp.

|  |  |  |  |
| --- | --- | --- | --- |
| var stan::agrad::operator/ | ( | const var & | *a*, |
|  |  | const var & | *b* |
|  | ) |  |  |

inline

Division operator for two variables (C++).

The partial derivatives for the variables are

, and

.

Parameters
:   |  |  |
    | --- | --- |
    | a | First variable operand. |
    | b | Second variable operand. |

Returns
:   Variable result of dividing the first variable by the second.

Definition at line 95 of file operator\_division.hpp.

|  |  |  |  |
| --- | --- | --- | --- |
| var stan::agrad::operator/ | ( | const var & | *a*, |
|  |  | const double | *b* |
|  | ) |  |  |

inline

Division operator for dividing a variable by a scalar (C++).

The derivative with respect to the variable is

.

Parameters
:   |  |  |
    | --- | --- |
    | a | Variable operand. |
    | b | Scalar operand. |

Returns
:   Variable result of dividing the variable by the scalar.

Definition at line 110 of file operator\_division.hpp.

|  |  |  |  |
| --- | --- | --- | --- |
| var stan::agrad::operator/ | ( | const double | *a*, |
|  |  | const var & | *b* |
|  | ) |  |  |

inline

Division operator for dividing a scalar by a variable (C++).

The derivative with respect to the variable is

.

Parameters
:   |  |  |
    | --- | --- |
    | a | Scalar operand. |
    | b | Variable operand. |

Returns
:   Variable result of dividing the scalar by the variable.

Definition at line 127 of file operator\_division.hpp.

template<typename T >

|  |  |  |  |
| --- | --- | --- | --- |
| bool stan::agrad::operator< | ( | const fvar< T > & | *x*, |
|  |  | double | *y* |
|  | ) |  |  |

inline

Definition at line 12 of file operator\_less\_than.hpp.

template<typename T >

|  |  |  |  |
| --- | --- | --- | --- |
| bool stan::agrad::operator< | ( | double | *x*, |
|  |  | const fvar< T > & | *y* |
|  | ) |  |  |

inline

Definition at line 18 of file operator\_less\_than.hpp.

template<typename T >

|  |  |  |  |
| --- | --- | --- | --- |
| bool stan::agrad::operator< | ( | const fvar< T > & | *x*, |
|  |  | const fvar< T > & | *y* |
|  | ) |  |  |

inline

Definition at line 24 of file operator\_less\_than.hpp.

|  |  |  |  |
| --- | --- | --- | --- |
| bool stan::agrad::operator< | ( | const var & | *a*, |
|  |  | const var & | *b* |
|  | ) |  |  |

inline

Less than operator comparing variables' values (C++).

Parameters
:   |  |  |
    | --- | --- |
    | a | First variable. |
    | b | Second variable. |

Returns
:   True if first variable's value is less than second's.

Definition at line 24 of file operator\_less\_than.hpp.

|  |  |  |  |
| --- | --- | --- | --- |
| bool stan::agrad::operator< | ( | const var & | *a*, |
|  |  | const double | *b* |
|  | ) |  |  |

inline

Less than operator comparing variable's value and a double (C++).

Parameters
:   |  |  |
    | --- | --- |
    | a | First variable. |
    | b | Second value. |

Returns
:   True if first variable's value is less than second value.

Definition at line 36 of file operator\_less\_than.hpp.

|  |  |  |  |
| --- | --- | --- | --- |
| bool stan::agrad::operator< | ( | const double | *a*, |
|  |  | const var & | *b* |
|  | ) |  |  |

inline

Less than operator comparing a double and variable's value (C++).

Parameters
:   |  |  |
    | --- | --- |
    | a | First value. |
    | b | Second variable. |

Returns
:   True if first value is less than second variable's value.

Definition at line 48 of file operator\_less\_than.hpp.

template<typename T >

|  |  |  |  |
| --- | --- | --- | --- |
| bool stan::agrad::operator<= | ( | const fvar< T > & | *x*, |
|  |  | const fvar< T > & | *y* |
|  | ) |  |  |

inline

Definition at line 14 of file operator\_less\_than\_or\_equal.hpp.

template<typename T >

|  |  |  |  |
| --- | --- | --- | --- |
| bool stan::agrad::operator<= | ( | const fvar< T > & | *x*, |
|  |  | double | *y* |
|  | ) |  |  |

inline

Definition at line 21 of file operator\_less\_than\_or\_equal.hpp.

|  |  |  |  |
| --- | --- | --- | --- |
| bool stan::agrad::operator<= | ( | const var & | *a*, |
|  |  | const var & | *b* |
|  | ) |  |  |

inline

Less than or equal operator comparing two variables' values (C++).

Parameters
:   |  |  |
    | --- | --- |
    | a | First variable. |
    | b | Second variable. |

Returns
:   True if first variable's value is less than or equal to the second's.

Definition at line 26 of file operator\_less\_than\_or\_equal.hpp.

template<typename T >

|  |  |  |  |
| --- | --- | --- | --- |
| bool stan::agrad::operator<= | ( | double | *x*, |
|  |  | const fvar< T > & | *y* |
|  | ) |  |  |

inline

Definition at line 28 of file operator\_less\_than\_or\_equal.hpp.

|  |  |  |  |
| --- | --- | --- | --- |
| bool stan::agrad::operator<= | ( | const var & | *a*, |
|  |  | const double | *b* |
|  | ) |  |  |

inline

Less than or equal operator comparing a variable's value and a scalar (C++).

Parameters
:   |  |  |
    | --- | --- |
    | a | First variable. |
    | b | Second value. |

Returns
:   True if first variable's value is less than or equal to the second value.

Definition at line 39 of file operator\_less\_than\_or\_equal.hpp.

|  |  |  |  |
| --- | --- | --- | --- |
| bool stan::agrad::operator<= | ( | const double | *a*, |
|  |  | const var & | *b* |
|  | ) |  |  |

inline

Less than or equal operator comparing a double and variable's value (C++).

Parameters
:   |  |  |
    | --- | --- |
    | a | First value. |
    | b | Second variable. |

Returns
:   True if first value is less than or equal to the second variable's value.

Definition at line 52 of file operator\_less\_than\_or\_equal.hpp.

template<typename T >

|  |  |  |  |
| --- | --- | --- | --- |
| bool stan::agrad::operator== | ( | const fvar< T > & | *x*, |
|  |  | const fvar< T > & | *y* |
|  | ) |  |  |

inline

Definition at line 14 of file operator\_equal.hpp.

template<typename T >

|  |  |  |  |
| --- | --- | --- | --- |
| bool stan::agrad::operator== | ( | const fvar< T > & | *x*, |
|  |  | double | *y* |
|  | ) |  |  |

inline

Definition at line 21 of file operator\_equal.hpp.

|  |  |  |  |
| --- | --- | --- | --- |
| bool stan::agrad::operator== | ( | const var & | *a*, |
|  |  | const var & | *b* |
|  | ) |  |  |

inline

Equality operator comparing two variables' values (C++).

Parameters
:   |  |  |
    | --- | --- |
    | a | First variable. |
    | b | Second variable. |

Returns
:   True if the first variable's value is the same as the second's.

Definition at line 26 of file operator\_equal.hpp.

template<typename T >

|  |  |  |  |
| --- | --- | --- | --- |
| bool stan::agrad::operator== | ( | double | *x*, |
|  |  | const fvar< T > & | *y* |
|  | ) |  |  |

inline

Definition at line 28 of file operator\_equal.hpp.

|  |  |  |  |
| --- | --- | --- | --- |
| bool stan::agrad::operator== | ( | const var & | *a*, |
|  |  | const double | *b* |
|  | ) |  |  |

inline

Equality operator comparing a variable's value and a double (C++).

Parameters
:   |  |  |
    | --- | --- |
    | a | First variable. |
    | b | Second value. |

Returns
:   True if the first variable's value is the same as the second value.

Definition at line 39 of file operator\_equal.hpp.

|  |  |  |  |
| --- | --- | --- | --- |
| bool stan::agrad::operator== | ( | const double | *a*, |
|  |  | const var & | *b* |
|  | ) |  |  |

inline

Equality operator comparing a scalar and a variable's value (C++).

Parameters
:   |  |  |
    | --- | --- |
    | a | First scalar. |
    | b | Second variable. |

Returns
:   True if the variable's value is equal to the scalar.

Definition at line 51 of file operator\_equal.hpp.

template<typename T >

|  |  |  |  |
| --- | --- | --- | --- |
| bool stan::agrad::operator> | ( | const fvar< T > & | *x*, |
|  |  | const fvar< T > & | *y* |
|  | ) |  |  |

inline

Definition at line 14 of file operator\_greater\_than.hpp.

template<typename T >

|  |  |  |  |
| --- | --- | --- | --- |
| bool stan::agrad::operator> | ( | const fvar< T > & | *x*, |
|  |  | double | *y* |
|  | ) |  |  |

inline

Definition at line 21 of file operator\_greater\_than.hpp.

|  |  |  |  |
| --- | --- | --- | --- |
| bool stan::agrad::operator> | ( | const var & | *a*, |
|  |  | const var & | *b* |
|  | ) |  |  |

inline

Greater than operator comparing variables' values (C++).

Parameters
:   |  |  |
    | --- | --- |
    | a | First variable. |
    | b | Second variable. |

Returns
:   True if first variable's value is greater than second's.

Definition at line 25 of file operator\_greater\_than.hpp.

template<typename T >

|  |  |  |  |
| --- | --- | --- | --- |
| bool stan::agrad::operator> | ( | double | *x*, |
|  |  | const fvar< T > & | *y* |
|  | ) |  |  |

inline

Definition at line 28 of file operator\_greater\_than.hpp.

|  |  |  |  |
| --- | --- | --- | --- |
| bool stan::agrad::operator> | ( | const var & | *a*, |
|  |  | const double | *b* |
|  | ) |  |  |

inline

Greater than operator comparing variable's value and double (C++).

Parameters
:   |  |  |
    | --- | --- |
    | a | First variable. |
    | b | Second value. |

Returns
:   True if first variable's value is greater than second value.

Definition at line 37 of file operator\_greater\_than.hpp.

|  |  |  |  |
| --- | --- | --- | --- |
| bool stan::agrad::operator> | ( | const double | *a*, |
|  |  | const var & | *b* |
|  | ) |  |  |

inline

Greater than operator comparing a double and a variable's value (C++).

Parameters
:   |  |  |
    | --- | --- |
    | a | First value. |
    | b | Second variable. |

Returns
:   True if first value is greater than second variable's value.

Definition at line 49 of file operator\_greater\_than.hpp.

template<typename T >

|  |  |  |  |
| --- | --- | --- | --- |
| bool stan::agrad::operator>= | ( | const fvar< T > & | *x*, |
|  |  | const fvar< T > & | *y* |
|  | ) |  |  |

inline

Definition at line 14 of file operator\_greater\_than\_or\_equal.hpp.

template<typename T >

|  |  |  |  |
| --- | --- | --- | --- |
| bool stan::agrad::operator>= | ( | const fvar< T > & | *x*, |
|  |  | double | *y* |
|  | ) |  |  |

inline

Definition at line 21 of file operator\_greater\_than\_or\_equal.hpp.

|  |  |  |  |
| --- | --- | --- | --- |
| bool stan::agrad::operator>= | ( | const var & | *a*, |
|  |  | const var & | *b* |
|  | ) |  |  |

inline

Greater than or equal operator comparing two variables' values (C++).

Parameters
:   |  |  |
    | --- | --- |
    | a | First variable. |
    | b | Second variable. |

Returns
:   True if first variable's value is greater than or equal to the second's.

Definition at line 27 of file operator\_greater\_than\_or\_equal.hpp.

template<typename T >

|  |  |  |  |
| --- | --- | --- | --- |
| bool stan::agrad::operator>= | ( | double | *x*, |
|  |  | const fvar< T > & | *y* |
|  | ) |  |  |

inline

Definition at line 28 of file operator\_greater\_than\_or\_equal.hpp.

|  |  |  |  |
| --- | --- | --- | --- |
| bool stan::agrad::operator>= | ( | const var & | *a*, |
|  |  | const double | *b* |
|  | ) |  |  |

inline

Greater than or equal operator comparing variable's value and double (C++).

Parameters
:   |  |  |
    | --- | --- |
    | a | First variable. |
    | b | Second value. |

Returns
:   True if first variable's value is greater than or equal to second value.

Definition at line 40 of file operator\_greater\_than\_or\_equal.hpp.

|  |  |  |  |
| --- | --- | --- | --- |
| bool stan::agrad::operator>= | ( | const double | *a*, |
|  |  | const var & | *b* |
|  | ) |  |  |

inline

Greater than or equal operator comparing double and variable's value (C++).

Parameters
:   |  |  |
    | --- | --- |
    | a | First value. |
    | b | Second variable. |

Returns
:   True if the first value is greater than or equal to the second variable's value.

Definition at line 53 of file operator\_greater\_than\_or\_equal.hpp.

template<typename T >

|  |  |  |  |
| --- | --- | --- | --- |
| fvar<T> stan::agrad::owens\_t | ( | const fvar< T > & | *x1*, |
|  |  | const fvar< T > & | *x2* |
|  | ) |  |  |

inline

Definition at line 17 of file owens\_t.hpp.

template<typename T >

|  |  |  |  |
| --- | --- | --- | --- |
| fvar<T> stan::agrad::owens\_t | ( | const double | *x1*, |
|  |  | const fvar< T > & | *x2* |
|  | ) |  |  |

inline

Definition at line 40 of file owens\_t.hpp.

template<typename T >

|  |  |  |  |
| --- | --- | --- | --- |
| fvar<T> stan::agrad::owens\_t | ( | const fvar< T > & | *x1*, |
|  |  | const double | *x2* |
|  | ) |  |  |

inline

Definition at line 57 of file owens\_t.hpp.

|  |  |  |  |
| --- | --- | --- | --- |
| var stan::agrad::owens\_t | ( | const var & | *h*, |
|  |  | const var & | *a* |
|  | ) |  |  |

inline

The Owen's T function of h and a.

Used to compute the cumulative density function for the skew normal distribution.

Parameters
:   |  |  |
    | --- | --- |
    | h | var parameter. |
    | a | var parameter. |

Returns
:   The Owen's T function.

Definition at line 82 of file owens\_t.hpp.

|  |  |  |  |
| --- | --- | --- | --- |
| var stan::agrad::owens\_t | ( | const var & | *h*, |
|  |  | const double & | *a* |
|  | ) |  |  |

inline

The Owen's T function of h and a.

Used to compute the cumulative density function for the skew normal distribution.

Parameters
:   |  |  |
    | --- | --- |
    | h | var parameter. |
    | a | double parameter. |

Returns
:   The Owen's T function.

Definition at line 98 of file owens\_t.hpp.

|  |  |  |  |
| --- | --- | --- | --- |
| var stan::agrad::owens\_t | ( | const double & | *h*, |
|  |  | const var & | *a* |
|  | ) |  |  |

inline

The Owen's T function of h and a.

Used to compute the cumulative density function for the skew normal distribution.

Parameters
:   |  |  |
    | --- | --- |
    | h | double parameter. |
    | a | var parameter. |

Returns
:   The Owen's T function.

Definition at line 114 of file owens\_t.hpp.

template<typename T , typename F >

|  |  |  |  |
| --- | --- | --- | --- |
| void stan::agrad::partial\_derivative | ( | const F & | *f*, |
|  |  | const Eigen::Matrix< T, Eigen::Dynamic, 1 > & | *x*, |
|  |  | int | *n*, |
|  |  | T & | *fx*, |
|  |  | T & | *dfx\_dxn* |
|  | ) |  |  |

Return the partial derivative of the specified multiivariate function at the specified argument.

Template Parameters
:   |  |  |
    | --- | --- |
    | T | Argument type |
    | F | Function type |

Parameters
:   |  |  |  |
    | --- | --- | --- |
    |  | f | Function |
    | [in] | x | Argument vector |
    | [in] | n | Index of argument with which to take derivative |
    | [out] | fx | Value of function applied to argument |
    | [out] | dfx\_dxn | Value of partial derivative |

Definition at line 49 of file autodiff.hpp.

template<typename T >

|  |  |  |  |  |  |
| --- | --- | --- | --- | --- | --- |
| fvar<T> stan::agrad::Phi | ( | const fvar< T > & | *x* | ) |  |

inline

Definition at line 14 of file Phi.hpp.

|  |  |  |  |  |  |
| --- | --- | --- | --- | --- | --- |
| var stan::agrad::Phi | ( | const stan::agrad::var & | *a* | ) |  |

inline

The unit normal cumulative density function for variables (stan).

See stan::math::Phi() for the double-based version.

The derivative is the unit normal density function,

.

Parameters
:   |  |  |
    | --- | --- |
    | a | Variable argument. |

Returns
:   The unit normal cdf evaluated at the specified argument.

Definition at line 66 of file Phi.hpp.

|  |  |  |  |  |  |
| --- | --- | --- | --- | --- | --- |
| var stan::agrad::Phi\_approx | ( | const stan::agrad::var & | *a* | ) |  |

inline

Approximation of the unit normal CDF for variables (stan).

http://www.jiem.org/index.php/jiem/article/download/60/27

Parameters
:   |  |  |
    | --- | --- |
    | a | Variable argument. |

Returns
:   The corresponding unit normal cdf approximation.

Definition at line 48 of file Phi\_approx.hpp.

template<typename T >

|  |  |  |  |
| --- | --- | --- | --- |
| fvar<T> stan::agrad::pow | ( | const fvar< T > & | *x1*, |
|  |  | const fvar< T > & | *x2* |
|  | ) |  |  |

inline

Definition at line 17 of file pow.hpp.

template<typename T >

|  |  |  |  |
| --- | --- | --- | --- |
| fvar<T> stan::agrad::pow | ( | const double | *x1*, |
|  |  | const fvar< T > & | *x2* |
|  | ) |  |  |

inline

Definition at line 29 of file pow.hpp.

template<typename T >

|  |  |  |  |
| --- | --- | --- | --- |
| fvar<T> stan::agrad::pow | ( | const fvar< T > & | *x1*, |
|  |  | const double | *x2* |
|  | ) |  |  |

inline

Definition at line 39 of file pow.hpp.

|  |  |  |  |
| --- | --- | --- | --- |
| var stan::agrad::pow | ( | const var & | *base*, |
|  |  | const var & | *exponent* |
|  | ) |  |  |

inline

Return the base raised to the power of the exponent (cmath).

The partial derivatives are

, and

.

Parameters
:   |  |  |
    | --- | --- |
    | base | Base variable. |
    | exponent | Exponent variable. |

Returns
:   Base raised to the exponent.

Definition at line 106 of file pow.hpp.

|  |  |  |  |
| --- | --- | --- | --- |
| var stan::agrad::pow | ( | const var & | *base*, |
|  |  | const double | *exponent* |
|  | ) |  |  |

inline

Return the base variable raised to the power of the exponent scalar (cmath).

The derivative for the variable is

.

Parameters
:   |  |  |
    | --- | --- |
    | base | Base variable. |
    | exponent | Exponent scalar. |

Returns
:   Base raised to the exponent.

Definition at line 122 of file pow.hpp.

|  |  |  |  |
| --- | --- | --- | --- |
| var stan::agrad::pow | ( | const double | *base*, |
|  |  | const var & | *exponent* |
|  | ) |  |  |

inline

Return the base scalar raised to the power of the exponent variable (cmath).

The derivative for the variable is

.

Parameters
:   |  |  |
    | --- | --- |
    | base | Base scalar. |
    | exponent | Exponent variable. |

Returns
:   Base raised to the exponent.

Definition at line 144 of file pow.hpp.

|  |  |  |  |
| --- | --- | --- | --- |
| var stan::agrad::precomputed\_gradients | ( | const double | *value*, |
|  |  | const std::vector< var > & | *vars*, |
|  |  | const std::vector< double > & | *gradients* |
|  | ) |  |  |

This function is provided for Stan users that want to compute gradients without using Stan's auto-diff.

Users need to provide the value, the independent variables, and the gradients of this expression with respect to the indepedent variables.

(For advanced users, a faster version that doesn't involve copying vectors exists can be written.)

Parameters
:   |  |  |
    | --- | --- |
    | value | The value of the resulting dependent variable. |
    | vars | The independent variables. |
    | gradients | The value of the gradients of the dependent variable with respect to the independent variables. |

Returns
:   An auto-diff variable that uses the precomputed gradients provided.

Definition at line 86 of file precomputed\_gradients.hpp.

|  |  |  |  |  |  |
| --- | --- | --- | --- | --- | --- |
| double stan::agrad::primitive\_value | ( | const agrad::var & | *v* | ) |  |

inline

Return the primitive double value for the specified auto-diff variable.

Parameters
:   |  |  |
    | --- | --- |
    | v | input variable. |

Returns
:   value of input.

Definition at line 17 of file primitive\_value.hpp.

template<typename T >

|  |  |  |  |  |  |
| --- | --- | --- | --- | --- | --- |
| double stan::agrad::primitive\_value | ( | const fvar< T > & | *v* | ) |  |

inline

Return the primitive value of the specified forward-mode autodiff variable.

This function applies recursively to higher-order autodiff types to return a primitive double value.

Template Parameters
:   |  |  |
    | --- | --- |
    | T | scalar type for autodiff variable. |

Parameters
:   |  |  |
    | --- | --- |
    | v | input variable. |

Returns
:   primitive value of input.

Definition at line 22 of file primitive\_value.hpp.

|  |  |  |  |  |  |
| --- | --- | --- | --- | --- | --- |
| void stan::agrad::print\_stack | ( | std::ostream & | *o* | ) |  |

inline

Prints the auto-dif variable stack.

This function is used for debugging purposes.

Only works if all members of stack are vari\* as it casts to vari\*.

Parameters
:   |  |  |
    | --- | --- |
    | o | ostream to modify |

Definition at line 20 of file print\_stack.hpp.

template<typename T >

|  |  |  |  |  |  |
| --- | --- | --- | --- | --- | --- |
| Eigen::Matrix<fvar<T>,Eigen::Dynamic,Eigen::Dynamic> stan::agrad::qr\_Q | ( | const Eigen::Matrix< fvar< T >, Eigen::Dynamic, Eigen::Dynamic > & | *m* | ) |  |

Definition at line 15 of file qr\_Q.hpp.

template<typename T >

|  |  |  |  |  |  |
| --- | --- | --- | --- | --- | --- |
| Eigen::Matrix<fvar<T>,Eigen::Dynamic,Eigen::Dynamic> stan::agrad::qr\_R | ( | const Eigen::Matrix< fvar< T >, Eigen::Dynamic, Eigen::Dynamic > & | *m* | ) |  |

Definition at line 15 of file qr\_R.hpp.

template<typename TA , int RA, int CA, typename TB , int RB, int CB>

|  |  |  |  |
| --- | --- | --- | --- |
| boost::enable\_if\_c< boost::is\_same<TA,var>::value \|\| boost::is\_same<TB,var>::value, Eigen::Matrix<var,CB,CB> >::type stan::agrad::quad\_form | ( | const Eigen::Matrix< TA, RA, CA > & | *A*, |
|  |  | const Eigen::Matrix< TB, RB, CB > & | *B* |
|  | ) |  |  |

inline

Definition at line 129 of file quad\_form.hpp.

template<typename TA , int RA, int CA, typename TB , int RB>

|  |  |  |  |
| --- | --- | --- | --- |
| boost::enable\_if\_c< boost::is\_same<TA,var>::value \|\| boost::is\_same<TB,var>::value, var >::type stan::agrad::quad\_form | ( | const Eigen::Matrix< TA, RA, CA > & | *A*, |
|  |  | const Eigen::Matrix< TB, RB, 1 > & | *B* |
|  | ) |  |  |

inline

Definition at line 145 of file quad\_form.hpp.

template<typename TA , int RA, int CA, typename TB , int RB, int CB>

|  |  |  |  |
| --- | --- | --- | --- |
| boost::enable\_if\_c< boost::is\_same<TA,var>::value \|\| boost::is\_same<TB,var>::value, Eigen::Matrix<var,CB,CB> >::type stan::agrad::quad\_form\_sym | ( | const Eigen::Matrix< TA, RA, CA > & | *A*, |
|  |  | const Eigen::Matrix< TB, RB, CB > & | *B* |
|  | ) |  |  |

inline

Definition at line 162 of file quad\_form.hpp.

template<typename TA , int RA, int CA, typename TB , int RB>

|  |  |  |  |
| --- | --- | --- | --- |
| boost::enable\_if\_c< boost::is\_same<TA,var>::value \|\| boost::is\_same<TB,var>::value, var >::type stan::agrad::quad\_form\_sym | ( | const Eigen::Matrix< TA, RA, CA > & | *A*, |
|  |  | const Eigen::Matrix< TB, RB, 1 > & | *B* |
|  | ) |  |  |

inline

Definition at line 179 of file quad\_form.hpp.

|  |  |  |  |  |
| --- | --- | --- | --- | --- |
| static void stan::agrad::recover\_memory | ( |  | ) |  |

inlinestatic

Recover memory used for all variables for reuse.

Exceptions
:   |  |  |
    | --- | --- |
    | std::logic\_error | if `empty_nested()` returns `false` |

Definition at line 52 of file var\_stack.hpp.

|  |  |  |  |  |
| --- | --- | --- | --- | --- |
| static void stan::agrad::recover\_memory\_nested | ( |  | ) |  |

inlinestatic

Recover only the memory used for the top nested call.

If there is nothing on the nested stack, then a `std::logic_error` exception is thrown.

Exceptions
:   |  |  |
    | --- | --- |
    | std::logic\_error | if `empty_nested()` returns `true` |

Definition at line 72 of file var\_stack.hpp.

template<typename T >

|  |  |  |  |
| --- | --- | --- | --- |
| fvar<T> stan::agrad::rising\_factorial | ( | const fvar< T > & | *x*, |
|  |  | const fvar< T > & | *n* |
|  | ) |  |  |

inline

Definition at line 16 of file rising\_factorial.hpp.

template<typename T >

|  |  |  |  |
| --- | --- | --- | --- |
| fvar<T> stan::agrad::rising\_factorial | ( | const fvar< T > & | *x*, |
|  |  | const double | *n* |
|  | ) |  |  |

inline

Definition at line 28 of file rising\_factorial.hpp.

template<typename T >

|  |  |  |  |
| --- | --- | --- | --- |
| fvar<T> stan::agrad::rising\_factorial | ( | const double | *x*, |
|  |  | const fvar< T > & | *n* |
|  | ) |  |  |

inline

Definition at line 40 of file rising\_factorial.hpp.

|  |  |  |  |
| --- | --- | --- | --- |
| var stan::agrad::rising\_factorial | ( | const var & | *a*, |
|  |  | const double & | *b* |
|  | ) |  |  |

inline

Definition at line 48 of file rising\_factorial.hpp.

|  |  |  |  |
| --- | --- | --- | --- |
| var stan::agrad::rising\_factorial | ( | const var & | *a*, |
|  |  | const var & | *b* |
|  | ) |  |  |

inline

Definition at line 53 of file rising\_factorial.hpp.

|  |  |  |  |
| --- | --- | --- | --- |
| var stan::agrad::rising\_factorial | ( | const double & | *a*, |
|  |  | const var & | *b* |
|  | ) |  |  |

inline

Definition at line 58 of file rising\_factorial.hpp.

template<typename T >

|  |  |  |  |  |  |
| --- | --- | --- | --- | --- | --- |
| fvar<T> stan::agrad::round | ( | const fvar< T > & | *x* | ) |  |

inline

Definition at line 15 of file round.hpp.

|  |  |  |  |  |  |
| --- | --- | --- | --- | --- | --- |
| var stan::agrad::round | ( | const stan::agrad::var & | *a* | ) |  |

inline

Returns the rounded form of the specified variable (C99).

See boost::math::round() for the double-based version.

The derivative is zero everywhere but numbers half way between whole numbers, so for convenience the derivative is defined to be everywhere zero,

.

Parameters
:   |  |  |
    | --- | --- |
    | a | Specified variable. |

Returns
:   Rounded variable.

Definition at line 57 of file round.hpp.

template<typename T , int R1, int C1, int R2, int C2>

|  |  |  |  |
| --- | --- | --- | --- |
| Eigen::Matrix<fvar<T>, R1, 1> stan::agrad::rows\_dot\_product | ( | const Eigen::Matrix< fvar< T >, R1, C1 > & | *v1*, |
|  |  | const Eigen::Matrix< fvar< T >, R2, C2 > & | *v2* |
|  | ) |  |  |

inline

Definition at line 19 of file rows\_dot\_product.hpp.

template<typename T , int R1, int C1, int R2, int C2>

|  |  |  |  |
| --- | --- | --- | --- |
| Eigen::Matrix<fvar<T>, R1, 1> stan::agrad::rows\_dot\_product | ( | const Eigen::Matrix< double, R1, C1 > & | *v1*, |
|  |  | const Eigen::Matrix< fvar< T >, R2, C2 > & | *v2* |
|  | ) |  |  |

inline

Definition at line 35 of file rows\_dot\_product.hpp.

template<typename T , int R1, int C1, int R2, int C2>

|  |  |  |  |
| --- | --- | --- | --- |
| Eigen::Matrix<fvar<T>, R1, 1> stan::agrad::rows\_dot\_product | ( | const Eigen::Matrix< fvar< T >, R1, C1 > & | *v1*, |
|  |  | const Eigen::Matrix< double, R2, C2 > & | *v2* |
|  | ) |  |  |

inline

Definition at line 51 of file rows\_dot\_product.hpp.

template<typename T1 , int R1, int C1, typename T2 , int R2, int C2>

|  |  |  |  |
| --- | --- | --- | --- |
| boost::enable\_if\_c<boost::is\_same<T1,var>::value \|\| boost::is\_same<T2,var>::value, Eigen::Matrix<var, R1, 1> >::type stan::agrad::rows\_dot\_product | ( | const Eigen::Matrix< T1, R1, C1 > & | *v1*, |
|  |  | const Eigen::Matrix< T2, R2, C2 > & | *v2* |
|  | ) |  |  |

inline

Definition at line 277 of file dot\_product.hpp.

template<typename T , int R, int C>

|  |  |  |  |  |  |
| --- | --- | --- | --- | --- | --- |
| Eigen::Matrix<fvar<T>,R,1> stan::agrad::rows\_dot\_self | ( | const Eigen::Matrix< fvar< T >, R, C > & | *x* | ) |  |

inline

Definition at line 15 of file rows\_dot\_self.hpp.

|  |  |  |  |  |  |
| --- | --- | --- | --- | --- | --- |
| var stan::agrad::sd | ( | const std::vector< var > & | *v* | ) |  |

Return the sample standard deviation of the specified standard vector.

Raise domain error if size is not greater than zero.

Parameters
:   |  |  |  |
    | --- | --- | --- |
    | [in] | v | a vector |

Returns
:   sample standard deviation of specified vector

Definition at line 63 of file sd.hpp.

template<int R, int C>

|  |  |  |  |  |  |
| --- | --- | --- | --- | --- | --- |
| var stan::agrad::sd | ( | const Eigen::Matrix< var, R, C > & | *m* | ) |  |

Definition at line 80 of file sd.hpp.

|  |  |  |  |  |
| --- | --- | --- | --- | --- |
| static void stan::agrad::set\_zero\_all\_adjoints | ( |  | ) |  |

static

Reset all adjoint values in the stack to zero.

Definition at line 87 of file chainable.hpp.

template<typename T >

|  |  |  |  |  |  |
| --- | --- | --- | --- | --- | --- |
| fvar<T> stan::agrad::sin | ( | const fvar< T > & | *x* | ) |  |

inline

Definition at line 14 of file sin.hpp.

|  |  |  |  |  |  |
| --- | --- | --- | --- | --- | --- |
| var stan::agrad::sin | ( | const var & | *a* | ) |  |

inline

Return the sine of a radian-scaled variable (cmath).

The derivative is defined by

.

Parameters
:   |  |  |
    | --- | --- |
    | a | Variable for radians of angle. |

Returns
:   Sine of variable.

Definition at line 50 of file sin.hpp.

template<typename T >

|  |  |  |  |  |  |
| --- | --- | --- | --- | --- | --- |
| fvar<T> stan::agrad::sinh | ( | const fvar< T > & | *x* | ) |  |

inline

Definition at line 14 of file sinh.hpp.

|  |  |  |  |  |  |
| --- | --- | --- | --- | --- | --- |
| var stan::agrad::sinh | ( | const var & | *a* | ) |  |

inline

Return the hyperbolic sine of the specified variable (cmath).

The derivative is defined by

.

Parameters
:   |  |  |
    | --- | --- |
    | a | Variable. |

Returns
:   Hyperbolic sine of variable.

Definition at line 50 of file sinh.hpp.

template<typename T >

|  |  |  |  |  |  |
| --- | --- | --- | --- | --- | --- |
| Eigen::Matrix<fvar<T>,Eigen::Dynamic,1> stan::agrad::softmax | ( | const Eigen::Matrix< fvar< T >, Eigen::Dynamic, 1 > & | *alpha* | ) |  |

inline

Definition at line 14 of file softmax.hpp.

|  |  |  |  |  |  |
| --- | --- | --- | --- | --- | --- |
| Eigen::Matrix<var,Eigen::Dynamic,1> stan::agrad::softmax | ( | const Eigen::Matrix< var, Eigen::Dynamic, 1 > & | *alpha* | ) |  |

inline

Return the softmax of the specified Eigen vector.

Softmax is guaranteed to return a simplex.

The gradient calculations are unfolded.

Parameters
:   |  |  |
    | --- | --- |
    | alpha | Unconstrained input vector. |

Returns
:   Softmax of the input.

Exceptions
:   |  |  |
    | --- | --- |
    | std::domain\_error | If the input vector is size 0. |

Definition at line 58 of file softmax.hpp.

template<typename T >

|  |  |  |  |  |  |
| --- | --- | --- | --- | --- | --- |
| std::vector< fvar<T> > stan::agrad::sort\_asc | ( | std::vector< fvar< T > > | *xs* | ) |  |

inline

Definition at line 18 of file sort.hpp.

|  |  |  |  |  |  |
| --- | --- | --- | --- | --- | --- |
| std::vector<var> stan::agrad::sort\_asc | ( | std::vector< var > | *xs* | ) |  |

inline

Return the specified standard vector in ascending order with gradients kept.

Parameters
:   |  |  |
    | --- | --- |
    | xs | Standard vector to order. |

Returns
:   Standard vector ordered.

Template Parameters
:   |  |  |
    | --- | --- |
    | T | Type of elements of the vector. |

Definition at line 24 of file sort.hpp.

template<typename T , int R, int C>

|  |  |  |  |  |  |
| --- | --- | --- | --- | --- | --- |
| Eigen::Matrix<fvar<T>,R,C> stan::agrad::sort\_asc | ( | Eigen::Matrix< fvar< T >, R, C > | *xs* | ) |  |

inline

Definition at line 34 of file sort.hpp.

template<int R, int C>

|  |  |  |  |  |  |
| --- | --- | --- | --- | --- | --- |
| Eigen::Matrix<var,R,C> stan::agrad::sort\_asc | ( | Eigen::Matrix< var, R, C > | *xs* | ) |  |

inline

Return the specified eigen vector in ascending order with gradients kept.

Parameters
:   |  |  |
    | --- | --- |
    | xs | Eigen vector to order. |

Returns
:   Eigen vector ordered.

Template Parameters
:   |  |  |
    | --- | --- |
    | T | Type of elements of the vector. |

Definition at line 49 of file sort.hpp.

template<typename T >

|  |  |  |  |  |  |
| --- | --- | --- | --- | --- | --- |
| std::vector< fvar<T> > stan::agrad::sort\_desc | ( | std::vector< fvar< T > > | *xs* | ) |  |

inline

Definition at line 26 of file sort.hpp.

|  |  |  |  |  |  |
| --- | --- | --- | --- | --- | --- |
| std::vector<var> stan::agrad::sort\_desc | ( | std::vector< var > | *xs* | ) |  |

inline

Return the specified standard vector in descending order with gradients kept.

Parameters
:   |  |  |
    | --- | --- |
    | xs | Standard vector to order. |

Returns
:   Standard vector ordered.

Template Parameters
:   |  |  |
    | --- | --- |
    | T | Type of elements of the vector. |

Definition at line 36 of file sort.hpp.

template<typename T , int R, int C>

|  |  |  |  |  |  |
| --- | --- | --- | --- | --- | --- |
| Eigen::Matrix<fvar<T>,R,C> stan::agrad::sort\_desc | ( | Eigen::Matrix< fvar< T >, R, C > | *xs* | ) |  |

inline

Definition at line 42 of file sort.hpp.

template<int R, int C>

|  |  |  |  |  |  |
| --- | --- | --- | --- | --- | --- |
| Eigen::Matrix<var,R,C> stan::agrad::sort\_desc | ( | Eigen::Matrix< var, R, C > | *xs* | ) |  |

inline

Return the specified eigen vector in descending order with gradients kept.

Parameters
:   |  |  |
    | --- | --- |
    | xs | Eigen vector to order. |

Returns
:   Eigen vector ordered.

Template Parameters
:   |  |  |
    | --- | --- |
    | T | Type of elements of the vector. |

Definition at line 62 of file sort.hpp.

template<typename T >

|  |  |  |  |  |  |
| --- | --- | --- | --- | --- | --- |
| fvar<T> stan::agrad::sqrt | ( | const fvar< T > & | *x* | ) |  |

inline

Definition at line 15 of file sqrt.hpp.

|  |  |  |  |  |  |
| --- | --- | --- | --- | --- | --- |
| var stan::agrad::sqrt | ( | const var & | *a* | ) |  |

inline

Return the square root of the specified variable (cmath).

The derivative is defined by

.

Parameters
:   |  |  |
    | --- | --- |
    | a | Variable whose square root is taken. |

Returns
:   Square root of variable.

Definition at line 51 of file sqrt.hpp.

template<typename T >

|  |  |  |  |  |  |
| --- | --- | --- | --- | --- | --- |
| fvar<T> stan::agrad::square | ( | const fvar< T > & | *x* | ) |  |

inline

Definition at line 15 of file square.hpp.

|  |  |  |  |  |  |
| --- | --- | --- | --- | --- | --- |
| var stan::agrad::square | ( | const var & | *x* | ) |  |

inline

Return the square of the input variable.

Using `square(x)` is more efficient than using `x * x`.

Parameters
:   |  |  |
    | --- | --- |
    | x | Variable to square. |

Returns
:   Square of variable.

Definition at line 47 of file square.hpp.

template<int R1, int C1, int R2, int C2>

|  |  |  |  |
| --- | --- | --- | --- |
| var stan::agrad::squared\_distance | ( | const Eigen::Matrix< var, R1, C1 > & | *v1*, |
|  |  | const Eigen::Matrix< var, R2, C2 > & | *v2* |
|  | ) |  |  |

inline

Definition at line 112 of file squared\_distance.hpp.

template<int R1, int C1, int R2, int C2>

|  |  |  |  |
| --- | --- | --- | --- |
| var stan::agrad::squared\_distance | ( | const Eigen::Matrix< var, R1, C1 > & | *v1*, |
|  |  | const Eigen::Matrix< double, R2, C2 > & | *v2* |
|  | ) |  |  |

inline

Definition at line 121 of file squared\_distance.hpp.

template<int R1, int C1, int R2, int C2>

|  |  |  |  |
| --- | --- | --- | --- |
| var stan::agrad::squared\_distance | ( | const Eigen::Matrix< double, R1, C1 > & | *v1*, |
|  |  | const Eigen::Matrix< var, R2, C2 > & | *v2* |
|  | ) |  |  |

inline

Definition at line 130 of file squared\_distance.hpp.

|  |  |  |  |
| --- | --- | --- | --- |
| void stan::agrad::stan\_print | ( | std::ostream \* | *o*, |
|  |  | const var & | *x* |
|  | ) |  |  |

Definition at line 10 of file stan\_print.hpp.

|  |  |  |  |  |
| --- | --- | --- | --- | --- |
| static void stan::agrad::start\_nested | ( |  | ) |  |

inlinestatic

Record the current position so that `recover_memory_nested()` can find it.

Definition at line 96 of file var\_stack.hpp.

|  |  |  |  |  |  |
| --- | --- | --- | --- | --- | --- |
| var stan::agrad::step | ( | const stan::agrad::var & | *a* | ) |  |

inline

Return the step, or heaviside, function applied to the specified variable (stan).

See stan::math::step() for the double-based version.

The derivative of the step function is zero everywhere but at 0, so for convenience, it is taken to be everywhere zero,

.

Parameters
:   |  |  |
    | --- | --- |
    | a | Variable argument. |

Returns
:   The constant variable with value 1.0 if the argument's value is greater than or equal to 0.0, and value 0.0 otherwise.

Definition at line 25 of file step.hpp.

template<typename T , int R, int C>

|  |  |  |  |  |  |
| --- | --- | --- | --- | --- | --- |
| fvar<T> stan::agrad::sum | ( | const Eigen::Matrix< fvar< T >, R, C > & | *m* | ) |  |

inline

Definition at line 14 of file sum.hpp.

template<int R, int C>

|  |  |  |  |  |  |
| --- | --- | --- | --- | --- | --- |
| var stan::agrad::sum | ( | const Eigen::Matrix< var, R, C > & | *m* | ) |  |

inline

Returns the sum of the coefficients of the specified matrix, column vector or row vector.

Parameters
:   |  |  |
    | --- | --- |
    | m | Specified matrix or vector. |

Returns
:   Sum of coefficients of matrix.

Definition at line 68 of file sum.hpp.

template<typename T >

|  |  |  |  |  |  |
| --- | --- | --- | --- | --- | --- |
| fvar<T> stan::agrad::tan | ( | const fvar< T > & | *x* | ) |  |

inline

Definition at line 14 of file tan.hpp.

|  |  |  |  |  |  |
| --- | --- | --- | --- | --- | --- |
| var stan::agrad::tan | ( | const var & | *a* | ) |  |

inline

Return the tangent of a radian-scaled variable (cmath).

The derivative is defined by

.

Parameters
:   |  |  |
    | --- | --- |
    | a | Variable for radians of angle. |

Returns
:   Tangent of variable.

Definition at line 50 of file tan.hpp.

template<typename T >

|  |  |  |  |  |  |
| --- | --- | --- | --- | --- | --- |
| fvar<T> stan::agrad::tanh | ( | const fvar< T > & | *x* | ) |  |

inline

Definition at line 14 of file tanh.hpp.

|  |  |  |  |  |  |
| --- | --- | --- | --- | --- | --- |
| var stan::agrad::tanh | ( | const var & | *a* | ) |  |

inline

Return the hyperbolic tangent of the specified variable (cmath).

The derivative is defined by

.

Parameters
:   |  |  |
    | --- | --- |
    | a | Variable. |

Returns
:   Hyperbolic tangent of variable.

Definition at line 51 of file tanh.hpp.

template<typename T , int R, int C>

|  |  |  |  |  |  |
| --- | --- | --- | --- | --- | --- |
| Eigen::Matrix<fvar<T>,R,R> stan::agrad::tcrossprod | ( | const Eigen::Matrix< fvar< T >, R, C > & | *m* | ) |  |

inline

Definition at line 17 of file tcrossprod.hpp.

|  |  |  |  |  |  |
| --- | --- | --- | --- | --- | --- |
| matrix\_v stan::agrad::tcrossprod | ( | const matrix\_v & | *M* | ) |  |

inline

Returns the result of post-multiplying a matrix by its own transpose.

Parameters
:   |  |  |
    | --- | --- |
    | M | Matrix to multiply. |

Returns
:   M times its transpose.

Definition at line 24 of file tcrossprod.hpp.

template<typename T >

|  |  |  |  |  |  |
| --- | --- | --- | --- | --- | --- |
| fvar<T> stan::agrad::tgamma | ( | const fvar< T > & | *x* | ) |  |

inline

Definition at line 15 of file tgamma.hpp.

|  |  |  |  |  |  |
| --- | --- | --- | --- | --- | --- |
| var stan::agrad::tgamma | ( | const stan::agrad::var & | *a* | ) |  |

inline

Return the Gamma function applied to the specified variable (C99).

See boost::math::tgamma() for the double-based version.

The derivative with respect to the argument is

where  is the digamma function.

See boost::math::digamma() for the double-based version.

Parameters
:   |  |  |
    | --- | --- |
    | a | Argument to function. |

Returns
:   The Gamma function applied to the specified argument.

Definition at line 66 of file tgamma.hpp.

template<typename T >

|  |  |  |  |  |  |
| --- | --- | --- | --- | --- | --- |
| fvar<T> stan::agrad::to\_fvar | ( | const T & | *x* | ) |  |

inline

Definition at line 17 of file to\_fvar.hpp.

template<typename T >

|  |  |  |  |  |  |
| --- | --- | --- | --- | --- | --- |
| fvar<T> stan::agrad::to\_fvar | ( | const fvar< T > & | *x* | ) |  |

inline

Definition at line 24 of file to\_fvar.hpp.

|  |  |  |  |  |  |
| --- | --- | --- | --- | --- | --- |
| matrix\_fd stan::agrad::to\_fvar | ( | const stan::math::matrix\_d & | *m* | ) |  |

inline

Definition at line 30 of file to\_fvar.hpp.

|  |  |  |  |  |  |
| --- | --- | --- | --- | --- | --- |
| matrix\_fd stan::agrad::to\_fvar | ( | const matrix\_fd & | *m* | ) |  |

inline

Definition at line 40 of file to\_fvar.hpp.

|  |  |  |  |  |  |
| --- | --- | --- | --- | --- | --- |
| matrix\_fv stan::agrad::to\_fvar | ( | const matrix\_fv & | *m* | ) |  |

inline

Definition at line 46 of file to\_fvar.hpp.

|  |  |  |  |  |  |
| --- | --- | --- | --- | --- | --- |
| matrix\_ffd stan::agrad::to\_fvar | ( | const matrix\_ffd & | *m* | ) |  |

inline

Definition at line 52 of file to\_fvar.hpp.

|  |  |  |  |  |  |
| --- | --- | --- | --- | --- | --- |
| matrix\_ffv stan::agrad::to\_fvar | ( | const matrix\_ffv & | *m* | ) |  |

inline

Definition at line 58 of file to\_fvar.hpp.

|  |  |  |  |  |  |
| --- | --- | --- | --- | --- | --- |
| vector\_fd stan::agrad::to\_fvar | ( | const stan::math::vector\_d & | *v* | ) |  |

inline

Definition at line 64 of file to\_fvar.hpp.

|  |  |  |  |  |  |
| --- | --- | --- | --- | --- | --- |
| vector\_fd stan::agrad::to\_fvar | ( | const vector\_fd & | *v* | ) |  |

inline

Definition at line 73 of file to\_fvar.hpp.

|  |  |  |  |  |  |
| --- | --- | --- | --- | --- | --- |
| vector\_fv stan::agrad::to\_fvar | ( | const vector\_fv & | *v* | ) |  |

inline

Definition at line 79 of file to\_fvar.hpp.

|  |  |  |  |  |  |
| --- | --- | --- | --- | --- | --- |
| vector\_ffd stan::agrad::to\_fvar | ( | const vector\_ffd & | *v* | ) |  |

inline

Definition at line 85 of file to\_fvar.hpp.

|  |  |  |  |  |  |
| --- | --- | --- | --- | --- | --- |
| vector\_ffv stan::agrad::to\_fvar | ( | const vector\_ffv & | *v* | ) |  |

inline

Definition at line 91 of file to\_fvar.hpp.

|  |  |  |  |  |  |
| --- | --- | --- | --- | --- | --- |
| row\_vector\_fd stan::agrad::to\_fvar | ( | const stan::math::row\_vector\_d & | *rv* | ) |  |

inline

Definition at line 97 of file to\_fvar.hpp.

|  |  |  |  |  |  |
| --- | --- | --- | --- | --- | --- |
| row\_vector\_fd stan::agrad::to\_fvar | ( | const row\_vector\_fd & | *rv* | ) |  |

inline

Definition at line 106 of file to\_fvar.hpp.

|  |  |  |  |  |  |
| --- | --- | --- | --- | --- | --- |
| row\_vector\_fv stan::agrad::to\_fvar | ( | const row\_vector\_fv & | *rv* | ) |  |

inline

Definition at line 112 of file to\_fvar.hpp.

|  |  |  |  |  |  |
| --- | --- | --- | --- | --- | --- |
| row\_vector\_ffd stan::agrad::to\_fvar | ( | const row\_vector\_ffd & | *rv* | ) |  |

inline

Definition at line 118 of file to\_fvar.hpp.

|  |  |  |  |  |  |
| --- | --- | --- | --- | --- | --- |
| row\_vector\_ffv stan::agrad::to\_fvar | ( | const row\_vector\_ffv & | *rv* | ) |  |

inline

Definition at line 124 of file to\_fvar.hpp.

template<typename T , int R, int C>

|  |  |  |  |
| --- | --- | --- | --- |
| Eigen::Matrix<fvar<T>, R, C> stan::agrad::to\_fvar | ( | const Eigen::Matrix< T, R, C > & | *val*, |
|  |  | const Eigen::Matrix< T, R, C > & | *deriv* |
|  | ) |  |  |

inline

Definition at line 131 of file to\_fvar.hpp.

|  |  |  |  |  |  |
| --- | --- | --- | --- | --- | --- |
| var stan::agrad::to\_var | ( | const double & | *x* | ) |  |

inline

Converts argument to an automatic differentiation variable.

Returns a stan::agrad::var variable with the input value.

Parameters
:   |  |  |  |
    | --- | --- | --- |
    | [in] | x | A scalar value |

Returns
:   An automatic differentiation variable with the input value.

Definition at line 21 of file to\_var.hpp.

|  |  |  |  |  |  |
| --- | --- | --- | --- | --- | --- |
| var stan::agrad::to\_var | ( | const var & | *x* | ) |  |

inline

Converts argument to an automatic differentiation variable.

Returns a stan::agrad::var variable with the input value.

Parameters
:   |  |  |  |
    | --- | --- | --- |
    | [in] | x | An automatic differentiation variable. |

Returns
:   An automatic differentiation variable with the input value.

Definition at line 32 of file to\_var.hpp.

|  |  |  |  |  |  |
| --- | --- | --- | --- | --- | --- |
| matrix\_v stan::agrad::to\_var | ( | const stan::math::matrix\_d & | *m* | ) |  |

inline

Converts argument to an automatic differentiation variable.

Returns a stan::agrad::var variable with the input value.

Parameters
:   |  |  |  |
    | --- | --- | --- |
    | [in] | m | A Matrix with scalars |

Returns
:   A Matrix with automatic differentiation variables

Definition at line 43 of file to\_var.hpp.

|  |  |  |  |  |  |
| --- | --- | --- | --- | --- | --- |
| matrix\_v stan::agrad::to\_var | ( | const matrix\_v & | *m* | ) |  |

inline

Converts argument to an automatic differentiation variable.

Returns a stan::agrad::var variable with the input value.

Parameters
:   |  |  |  |
    | --- | --- | --- |
    | [in] | m | A Matrix with automatic differentiation variables. |

Returns
:   A Matrix with automatic differentiation variables.

Definition at line 58 of file to\_var.hpp.

|  |  |  |  |  |  |
| --- | --- | --- | --- | --- | --- |
| vector\_v stan::agrad::to\_var | ( | const stan::math::vector\_d & | *v* | ) |  |

inline

Converts argument to an automatic differentiation variable.

Returns a stan::agrad::var variable with the input value.

Parameters
:   |  |  |  |
    | --- | --- | --- |
    | [in] | v | A Vector of scalars |

Returns
:   A Vector of automatic differentiation variables with values of v

Definition at line 70 of file to\_var.hpp.

|  |  |  |  |  |  |
| --- | --- | --- | --- | --- | --- |
| vector\_v stan::agrad::to\_var | ( | const vector\_v & | *v* | ) |  |

inline

Converts argument to an automatic differentiation variable.

Returns a stan::agrad::var variable with the input value.

Parameters
:   |  |  |  |
    | --- | --- | --- |
    | [in] | v | A Vector of automatic differentiation variables |

Returns
:   A Vector of automatic differentiation variables with values of v

Definition at line 85 of file to\_var.hpp.

|  |  |  |  |  |  |
| --- | --- | --- | --- | --- | --- |
| row\_vector\_v stan::agrad::to\_var | ( | const stan::math::row\_vector\_d & | *rv* | ) |  |

inline

Converts argument to an automatic differentiation variable.

Returns a stan::agrad::var variable with the input value.

Parameters
:   |  |  |  |
    | --- | --- | --- |
    | [in] | rv | A row vector of scalars |

Returns
:   A row vector of automatic differentation variables with values of rv.

Definition at line 97 of file to\_var.hpp.

|  |  |  |  |  |  |
| --- | --- | --- | --- | --- | --- |
| row\_vector\_v stan::agrad::to\_var | ( | const row\_vector\_v & | *rv* | ) |  |

inline

Converts argument to an automatic differentiation variable.

Returns a stan::agrad::var variable with the input value.

Parameters
:   |  |  |  |
    | --- | --- | --- |
    | [in] | rv | A row vector with automatic differentiation variables |

Returns
:   A row vector with automatic differentiation variables with values of rv.

Definition at line 112 of file to\_var.hpp.

template<typename T1 , int R1, int C1, typename T2 , int R2, int C2, typename T3 , int R3, int C3>

|  |  |  |  |
| --- | --- | --- | --- |
| boost::enable\_if\_c<boost::is\_same<T1,var>::value \|\| boost::is\_same<T2,var>::value \|\| boost::is\_same<T3,var>::value, var>::type stan::agrad::trace\_gen\_inv\_quad\_form\_ldlt | ( | const Eigen::Matrix< T1, R1, C1 > & | *D*, |
|  |  | const stan::math::LDLT\_factor< T2, R2, C2 > & | *A*, |
|  |  | const Eigen::Matrix< T3, R3, C3 > & | *B* |
|  | ) |  |  |

inline

Compute the trace of an inverse quadratic form.

I.E., this computes trace(D B^T A^-1 B) where D is a square matrix and the LDLT\_factor of A is provided.

Definition at line 25 of file trace\_gen\_inv\_quad\_form\_ldlt.hpp.

template<int RD, int CD, int RA, int CA, int RB, int CB, typename T >

|  |  |  |  |
| --- | --- | --- | --- |
| fvar<T> stan::agrad::trace\_gen\_quad\_form | ( | const Eigen::Matrix< fvar< T >, RD, CD > & | *D*, |
|  |  | const Eigen::Matrix< fvar< T >, RA, CA > & | *A*, |
|  |  | const Eigen::Matrix< fvar< T >, RB, CB > & | *B* |
|  | ) |  |  |

inline

Definition at line 15 of file trace\_gen\_quad\_form.hpp.

template<typename TD , int RD, int CD, typename TA , int RA, int CA, typename TB , int RB, int CB>

|  |  |  |  |
| --- | --- | --- | --- |
| boost::enable\_if\_c< boost::is\_same<TD,var>::value \|\| boost::is\_same<TA,var>::value \|\| boost::is\_same<TB,var>::value, var >::type stan::agrad::trace\_gen\_quad\_form | ( | const Eigen::Matrix< TD, RD, CD > & | *D*, |
|  |  | const Eigen::Matrix< TA, RA, CA > & | *A*, |
|  |  | const Eigen::Matrix< TB, RB, CB > & | *B* |
|  | ) |  |  |

inline

Definition at line 111 of file trace\_gen\_quad\_form.hpp.

template<typename T2 , int R2, int C2, typename T3 , int R3, int C3>

|  |  |  |  |
| --- | --- | --- | --- |
| boost::enable\_if\_c<boost::is\_same<T2,var>::value \|\| boost::is\_same<T3,var>::value, var>::type stan::agrad::trace\_inv\_quad\_form\_ldlt | ( | const stan::math::LDLT\_factor< T2, R2, C2 > & | *A*, |
|  |  | const Eigen::Matrix< T3, R3, C3 > & | *B* |
|  | ) |  |  |

inline

Compute the trace of an inverse quadratic form.

I.E., this computes trace(B^T A^-1 B) where the LDLT\_factor of A is provided.

Definition at line 162 of file trace\_inv\_quad\_form\_ldlt.hpp.

template<int RA, int CA, int RB, int CB, typename T >

|  |  |  |  |
| --- | --- | --- | --- |
| stan::agrad::fvar<T> stan::agrad::trace\_quad\_form | ( | const Eigen::Matrix< stan::agrad::fvar< T >, RA, CA > & | *A*, |
|  |  | const Eigen::Matrix< stan::agrad::fvar< T >, RB, CB > & | *B* |
|  | ) |  |  |

inline

Definition at line 19 of file trace\_quad\_form.hpp.

template<typename TA , int RA, int CA, typename TB , int RB, int CB>

|  |  |  |  |
| --- | --- | --- | --- |
| boost::enable\_if\_c< boost::is\_same<TA,var>::value \|\| boost::is\_same<TB,var>::value, var >::type stan::agrad::trace\_quad\_form | ( | const Eigen::Matrix< TA, RA, CA > & | *A*, |
|  |  | const Eigen::Matrix< TB, RB, CB > & | *B* |
|  | ) |  |  |

inline

Definition at line 99 of file trace\_quad\_form.hpp.

template<typename T >

|  |  |  |  |  |  |
| --- | --- | --- | --- | --- | --- |
| fvar<T> stan::agrad::trunc | ( | const fvar< T > & | *x* | ) |  |

inline

Definition at line 15 of file trunc.hpp.

|  |  |  |  |  |  |
| --- | --- | --- | --- | --- | --- |
| var stan::agrad::trunc | ( | const stan::agrad::var & | *a* | ) |  |

inline

Returns the truncatation of the specified variable (C99).

See boost::math::trunc() for the double-based version.

The derivative is zero everywhere but at integer values, so for convenience the derivative is defined to be everywhere zero,

.

Parameters
:   |  |  |
    | --- | --- |
    | a | Specified variable. |

Returns
:   Truncation of the variable.

Definition at line 55 of file trunc.hpp.

template<int R, int C>

|  |  |  |  |  |  |
| --- | --- | --- | --- | --- | --- |
| const Eigen::Matrix<double,R,C>& stan::agrad::value\_of | ( | const Eigen::Matrix< double, R, C > & | *M* | ) |  |

inline

Convert a matrix to a matrix of doubles.

When the input is already a matrix of doubles a reference is returned.

This is used as a convenience function for implementing varis of matrix operations.

Definition at line 16 of file value\_of.hpp.

template<typename T >

|  |  |  |  |  |  |
| --- | --- | --- | --- | --- | --- |
| T stan::agrad::value\_of | ( | const fvar< T > & | *v* | ) |  |

inline

Return the value of the specified variable.

Parameters
:   |  |  |
    | --- | --- |
    | v | Variable. |

Returns
:   Value of variable.

Definition at line 16 of file value\_of.hpp.

|  |  |  |  |  |  |
| --- | --- | --- | --- | --- | --- |
| double stan::agrad::value\_of | ( | const agrad::var & | *v* | ) |  |

inline

Return the value of the specified variable.

This function is used internally by auto-dif functions along with `stan::math::value_of(T x)` to extract the `double` value of either a scalar or an auto-dif variable. This function will be called when the argument is a `stan::agrad::var` even if the function is not referred to by namespace because of argument-dependent lookup.

Parameters
:   |  |  |
    | --- | --- |
    | v | Variable. |

Returns
:   Value of variable.

Definition at line 22 of file value\_of.hpp.

template<int R, int C>

|  |  |  |  |  |  |
| --- | --- | --- | --- | --- | --- |
| Eigen::Matrix<double,R,C> stan::agrad::value\_of | ( | const Eigen::Matrix< var, R, C > & | *M* | ) |  |

inline

Convert a matrix to a matrix of doubles.

When the input is already a matrix of vars a new matrix is returned with the value of the constituent vars.

This is used as a convenience function for implementing varis of matrix operations.

Definition at line 27 of file value\_of.hpp.

|  |  |  |  |  |  |
| --- | --- | --- | --- | --- | --- |
| var stan::agrad::variance | ( | const std::vector< var > & | *v* | ) |  |

Return the sample variance of the specified standard vector.

Raise domain error if size is not greater than zero.

Parameters
:   |  |  |  |
    | --- | --- | --- |
    | [in] | v | a vector |

Returns
:   sample variance of specified vector

Definition at line 51 of file variance.hpp.

template<int R, int C>

|  |  |  |  |  |  |
| --- | --- | --- | --- | --- | --- |
| var stan::agrad::variance | ( | const Eigen::Matrix< var, R, C > & | *m* | ) |  |

Definition at line 68 of file variance.hpp.

## Variable Documentation

memory::stack\_alloc stan::agrad::memalloc\_

Definition at line 16 of file var\_stack.cpp.

std::vector< size\_t > stan::agrad::nested\_var\_alloc\_stack\_starts\_

Definition at line 20 of file var\_stack.cpp.

std::vector< size\_t > stan::agrad::nested\_var\_nochain\_stack\_sizes\_

Definition at line 19 of file var\_stack.cpp.

std::vector< size\_t > stan::agrad::nested\_var\_stack\_sizes\_

Definition at line 18 of file var\_stack.cpp.

std::vector< chainable\_alloc \* > stan::agrad::var\_alloc\_stack\_

Definition at line 15 of file var\_stack.cpp.

std::vector< chainable \* > stan::agrad::var\_nochain\_stack\_

Definition at line 14 of file var\_stack.cpp.

std::vector< chainable \* > stan::agrad::var\_stack\_

Definition at line 13 of file var\_stack.cpp.


---

[ Stan Home Page ]

*© 2011–2014,
Stan Development Team.*
